# Self-assembling proteins compose the chemically resistant shell biomaterial of planktonic tintinnid ciliates

**DOI:** 10.1038/s41467-026-74402-4

**Published:** 2026-06-13

**Authors:** Maximilian H. Ganser, Markus Wiederstein, Christof Regl, Laura A. Katz, Sabine Agatha

**Affiliations:** 1 Department of Environment and Biodiversity, University of Salzburg, Salzburg, Austria; 2 Department of Biosciences, University of Salzburg, Salzburg, Austria; 3 Department of Biological Sciences, Smith College, Northampton, MA USA

**Keywords:** Microbiology, Transcriptomics, Molecular modelling, Biomaterials - proteins, Mass spectrometry

## Abstract

Biomaterials provide superior properties and sustainable alternatives relevant to medicine, textiles, and high-tech applications. Research has mainly focused on animal-derived proteinaceous biomaterials, which remain challenging to reproduce while retaining their remarkable properties. Here, we show that the shell biomaterial of tintinnid ciliates, a lineage of planktonic unicellular eukaryotes, is composed of self-assembling structural proteins. The shells form in sea- and freshwater, are structurally diverse, and exhibit resistance against high temperatures and the strongest chemicals. Combining single-cell transcriptomics with proteomics of the shells, we identify the amino acid sequences of the shell-forming proteins that represent a new family unique to tintinnid ciliates, which we term Tintinnidorin. The proteins are rich in aromatic residues and possess a coherent architecture with flexible, unfolded segments connecting a folded core structure of beta-sheets. These multivalent capabilities facilitate intracellular storage, extracellular self-assembly, wet adhesion, thermostability, and salt tolerance. Tintinnid ciliates and their Tintinnidorin proteins provide an accessible system to elucidate sequence-structure-material relationships and inspire biomaterial design.

## Abstract (web summary)

Tintinnidorin proteins form a resistant material that self-assembles into the shells of tintinnid ciliates, which are unicellular planktonic eukaryotes. The material’s properties make it promising for biomaterial design.

## Introduction

Structural proteins are fundamental building blocks for diverse biological systems, occurring in all lineages across the tree of life and in viruses. Their primary functions include maintaining cellular shape and integrity, enabling movement, and forming protective barriers. Beyond these ubiquitous roles, structural proteins forming complex biomaterials with exceptional mechanical and functional properties have evolved in diverse organisms^1^. A well-known example is silk, a natural biomaterial secreted by various arthropods, including insects and especially spiders, which is renowned for its high tensile strength, elasticity, and biocompatibility^2,3^.

Human use of biomaterials has substantially increased with recent technological progress enabling researchers to mimic various natural properties based on decades of interdisciplinary research^4^. Applications of the resulting synthetic biomaterials are versatile across industries and include textiles^5^, cosmetics, coatings, and wet adhesives^6^. In the medical field, biomaterials are used for tissue engineering, wound dressings, implants, surgical sutures, and drug delivery systems^7^. More broadly, renewable, biodegradable, and sustainable biomaterials offer environmental benefits and economic potential, as demand for green alternatives to petroleum-based materials like conventional plastics continues to grow^8^. While many biomaterials have been discovered in Metazoa^1^, unicellular eukaryotes that comprise the vast majority of eukaryotic diversity^9^ have been largely overlooked.

**Fig. 1:**
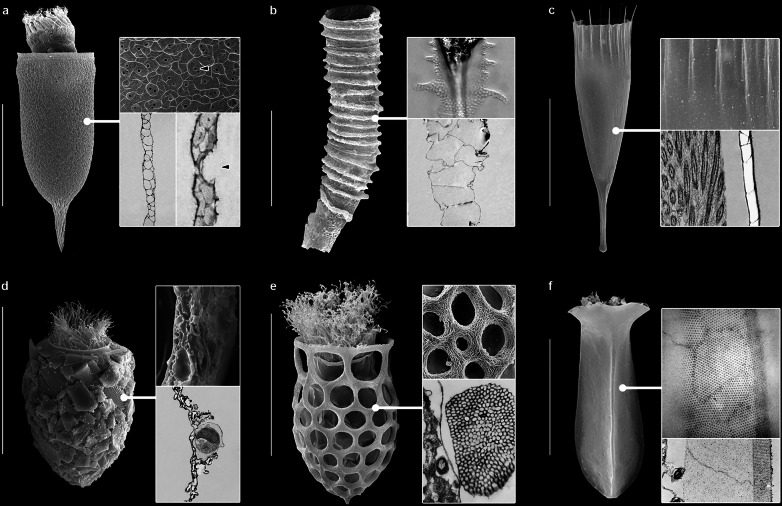
Diversity of shells in tintinnid ciliates. The wall is optically transparent (**a**–**c**, **e**, **f**) or agglutinated (**d**) with chambered (a-d), tubular (e), or solid (f) textures. **a** *Schmidingerella*, shell with protruding cell (SEM, scanning electron micrograph), detail of outer shell surface (SEM), and two ultrathin sections (TEM, transmission electron micrographs) showing wall texture, surface ridges, and minute pores (arrowheads). **b** *Climacocylis*, shell (SEM), detail in the light microscope showing spiralled ridge, and wall section (TEM). Shell image used with permission of John Wiley & Sons, Ltd, from ‘The Biology and Ecology of Tintinnid Ciliates’ (eds Dolan, J. R. et al.), Agatha, S., Laval-Peuto, M. & Simon, P., 2013; permission conveyed through Copyright Clearance Centre, Inc. **c** *Dadayiella*, shell (SEM), detail of anterior outer shell surface (SEM), and wall section (TEM). Shell image reproduced from ‘A comparative ultrastructural study of tintinnid loricae (Alveolata, Ciliophora, Spirotricha) and a hypothesis on their evolution’, Agatha, S. & Bartel, H., Journal of Eukaryotic Microbiology 69, 2022, according to the CC BY 4.0 requirements. **d** *Stenosemella*, shell (SEM), fracture surface (SEM), and wall section (TEM) with adhered and imbedded foreign particles. **e** *Dictyocysta*, shell (SEM), detail of outer surface (SEM), and wall section (TEM^109^). Shell image reproduced from ‘A light and scanning electron microscopic study of the closing apparatus in tintinnid ciliates (Ciliophora, Spirotricha, Tintinnina): a forgotten synapomorphy’, Agatha, S., Journal of Eukaryotic Microbiology 57, 2010; permission conveyed through Copyright Clearance Centre, Inc. Image of shell section used with permission of Elsevier Science & Technology Journals, from ‘Traité de Zoologie. Infusoires Ciliés, Systématique’ (ed P. de Puytorac), Laval-Peuto, M., 1993; permission conveyed through Copyright Clearance Centre, Inc. **f** *Amphorides*, shell (SEM), tangential section of outer wall layer and transverse section showing the solid wall with a crystalline outer and an amorphous inner layer (TEM). Shell image reproduced from ‘A comparative ultrastructural study of tintinnid loricae (Alveolata, Ciliophora, Spirotricha) and a hypothesis on their evolution’, Agatha, S. & Bartel, H., Journal of Eukaryotic Microbiology 69, 2022, according to the CC BY 4.0 requirements. Images are either original, owned by the authors, or reproduced with permission (Supplementary Note 8). Original SEM, TEM, and light microscope observations were confirmed in at least three independent specimens. Scale bars 100 µm (**a**, **b**), 50 µm (**c**–**f**).

Tintinnids are microscopic shell-forming ciliates usually 50–400 µm long that have inhabited the marine plankton since about 260 mya^10^. Each of the about 1000 extant species distributed across the world’s oceans and freshwater bodies forms a distinct vase- or tube-shaped shell (lorica)^11,12^. Tintinnid shells are intricate structures, rich in texture and ornamentation^11,13^, resembling delicate works of art, yet crafted by a single cell (Fig. 1). The proposed adaptive advantages of the shells for tintinnid ciliates are primarily associated with protection and efficient filter feeding (Supplementary Note 1). Structurally, the shells may be agglutinated with foreign particles (e.g., *Tintinnopsis* species), entirely transparent (e.g., *Schmidingerella* species), or a combination of both (e.g., *Codonellopsis* and *Stenosemella* species). The shell walls can be soft (e.g., *Tintinnidium* and *Antetintinnidium* species) or rigid (remaining tintinnids) and may feature minute pores (e.g., *Schmidingerella*) or large fenestrations (e.g., *Dictyocysta*). At the ultrastructural level, the shell walls consist of one or multiple layers, which exhibit various organizational patterns including chambered, tubular, solid, or crystalline textures^13^. Previous experiments demonstrated the resilience of the shell material to various proteolytic enzymes as well as strong acids and bases even under high temperatures^14,15^ (Supplementary Note 2).

The chemical composition of tintinnid shells has remained a mystery for over two centuries^14,15^. The shell-forming material is produced during the cell cycle and accumulates as secretory granules that progressively mature^16^. Hardly anything is known about shell construction by tintinnid ciliates because the process has rarely been observed or documented, until now. In species forming transparent shells, the process is usually completed within a few minutes after cell division and involves a coordinated interplay of regulated granule secretion, cell movement, and self-assembly of the shell-forming granular material^16,17^.

**Fig. 2:**
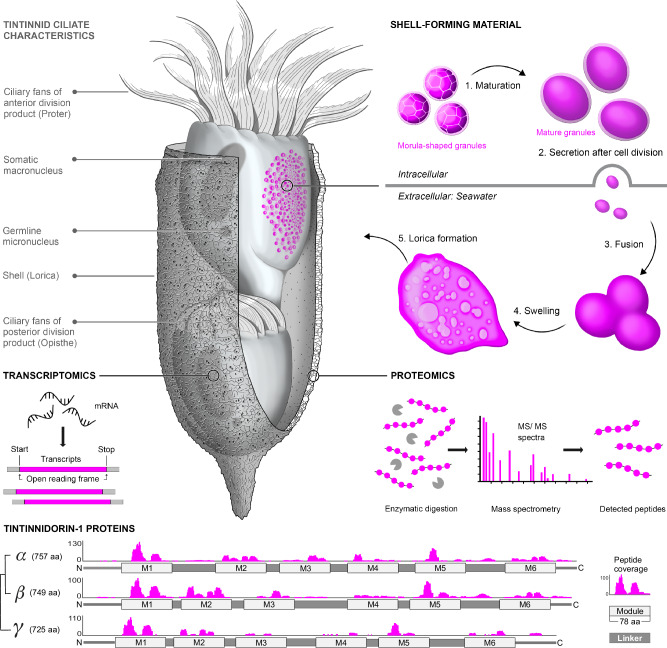
Identification of shell biomaterial proteins in the tintinnid ciliate *Schmidingerella.* Transcriptomic and proteomic analyses on cells and shells congruently identified three variants of a new protein family, namely, Tintinnidorin-1-alpha, beta, and gamma. Gene-sized nanochromosomes encode Tintinnidorin proteins in the macronucleus. During the complex cell division process, messenger RNA (mRNA) is translated into Tintinnidorin proteins that form microscopically visible granules in secretory vesicles. The intracellular granular shell material matures and is secreted by the anterior division product after its separation from the posterior division product, which keeps the parental shell about 200 × 80 µm in size. The rapid self-assembly process involves the fusion and swelling of the extracellular granules, eventually forming the shell of the tintinnid steadily swimming in the water column by means of its apical ciliary fans (membranelles; Supplementary Note 3). The three Tintinnidorin protein variants comprise six modules of identical length alternating with linkers of highly variable lengths. The short chains of amino acids (peptides) identified in the tandem-mass spectrometry data of enzymatically digested *Schmidingerella* shells mainly cover the modules of the three Tintinnidorin protein variants; their quantity is depicted by magenta curves parallel to the protein sequences. Pairwise comparisons reveal a higher sequence similarity between Tintinnidorin-1-alpha and beta as illustrated in the tree. aa, amino acids; C, C-terminal region; N, N-terminal region. Images of tintinnid ciliate and shell-forming granules created by Michael Gruber (Salzburg) based on detailed information given by the authors.

This study identifies and characterises structural proteins as the main component of tintinnid ciliate shells by combining single-cell transcriptomic analyses and mass-spectrometry of the shells from the tintinnid *Schmidingerella* (Fig. 2). We incorporate single-cell transcriptome data from further tintinnid species with structurally diverse shells (this study; ref. ^18^), including agglutinated forms, and metatranscriptome and genome data from plankton samples collected globally^19,20^. The identified shell proteins exhibit a coherent architecture and composition distinct from previously characterised proteins in reference databases. Their apparent restriction to tintinnid ciliates supports the establishment of a new protein family, which we designate Tintinnidorin. Further, we describe potential mechanisms of the shell material self-assembly and discuss the use of tintinnid ciliates as convenient systems to uncover protein sequence-structure-function relationships that will benefit the design of next-generation biomaterials.

## Results and discussion

### Identification of the shell biomaterial proteins

We obtained single-cell transcriptome data from a monoclonal culture of the tintinnid *Schmidingerella*, including 20 individuals in early, middle, late, and post-division stages of the cell cycle. The transcriptome data from each individual (cells 1–20; Supplementary Data 1) as well as the pooled data from all cells were assembled de novo because no complete reference genome or transcriptome exists. The pooled data assembly, termed the “*Schmidingerella* reference transcriptome”, represents our best inference of protein-coding sequences as it comprises the most comprehensive proteome coverage of a tintinnid ciliate’s cell cycle to date. Additionally, to determine their composition, we enzymatically digested about 1700 of the transparent, champagne flute-shaped shells from the same *Schmidingerella* culture with a custom protocol (Supplementary Methods 3 and 4). The composition of the shells was assessed after tandem-mass spectrometry. We used a machine learning model^21^ to identify potential shell peptide sequences from the mass spectra de novo, that is, without reference databases of known peptides or any prior information on the protein sequences. We validated the identified shell peptide sequences (Fig. 2) by manual assignment of the mass spectra (Supplementary Data 2).

Combining transcriptomic and mass spectrometry data enabled direct identification of the shell proteins by their full-length amino acid sequences. Peptides from the digested shells that were predominant in several independent tandem-mass spectrometry measurements matched various segments of three full-length protein-coding sequences (757, 749, and 725 amino acids in length) of the *Schmidingerella* reference transcriptome (Fig. 2). Two further features of the identified protein sequences provide direct links to the shell-forming material production and secretion in our *Schmidingerella* cells. First, the messenger RNAs (mRNAs) encoding these protein sequences are more highly expressed in the late stages of *Schmidingerella*’s cell division than in early and middle division stages (Supplementary Fig. 1 and Supplementary Data 3). Thus, the gene expression levels mirror previous light microscopical observations of cells from the same culture, in which the largest increase of shell-forming material was quantified in late cell cycle stages^22^. Secondly, the three protein variants each contain a eukaryotic signal peptide for extracellular transport at the N-terminus determined by protein language model predictions^23^ (Supplementary Data 4).

**Fig. 3:**
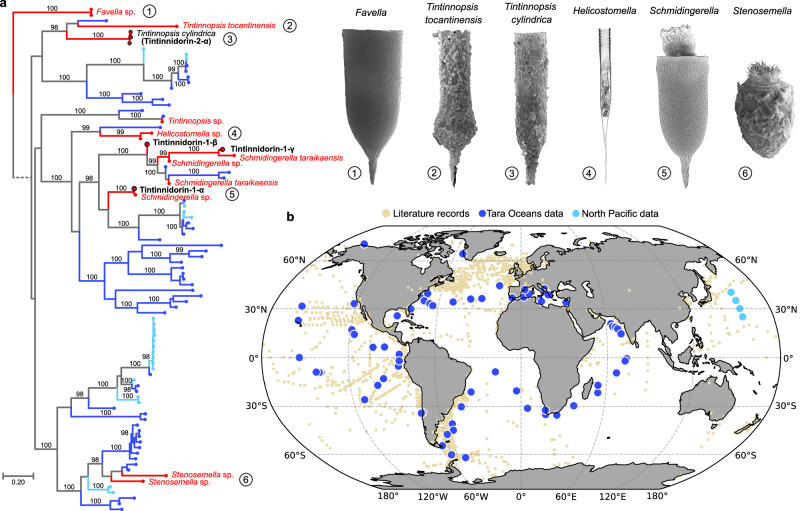
Tintinnidorin proteins extracted from single-cell transcriptomes and discovered in metatranscriptome data from global ocean plankton surveys. **a** Phylogeny of Tintinnidorin proteins. Single-cell data annotated with taxon names, light microscopic (4) and scanning electron microscopic images (1–3, 5, and 6) comprise full-length sequences (black) of *Schmidingerella* (Tintinnidorin-1-alpha, beta, and gamma), *Tintinnopsis cylindrica* (Tintinnidorin-2-alpha), and various partial sequences (red). The shells of *Favella*, *Helicostomella*, and *Schmidingerella* are optically translucent, while those of *Tintinnopsis* and *Stenosemella* are agglutinated with mainly mineral particles. The full-length Tintinnidorin sequences from the Tara Oceans data (dark blue) and the North Pacific Eukaryotic Gene Catalog (light blue) are not linked with morphospecies. Scale bar indicates substitution rate. Bootstrap support values (≥ 98) are shown on branches. **b** Global distribution of tintinnid ciliates based on the identification of their shells (literature records) and Tintinnidorin proteins.

We detected homologues of the identified shell proteins in single-cell transcriptomes of several other tintinnid species (Supplementary Data 5). The shell proteins clustered in a single orthogroup that only contained sequences from tintinnid ciliates (Supplementary Fig. 2). In contrast, no further sequence homologues were found in an extensive dataset of 1000 genomes and transcriptomes from a wide variety of archaea, bacteria, and eukaryotes^24^ including tintinnid-related planktonic ciliates without shells. Furthermore, structure searches against comprehensive sets of experimentally determined as well as predicted protein structures did not reveal significant structure similarities to known proteins (Supplementary Fig. 3 and Supplementary Data 6). The results strongly suggest that the structural proteins forming the shell biomaterial exclusively evolved in tintinnid ciliates. Here, we term the newly discovered protein family “Tintinnidorin” (Supplementary Note 4). Screening the Tara Oceans data in the Ocean Gene Atlas^25^ and the North Pacific Eukaryotic Gene Catalog^20^ revealed an extensive phylogenetic diversity of Tintinnidorin proteins greatly expanding our single-cell dataset (Fig. 3 and Supplementary Data 7).

### Characteristics of Tintinnidorin proteins

Tintinnidorin protein characteristics are the foundation for the multifaceted features and diversity of tintinnid ciliate shells. Our insights into these characteristics are based on in silico analyses that mainly cover amino acid composition, sequence architecture, and predicted protein structures. Physicochemical mechanisms and properties deduced from these analyses elucidate features, such as structural resilience, the capability for wet adhesion, and self-assembly, which follow general principles also associated with diverse terrestrial and aquatic animal biomaterials^26–29^.

**Fig. 4:**
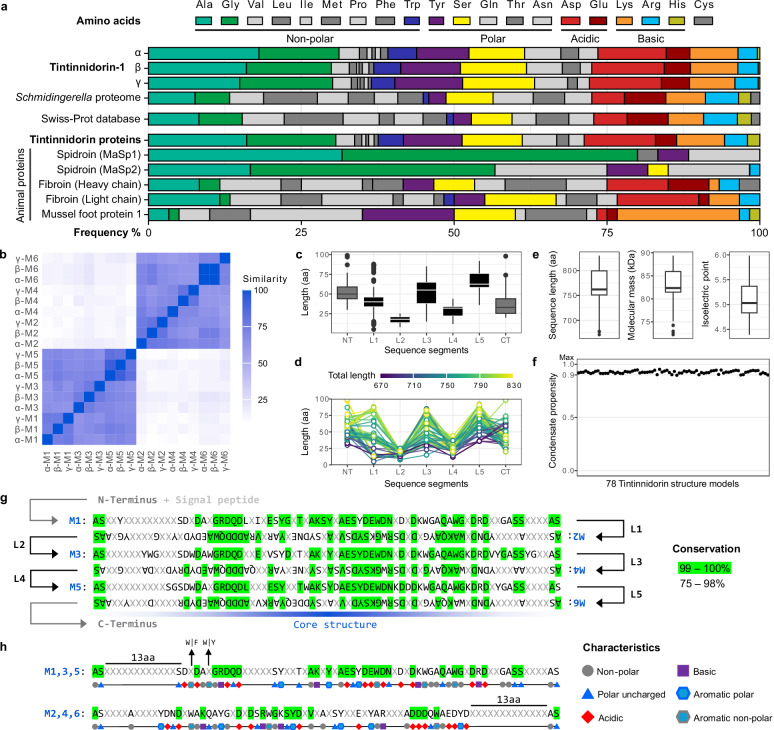
Comparisons of amino acid compositions. **a** Amino acid frequencies in Tintinnidorin-1-alpha, beta, and gamma proteins and their average values in the *Schmidingerella* proteome, the Swiss-Prot database, the Tintinnidorin protein sequences (*n* = 78), and selected animal biomaterial proteins (spider major ampullate spidroins 1 and 2; silkworm fibroin heavy and light chains; mussel foot protein 1). **b** Pairwise module sequence similarity of Tintinnidorin-1-alpha, beta, and gamma from *Schmidingerella*. **c**,** d** Sequence length distributions of N-terminal (NT), linker (L1–5), and C-terminal segments in the Tintinnidorin sequences (*n* = 78). Lineplot colours indicate total sequence length. **e** Boxplots of sequence lengths, molecular masses, and isoelectric points of the Tintinnidorin proteins (*n* = 78). Boxplots show the median (black/white centre line), the interquartile range (box; 25th–75th percentiles), and whiskers extending to the most extreme values. Outliers beyond this range are plotted as points. Minimum, maximum, and percentile values were calculated accordingly. **f** Condensate (phase separation) propensity values predicted for the Tintinnidorin structure models (*n* = 78). **g** Consensus of the six modules among the Tintinnidorin protein sequences (*n* = 78). Conserved amino acids present in ≥ 99% of sequences marked by green, amino acids present in 75–98% of sequences are denoted by their one-letter code, while variable amino acids are indicated by X. **h** Consensus amino acid sequences of odd- and even-numbered modules in the Tintinnidorin sequences (*n* = 78), respectively. Conserved amino acids present in ≥ 99% of sequences are marked by green, amino acids present in 75–98% of sequences are denoted by their one-letter code and annotated with their characteristics, while variable amino acids are indicated by X. α, β, γ Tintinnidorin-1-alpha, beta gamma; aa, amino acids; CT, C-terminal segment; L1–5, linker segments 1–5; M1–6, modules 1–6; NT, N-terminal segment. Amino acids: A, Ala, alanine; G, Gly, glycine; V, Val, valine; L, Leu, leucine; I, Ile, isoleucine; Met, methionine; Pro, proline; F, Phe, phenylalanine; W, Trp, tryptophan; Y, Tyr, tyrosine; S, Ser, serine; Q, Gln, glutamine; T, Thr, threonine; N, Asn, asparagine; D, Asp, aspartic acid; E, Glu, glutamic acid; K, Lys, lysine; R, Arg, arginine; His, histidine; Cys, cysteine.

Tintinnidorin protein compositions generally deviate from those of proteins in the Swiss-Prot database^30^ (Fig. 4a) and the other protein sequences in the *Schmidingerella* reference transcriptome due to high fractions of alanine, glycine, aspartic acid, tyrosine, and tryptophan (Fig. 4a and Supplementary Data 8). While the remaining amino acids are present in varying amounts, cysteine is completely absent in Tintinnidorin-1 proteins of *Schmidingerella* (Supplementary Fig. 4) and rarely existent in those of the other tintinnid species analysed so far (Supplementary Data 8). The most frequent amino acids in Tintinnidorin proteins are similarly predominant in different components of terrestrial and aquatic animal biomaterials. Alanine and glycine are the most prevalent amino acids in the main structural proteins of terrestrial animal derived silks, such as spider spidroins^1,31^ and silkworm fibroins^32^. Tyrosine and lysine are abundant in proteins of marine animal biomaterials that are related to wet adhesion, such as mussel byssal foot proteins^33^ and sandcastle worm tube cement^27^ (Supplementary Note 2). Aspartic acid mainly occurs in protein components of fibroin-derived silks produced by terrestrial and aquatic animals^1^.

Tryptophan, one of the rarest amino acids in eukaryotes^34^, occurs in Tintinnidorin in much higher amounts (3.5–4.7%) than in known proteinaceous biomaterials and most proteins in general (Fig. 4a and Supplementary Data 8). The abundances of the aromatic amino acids tyrosine and tryptophan suggest that the shell biomaterial exhibits photoprotective properties by effectively absorbing ultraviolet radiation. In fact, we observed autofluorescence of *Schmidingerella* shells and transparent shells of other tintinnid species excited with UV light (Supplementary Fig. 5; excitation at 385 nm, emission at about 465 nm). Although excitation and emission wavelengths of these amino acids are typically much shorter (280 nm and 295–400 nm, respectively)^28,35^, several processes, e.g., the formation of bonds, oxidation, and structural features of the biomaterial, could result in a red edge excitation/emission shift^36^.

In terms of sequence architecture, Tintinnidorin proteins consist of six regions with an identical number of amino acids (modules M1-6) alternating with five regions of varying lengths (linkers L1-5) between the N- and C-terminal ends (Fig. 2—bottom and Supplementary Fig. 6). The linkers are between 5 and 91 amino acids in length (Fig. 4c, d and Supplementary Data 9), whereas the modules are invariably 78 amino acids long and mainly flanked by pairs of alanine and serine, sometimes aspartic acid or rarely other residues (Fig. 4g). Compositionally, modules and linkers differ significantly in their most abundant amino acids glycine, tryptophan, serine, aspartic acid, glutamic acid, lysine, arginine, and histidine (Supplementary Fig. 4c, d and Supplementary Data 10–12). This pattern of modules and linkers distinctly deviates from known structures of animal silk proteins, which mainly comprise low-complexity sequence regions rich in glycine and alanine and arranged in tandem repeats between the compositionally more diverse N- and C-terminal domains^1^.

Tintinnidorin modules are not identical in their amino acid sequences but show an alternating pattern with low similarity between pairs of subsequent modules (14 ± 3%) and high similarity between pairs of odd-numbered (70 ± 6%, M1, 3, 5) or even-numbered (69 ± 8%, M2, 4, 6) modules (Fig. 4b and Supplementary Data 13). Within odd- and even-numbered modules, about 35% and 21% of the amino acid positions are conserved across all Tintinnidorin sequences, respectively, and thus represent distinct sequence motifs (Fig. 4g, h). In contrast, the linkers contain no generally conserved positions, but a mixture of partly repeating amino acid segments mainly composed of glycine, tyrosine, lysine, alanine, and histidine. Additionally, linkers display a significantly higher variance of most amino acids compared to the modules (Supplementary Fig. 4c and Supplementary Data 10). Usually, two to four proline residues are present in each of the N- and C-terminal segments, whereas about two thirds of the Tintinnidorin proteins contain single proline residues in one or more of their linkers. The near absence of linker peptides in the proteomic analyses (Fig. 2—bottom) suggests that shell digestion by the three proteases either produced linker fragments below the detection limit, or alternatively, that covalent crosslinks impeded proteolytic cleavage, resulting in large and heterogeneous peptides unsuitable for mass spectrometry (Supplementary Method 5). Lengths of linkers 1, 3, and 5 vary widely, whereas those of linkers 2 and 4 show less variation in *Schmidingerella* and the other Tintinnidorin sequences. On average, linkers 2 and 4 are also consistently shorter than linkers 1, 3, and 5 (18 and 29 vs. 42, 54, and 66 amino acids) (Fig. 4c and Supplementary Data 9).

The emergence of conserved and variable elements in Tintinnidorin proteins is most likely related to the unusual genome architecture of ciliates, a lineage defined by the presence of both a germline micronucleus and somatic macronucleus in every individual^37^. One of the three Tintinnidorin variants of *Schmidingerella*, Tintinnidorin-1-gamma, appears to be a canonical paralog as it is divergent from the other two across its full length. In contrast, Tintinnidorin-1-alpha and beta are roughly 30% divergent across two thirds of their lengths; the remaining portions of these two sequences are identical (Supplementary Fig. 2). This pattern is consistent with alternative processing of shared germline sequences^38,39^, a process that is associated with elevated rates of protein evolution in ciliates as compared to other eukaryotes^40,41^. An alternative explanation/hypothesis is that gene conversion has homogenized the 3’ ends of Tintinnidorin-1-alpha and beta. Due to the lack of a complete *Schmidingerella* reference genome including micronuclear and macronuclear data, and of tintinnid genomic data in general, the evolutionary mechanisms remain elusive (Supplementary Note 5).

**Fig. 5:**
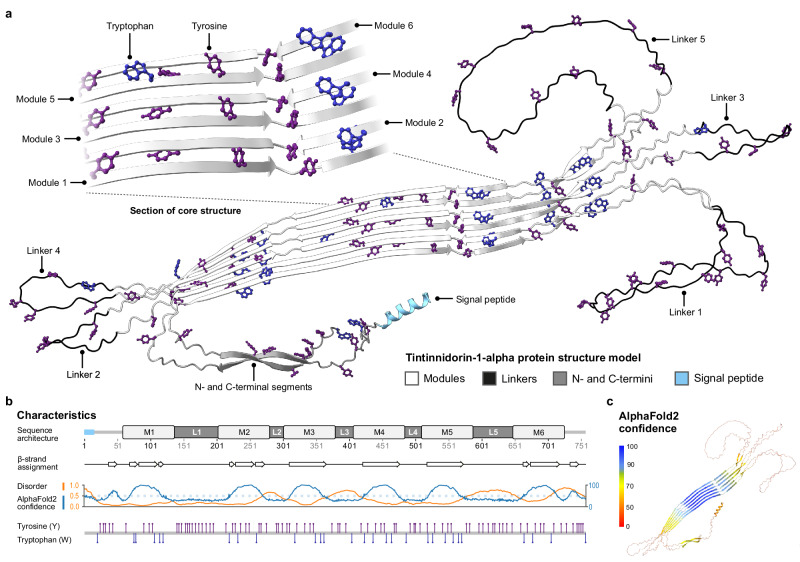
Predicted protein structure of Tintinnidorin-1-alpha from *Schmidingerella.* Six antiparallel beta-sheets mainly comprising the modules form the core structure (**a**) with high support by beta-strand assignments by STRIDE (**b**) and AlphaFold2 confidence values (**b**, **c**; coded by blue). The modules 1–6 (M1-6) are connected by linkers 1–5 of variable lengths, which display high values of disorder (**b**) as predicted by metapredict V2, probably causing a high conformational heterogeneity and low model confidences (**b**, **c**; coded by orange). The aromatic amino acids tyrosine (Y; purple) and tryptophan (W; blue) are highly abundant and rather homogenously distributed across the protein (**a**, **b**). The signal peptide for extracellular transport precedes the N-terminal segment, which forms intertwined beta-strands with the C-terminal segment.

Protein structure predictions of the 78 full-length Tintinnidorin sequences by AlphaFold2^42^ consistently resulted in monomers with strikingly similar core structures (Supplementary Figs. 8 and 9). The core structure comprises the six modules that form a layer of antiparallel beta**-**sheets connected by the linkers that lack a stable, well-defined three-dimensional structure, indicating that they are intrinsically disordered (Fig. 5a). Similarly, the terminal regions, especially the C-terminus, show a high propensity for disorder (Supplementary Fig. 10). However, the N- and C-terminal segments form two intertwined beta-strands in most monomer models, suggesting these sites to interact with other monomers (Fig. 5a). The predicted protein structures with the module-linker pattern are supported by secondary structure assignments by STRIDE^43^, disorder predictions by metapredict V2^44^, and the AlphaFold2 local confidence scores (Fig. 5b, c and Supplementary Fig. 6).

Sequence and structural characteristics of Tintinnidorin proteins are consistent with features displayed by the shell-forming material. Tintinnidorin proteins universally show a high condensate-forming potential, which means that they very likely undergo liquid-liquid phase separation (LLPS; scores ≥0.9, on a scale of 0–1) (Fig. 4f and Supplementary Data 7). Structural transitions of the shell-forming material during intracellular maturation revealed by transmission electron microscopy^16^ are probably the result of phase separation (Fig. 2). LLPS is strongly associated with storage and self-assembly processes in many protein-based materials of animals and pathogenic amyloids in humans^1,26,45,46^. These findings indicate that LLPS plays an important role for structural proteins in completely different biological systems (Supplementary Note 6). The flexibility of Tintinnidorin proteins required for undergoing LLPS and gelation is most likely achieved by the intrinsic disorder of their linkers (Supplementary Fig. 10). Actually, computational simulation experiments suggest that the properties of the intrinsically disordered linker regions, such as length and amino acid-dependent expansion or compaction in solution, can actively influence phase separation and gelation processes^47^.

Intracellular shell-forming material is stored in secretory vesicles (Fig. 2) that are strongly eosinophilic as revealed by histological stains of *Schmidingerella*^22^, indicating acidic conditions (low pH ~4–5)^48^. Tintinnidorin proteins exhibit low isoelectric points (mean pI = 5.1; Fig. 4e and Supplementary Data 7), resulting in near neutral net charges at low pH, which promotes protein-protein interactions due to reduced solubility and thus stable storage conditions. To concentrate and store functional biomaterial proteins within small volumes, reversible bonds might be formed by stickers (tryptophan, tyrosine, phenylalanine, and histidine residues) that are interspersed between flexible spacers (glycine and alanine residues) according to the sticker and spacer model^49^. In the Tintinnidorin proteins, the most potent “sticker” amino acids, namely, tryptophan and tyrosine, are abundant and evenly distributed (Fig. 5a, b and Supplementary Fig. 6), while flexible “spacer” amino acids are mainly present in the linkers (Supplementary Fig. 4d). Thus, interactions between the linkers and the modules under acidic conditions might enable densely packed proteins in the secretory vesicles, avoiding formation of irreversible and harmful assemblies similar to amyloid peptide aggregations^50^.

Extracellular formation of antiparallel beta-sheets contributes significantly to the mechanical strength and stability of structural proteins in silk biomaterials^51^. In spiders and silkworms, threads are spun from protein fibres, in which beta-sheet formation is mediated by shear forces, pH, and ion concentration specifically modulated in the silk-producing organs^26,52,53^. Likewise, mussels form proteinaceous byssus threads in a groove of their foot by controlling the microenvironment^29,54^. In comparison to these animals, tintinnid ciliates cannot actively control the extracellular physicochemical environment during shell formation as the shell-forming material is rapidly secreted into the surrounding (sea-)water. Nevertheless, family and genus-specific shell characteristics are consistent across widely differing environmental conditions. In some genera, species have even been recorded from seawater, brackish water, and freshwater. How Tintinnidorin proteins reliably self-assemble despite such contrasting conditions remains an open question (Supplementary Note 7).

In many marine prokaryotes, secreted proteins typically have low isoelectric points reflecting adaptations that prevent their denaturation even at seawater salinities^55^. To our knowledge, Tintinnidorin proteins represent the first example of extracellular eukaryotic proteins that follow this principle to retain their self-assembly function (Fig. 4e). Aromatic residues apparently play an important role for the thermostability of proteins^56^. In the seawater habitat of *Schmidingerella*, the extracellular conditions are different from the conditions in the secretory vesicles regarding pH (~8.1), ion composition, and oxidation potential. We observed a fusion and gradual increase in volume (swelling) of the shell-forming material granules (Fig. 2 and Supplementary Movie 1), which are in a viscous gel-like state just after secretion. The hardening of the shell marks the biomaterial’s transition to a solid state, which results in the characteristic chambered texture of the *Schmidingerella* shell wall with its surface ridges and pores (Fig. 1a). Gelation and solidification during shell formation are most likely driven by finely tuned phase transitions including liquid-liquid phase separation (LLPS).

### Global distribution of Tintinnidorin proteins

Tintinnidorin proteins are globally distributed and may serve as a marker to systematically assess the biogeography of tintinnid ciliates in future studies. Commonly, the global distribution of tintinnid ciliates has been inferred from their taxonomically relevant shells collected in plankton samples for about 250 years^57^ (Fig. 3b). In recent decades, molecular data, such as ribosomal marker genes, have significantly enhanced the resolution of tintinnid biodiversity studies through integrative approaches^12,58^. We detected Tintinnidorin protein sequences in the Tara Oceans metatranscriptome data acquired from each of the 68 globally scattered sampling sites^19^ and in the North Pacific Eukaryotic Gene Catalogue^20^ (Fig. 3b and Supplementary Data 14). While the latter comprises samples from a transect extending from the North Pacific Subtropical Gyre to the North Pacific Transition Zone, the Tara Oceans data cover most major oceanic provinces, oceanic and neritic regions, and different water depths (0–1000 m)^59^. These datasets contained 72 full-length Tintinnidorin protein sequences (from start to stop codon) plus many partial sequences (Tara = 370, Pacific = 1662) that mainly originate from samples taken from the ocean surface layer or at the deep chlorophyll maximum (0–200 m depth). Taxonomic annotations of the unigenes from the Tara Oceans data to the lowest possible rank^19^ that we identified as Tintinnidorin sequences show a strong association with ciliates (92%; *n* = 47), and specifically with tintinnid ciliates (45%, *n* = 23; Supplementary Data 7). We note that the approach depends on curated reference databases, which are incomplete, limiting the taxonomic resolution of metatranscriptome sequence annotations. Incomplete recovery of full-length Tintinnidorin transcripts from (meta-)transcriptomes is most likely related to low expression levels, partially degraded mRNA, sequencing bias, and challenges of a de novo assembly without reference genomes or transcriptomes, which are common limitations^60^. Nonetheless, even partial protein sequences could be unequivocally assigned to Tintinnidorin owing to its distinct sequence motifs.

Abundances of tintinnid ciliates vary substantially, from fewer than 100 to over 70,000 individuals per litre, accounting for up to 20% of the total biomass of microzooplankton (20–200 µm in size) communities^61^. One portion of this biomass can be attributed to Tintinnidorin proteins composing the shells, which remain completely intact even after digestion by predators, such as planktonic invertebrates feeding on tintinnid ciliates^62^. However, the fate of the shells after the decline of a tintinnid population (up to 500,000 sedimenting shells m^–2^ d^–1^)^63^ and their importance for biogeochemical recycling remain largely unknown. Sparse observations indicate that bacterial degradation may contribute to the breakdown of the tintinnid shell biomaterial during sedimentation or at the bottom of the oceans^64,65^.

### Potential of Tintinnidorin as a model system for biomaterial research

Tintinnid ciliates generate an astounding diversity of shells from Tintinnidorin proteins that unite a multitude of remarkable features. Investigating how these features are linked to the distinct characteristics of the shells from molecular to microscopic scales could provide essential insights into protein sequence-structure-function relationships. While general principles of such relationships have been extensively studied in animals^1,26,45^, these complex systems remain inherently challenging, and mimicking biomaterials, such as silk, with comparable mechanical and functional properties has yet to be achieved^66^.

The potential of the tintinnid shell biomaterial has been demonstrated by experiments and observations revealing remarkable properties, such as thermal and chemical resistance, photoprotection, wet adhesion, and self-assembly (Supplementary Note 2). On the molecular level, Tintinnidorin proteins exhibit architectures that could serve as a convenient model complementing current biomaterial research. The shell biomaterial provides unique insights into the hierarchical self-assembly of the proteins into diverse three-dimensional layers in water. In contrast, the mostly terrestrial fibre-forming silk biomaterials are complex mixtures of proteins produced by specialized cells of dedicated spinning organs^67–70^. How Tintinnidorin proteins interact and finally form a multimeric protein complex in the shell biomaterial will be the aim of future studies. At present, our analyses are restricted to single protein models, as predicting interactions among structural proteins while accounting for changing intracellular and extracellular conditions exceeds current methodological capabilities. In comparison to animals, tintinnid ciliates as single-celled organisms have distinctly shorter generation times (commonly daily cell divisions) and thus rather continuously produce Tintinnidorin proteins. Further advantages are that the cells are directly accessible to staining techniques of subcellular organelles and structures for advanced light and electron microscopic imaging and are potentially suitable targets for genetic engineering.

## Methods

### Sampling and cultivation

Monoclonal cultures of the model tintinnid *Schmidingerella* established with specimens collected from the Rosario Strait, Northeast Pacific, were provided by Kelley Bright and Suzanne L. Strom from the Shannon Point Marine Center, Western Washington University, USA, in November 2022. Details on the conditions used to establish cultures of *Schmidingerella* in the Strom Lab are provided in Supplementary Method 2. The monoclonal cells investigated in the present study (SPMC 176) were maintained at our laboratory for several months in artificial seawater plus f/2 medium^71^ at a salinity of 32‰, a pH of 7.8, a temperature of 15 °C, and a light-dark-cycle of 12:12 h. As food items, the dinoflagellate *Heterocapsa triquetra* (CCMP 448) and the haptophyte *Isochrysis galbana* also provided by the Strom Lab were maintained in culture as well. Each well of 6-well plates (CELLSTAR^®^ 6 Well Cell Culture Plates) was filled with 10 ml of filtered ciliate medium and about 5 ml of *H. triquetra* culture and kept for one day in the culture cabinet. Every two or three days, 20–40 *Schmidingerella* cells were transferred into the wells of the prepared plates. Finally, one drop of *I. galbana* culture was added to each well.

Field material sampling and single-cell processing of other tintinnid ciliates (*Favella*, *Tintinnopsis cylindrica*, *T. tocantinensis*, and *Stenosemella*) was performed by colleagues according to methods described in ref. ^18^. Further tintinnid ciliates were collected by a 20-µm-meshed plankton net from surface waters at Avery point, Groton, CT, USA (*Helicostomella*; 41°18'59.2“N 72°03'38.1“W) and Megan-sett Harbor, North Falmouth, MA, USA (*Metacylis* and *Schmidingerella*; 41°39'22.9“N 70°37'24.8“W).

### Fluorescence microscopy

Shells of *Favella*, *Helicostomella*, *Metacylis*, and *Schmidingerella* (Avery Point and Megansett Harbor field samples) were analysed with a Zeiss Axio Imager M2 microscope equipped with a Zeiss ApoTome.2 device at Smith College in Northampton, MA, USA. Images were captured with a Zeiss Axiocam 503 mono digital camera and the ZEN software v3.8 (Zeiss) at 200× and 400× magnifications and an excitation wavelength of 385 nm (UV light).

### Single-cell transcriptomics of *Schmidingerella*

#### Isolation of cells

To reduce contamination by non-target organisms, *Schmidingerella* specimens (*n* = 200) from the monoclonal culture were picked in batches of about 20 cells with a finely drawn glass Pasteur pipette and transferred three times to fresh wells of 6-well culture plates containing exclusively artificial seawater. Cells were either directly processed or starved for 10 min to 24 h. Next, single cells were transferred to a drop of artificial seawater on a disinfected microscope slide to determine the division stage under an Olympus BX53 compound light microscope with differential interference contrast optics at 200–400× magnification without a cover slip.

#### Cell classification

Assignment of specimens to division stages followed the definitions in ref. ^22^. The main distinguishing feature was the shape of the oral primordium (new oral apparatus of the posterior division product): small non-divider without oral primordium or early divider with a very small one (ED); middle divider with crescent-shaped oral primordium (MD); late divider with circular oral primordium parallel to cell surface (LD); and very late divider with oral primordium in distinct division furrow (VLD; Fig. 2). The category ‘postdivider’ (PD) represents posterior division products (opisthe cells) picked just after cell division (cell 9; Supplementary Data 3) and cells apparently missing a shell, which could include anterior division products (proters) that were unable to form a complete shell just after division or that abandoned their shells (cell 1).

#### mRNA extraction, amplification, library preparation, and sequencing

The SMART-Seq® v4 Ultra® Low Input RNA Kit (TaKaRa Bio, USA) was used for single-cell mRNA extraction, transcription, and subsequent cDNA amplification^72^. Each classified cell was transferred in about 1 µl of artificial seawater to a PCR tube, to which 0.25 µl 10× reaction buffer and 1.15 µl nuclease-free water were added immediately (total volume ~2.4 µl). PCR tubes containing the cells were frozen at –80 °C. Twenty out of the 200 picked *Schmidingerella* cells covering each division stage plus postdividers (ED = 2, MD = 10, LD = 3, VLD = 3, PD = 2) were randomly selected and processed, following the manufacturer’s protocol, except for using merely one fourth of the recommended reaction volumes. Samples were purified, using the AMPure XP purification system (Beckman Coulter, USA) and quantified with a Qubit 3.0 Fluorometer (Thermo Fisher Scientific, USA). Sequencing libraries were prepared with the Nextera XT DNA Library and Nextera XT Index kits (Illumina, USA). Paired-end sequencing (PE150) was conducted on an Illumina NovaSeq6000 System at Biomarker Technologies GmbH (Münster, Germany).

#### Raw read processing and transcriptome assembly

The quality of raw reads from each *Schmidingerella* single-cell transcriptome was assessed with FastQC v0.12.1^73^, and results were compiled with MultiQC v1.15^74^. Sequencing adapters and primers from cDNA amplification were removed. Reads were quality-filtered (phred score threshold = 24) and trimmed to a minimum length of 100 base pairs with BBduk from the BBMap package v39.28^75^. Next, reads were processed with RiboDetector v0.3.1^76^ to identify ribosomal RNA (rRNA) sequences (-e rrna), which were removed and stored in separate FASTQ files (Supplementary Method 3). De novo transcriptome assemblies were generated for each cell, using SPAdes v3.15.5 with the rnaSPAdes mode enabled (--rna) and k-mer sizes of 21, 33, 55, and 77^77,78^. In addition, reads from all twenty cells were pooled and assembled, using the same parameters to generate a reference dataset of protein-coding genes across the cell cycle of *Schmidingerella* termed the “reference transcriptome”. Raw read processing and assembly metrics are summarized in Supplementary Data 1.

#### Identification of protein-coding regions

Open reading frames (ORFs) in the assembled mRNA transcripts were identified with TIdeS v1.1.2^79^ (https://github.com/xxmalcala/TIdeS), which employs a machine learning approach. The TIdeS (Transcript Identification and Selection) framework utilizes a classifier trained on features such as coding potential, sequence composition, and ORF length to distinguish true protein-coding sequences from spurious ones. First, a TIdeS model was trained based on the *Schmidingerella* reference transcriptome and a set of reference protein sequences from six diverse eukaryotes prepared with “prep_tides_db.sh”. Next, the model was used to infer full-length ORFs in transcript sequences of each assembled transcriptome with default parameters and the ciliate genetic code (-g 6) for the correct translation of codons.

### Proteomics of *Schmidingerella* shells

#### Preparation of shells

Empty and intact shells of *Schmidingerella* specimens (SPMC 176) were continuously collected from the culture material, thoroughly washed in ddH_2_O to remove salt, and air-dried in 1.5 ml Eppendorf tubes, using a desiccator (Supplementary Method 4). Prior to enzymatic digestion, air-dried shells were resuspended in 100 µl molecular grade water and transferred to glass depression slides. Next, shells were washed two or three times, by transferring them through drops of 100 µl molecular grade water on clean depression slides with a finely drawn glass Pasteur pipette. Subsequently, shells were transferred in a minimum volume of water to a glass depression slide with 100 µl of 50 mM TEAB buffer (triethyl-ammonium bicarbonate, pH 8.5, Sigma-Aldrich). Finally, 100 µl of TEAB buffer with the shells were pipetted into a 1.5 ml LoBind® Eppendorf tube. Three tubes with about 1700 shells in total were prepared for the enzymatic digestion experiments.

#### Enzymatic digestion

Enzymatic digestion experiments were conducted with three proteases (proteinase K, trypsin, and elastase). Samples were incubated at optimal digestion temperatures according to manufacturer’s specifications in an Eppendorf Thermomixer C at 1000 rpm until most shells visibly dissolved in the solution. After digestion, samples were stored at –20 °C until processing for mass spectrometry. In the first digestion experiment, two samples were prepared, using proteinase K (Qiagen Proteinase K solution) and sequencing grade modified trypsin (Promega). The first sample (Sample 1) containing about 690 shells in 100 µl TEAB buffer was incubated with 3 µl proteinase K (0.3 mg/ml) at 57 °C for 7 h and subsequently with 1 µl trypsin (1 mg/ml) at 37 °C overnight. The second sample (Sample 2) containing about 490 shells in 100 µl TEAB buffer was incubated solely with 2 µl proteinase K (0.3 mg/ml) at 57 °C for 4 h. In the second digestion experiment (Sample 3), about 500 shells were incubated with 2 µl elastase (1 mg/ml; Promega) in 100 µl TEAB buffer at 37 °C for 18 h. Control samples containing identical amounts of TEAB buffer and proteases without shells were incubated alongside the shell digestion samples.

#### Tandem-mass spectrometry

The shell protein digests (50 µl of 100 µl) were acidified, using 2.50% (v/v) aqueous trifluoroacetic acid (Sigma-Aldrich) to a final concentration of 0.20% (v/v). Subsequently, the digests were purified, using Pierce™ C18 Tips (Thermo Fisher Scientific) according to the manufacturer’s protocol. The purified peptides were dried at 45 °C and 850 rpm in a Savant SpeedVac™ SPD210 Vacuum Concentrator (Thermo Fisher Scientific) equipped with a VLP120 vacuum pump and a RVT5105 refrigerated vapour trap (Thermo Fisher Scientific). The dried samples were resuspended in 20 µl of 1% (v/v) acetonitrile (ACN; Thermo Fisher Scientific) and 0.1% (v/v) formic acid (FA; ≥98%; Sigma-Aldrich) in water, which corresponds to the starting conditions in the ultra-high-performance liquid chromatography (UHPLC) method.

To elucidate their respective sequences, the peptides generated by the various enzymatic digestions of the *Schmidingerella* shells (Samples 1–3) were separated employing reversed-phase UHPLC on a C18-based nano-HPLC column and subsequently analysed by tandem-mass spectrometry, employing a hybrid quadrupole-orbitrap mass spectrometer (Thermo Fisher Scientific) in data-dependent acquisition mode. Chromatographic separation of 2 µl sample was carried out, employing reversed phase HPLC on a Vanquish Neo™ UHPLC system (Thermo Fisher Scientific), using an Aurora Ultimate TS C18 column (250 × 0.075 mm i.d.; dp 1.7 µm) from IonOpticks. For the separation, 0.1% aqueous formic acid (solvent A) and 0.1% formic acid in acetonitrile (solvent B) were pumped at a flow rate of 300 nl per minute in the following order: 1% B for 5 min, a linear gradient from 1 to 5% B in 5 min, a second linear gradient from 5 to 25% B in 75 min, and a third linear gradient from 25 to 35% B in 5 min. This was followed by flushing at 80% B for 5 min and column re-equilibration at 1% B for 15 min. The column temperature was kept constant at 50 °C, the autosampler was kept at 7 °C. The UHPLC system was hyphenated to a Q Exactive™ Hybrid Quadrupole-Orbitrap™ mass spectrometer via a Nanospray Flex™ ion source (both from Thermo Fisher Scientific). The spray voltage was set to 1.5 kV, S-lens RF level to 60.0, and capillary temperature to 250 °C. To increase tandem-mass spectra sampling depth and maximise the coverage of possible precursor ions and subsequently peptides, each sample was analysed in triplicate. First, each scan cycle consisted of a full scan at a scan range of m/z 350–1200 at a resolution setting of 70,000 at m/z 200, followed by 15 data-dependent higher-energy collisional dissociation (HCD) scans, using an isolation window of 1.0 m/z at 29% normalized collision energy at a resolution setting of 17,500 at m/z 200. Second, each scan cycle consisted of a full scan at a scan range of m/z 600–1800 at a resolution setting of 70,000 at m/z 200, followed by 15 data-dependent higher-energy collisional dissociation scans, using an isolation window of 1.0 m/z at 25% normalized collision energy at a resolution setting of 17,500 at m/z 200. Third, each scan cycle consisted of a full scan at a scan range of m/z 1000–2200 at a resolution setting of 70,000 at m/z 200, followed by 15 data-dependent higher-energy collisional dissociation scans, using an isolation window of 1.0 m/z at 29% normalized collision energy at a resolution setting of 17,500 at m/z 200. The automatic gain control (AGC) target was set to 1e6 charges with a maximum injection time of 100 ms for the full scan and 5e5 charges and 60 ms for the HCD scans in all three methods. Already isolated precursor ions were dynamically excluded for fragmentation for 10 s. Data acquisition was conducted, using Thermo Scientific™ Chromeleon™ 7.2 CDS (Thermo Fisher Scientific).

### Identification and analyses of Tintinnidorin proteins

#### Combining mass spectrometry and transcriptome data

Raw mass spectra data files were converted to the mzML format with ThermoRawFileParser v1.4.4^80^ (https://github.com/CompOmics/ThermoRawFileParser) and subsequently processed with Casanovo v4.2.0^21^ (https://github.com/Noble-Lab/casanovo), using default weights^81^ and settings. Casanovo is a machine learning model for de novo peptide sequencing that does not rely on reference sequences. Peptide sequences identified from the mass spectra were then matched to protein sequences in the *Schmidingerella* reference proteome. Across the entire reference proteome, only three full-length protein sequences (Tintinnidorin-1-alpha, beta, and gamma) received high numbers of peptide matches and were therefore selected for subsequent analyses.

To quantify the peptide coverage of the identified protein sequences and proteases, the Casanovo output was further processed with the Stitch software v1.5.0^82^ (https://github.com/snijderlab/stitch), using Tintinnidorin-1-alpha, beta, and gamma, proteinase K (Uniprot entry: P06873), trypsin (Uniprot entry: P00761), and elastase (Uniprot entry: P00772) as templates. To assess the confidence of the de novo sequencing results, selected peptide sequences identified by Casanovo were additionally evaluated by manual inspection of tandem-mass spectra with a fragment match tolerance of 15 ppm of predicted and measured fragments, using the proteomics data viewer PDV^83^. Furthermore, a mirror plot of the tandem-mass spectra was predicted on the respective peptide sequences based on AlphaPept^84^, using settings matching the measurements for each spectrum, such as precursor charge and fragmentation energy, allowing comparison between experimental spectra and corresponding in silico predictions (Supplementary Fig. 11).

#### Cellular localization

Subcellular localization and sorting signals of Tintinnidorin proteins were predicted with DeepLoc v2.1^23^ (https://services.healthtech.dtu.dk/services/DeepLoc-2.1; accessed on 04/17/2024; option “High quality”). The transformer-based protein language model differentiated between membrane-associated and soluble proteins. Protein sequences with predicted sorting signal peptides were additionally analysed with SignalP v6.0^85^ (https://services.healthtech.dtu.dk/services/SignalP-6.0/; accessed on 04/24/2024; option “Eukarya”, only predicts Sec/SPI “standard” secretory signal peptides).

#### Transcript level expression

Transcript abundances of Tintinnidorin-1-alpha, beta, and gamma for each *Schmidingerella* cell were quantified by transcripts per million (TPM) values estimated with Salmon v1.10.0^86^. TPM values were determined from filtered and quality trimmed paired-end reads (FPE and RPE) with the most conservative settings (--validateMappings, --gcBias, --seqBias, --posBias, --mimicStrictBT2) to remove reads with indels and to account for compositional and coverage biases. Transcript level expression values per cell were extracted from Salmon’s “quant.sf” files with tximport^87^ in RStudio (2023.12.1 + 402)^88^ and plotted with pheatmap v1.0.12 (https://github.com/raivokolde/pheatmap).

#### Orthogroup inference and homology search

Orthogroup inference of tintinnid ciliate protein-coding genes (20 *Schmidingerella* cells, the *Schmidingerella* reference transcriptome, and 46 single-cell transcriptomes of other tintinnid ciliates) included 232 genomes and transcriptomes from a wide variety of bacteria, archaea, and eukary-otes (Supplementary Data 5). Coding sequence files (CDS) of genomes were translated to amino acid sequences with the appropriate genetic code, using the third script of the EukPhylo v1.0 pipeline (https://zenodo.org/records/13323372). Open reading frames of protein-coding sequences in transcriptomes mainly comprising ciliates were determined with TIdeS, using the model based on the *Schmidingerella* reference transcriptome. Partial Tintinnidorin sequences that did not comprise the entire ORF were identified by sequence similarity searches against full-length Tintinnidorin sequences (CD-HIT and BLAST) and manually aligned to determine the correct reading frame. Subsequently, partial sequences were translated to amino acid sequences and added to the respective single-cell transcriptome assemblies. Orthogroups and hierarchical orthogroups were identified with OrthoFinder v2.5.5, using default settings^89^ (https://github.com/davidemms/OrthoFinder). In short, OrthoFinder performs an all-versus-all DIAMOND search to cluster genes into orthogroups, from which gene trees are computed. Next, gene trees are concatenated to infer a rooted species tree. Finally, hierarchical orthogroups are defined by reconciling gene trees with the species tree, identifying the descendant genes of each ancestral gene at successive internal nodes. Additional homology searches across a curated dataset of 1000 diverse species of bacteria, archaea, and eukaryotes were conducted with EukPhylo v1.0 (Supplementary Method 6).

#### Public sequence repository search

Two public repositories were screened for homologues to Tintinnidorin sequences. The Ocean Gene Atlas^25,90^ (https://tara-oceans.mio.osupytheas.fr/ocean-gene-atlas/) was searched, using Tintinnidorin nucleotide sequences as queries against the MATOUv1+T dataset^19^ with default settings. This dataset contains 116 million unigenes that were obtained from the Tara Oceans expedition^91^ and assembled with reads from metatranscriptomes clustered at 95% identity. Potential sequence homologues were downloaded from the web service for further processing (last accessed on 05/12/2025). Additional sequences were acquired from the North Pacific Eukaryotic Gene Catalogue (NPEGC)^20^ repository v0.92 (https://zenodo.org/records/13826820). Potential sequence homologues from both datasets were processed in AliView v1.28^92^ to determine the correct open reading frame, using Tintinnidorin-1-alpha, beta, and gamma sequences as reference. Only sequences containing the complete reading frame (*n* = 72), i.e., from start to stop codon, were retained for global distribution mapping and phylogenetic analyses. The locations of metatranscriptome samples, in which reads of full-length Tintinnidorin sequences were detected (Supplementary Data 14), and literature records of tintinnid ciliate occurrences (according to ref. ^57^, pers. commun.) were plotted on a world map with Matplotlib v3.10^93^ and the package Cartopy v0.24.1 (10.5281/zenodo.1182735).

#### Phylogenetic analyses

Full-length Tintinnidorin sequences detected in the two public repositories (*n* = 72) plus Tintinnidorin-1-alpha, beta, gamma (*Schmidingerella*) and Tintinnidorin-2 (*Tintinnopsis cylindrica*) sequences from the single-cell transcriptomes (*n* = 6) were aligned with MAFFT v7.526^94^, using the Smith-Waterman algorithm (--localpair) with 1000 iterations (--maxiterate 1000). Subsequently, partial sequences (*n* = 12) from further single-cell transcriptomes (*Favella*, *Helicostomella*, other *Schmidingerella*, *Stenosemella*, and *Tintinnopsis* species) were added to the alignment of full-length sequences, using the “--addfragments” command. Maximum likelihood tree inference was performed in IQ-TREE v3.0.1^95^ under the variable time substitution model^96^, a proportion of invariable sites, and a FreeRate model with five categories for rate heterogeneity (-m VT + F + I + R5). Branch support was assessed, using ultrafast bootstraps (-b 1000) and SH-like approximate likelihood ratio test (-alrt 1000) with 1000 replicates. The tree was plotted and graphically edited with TreeViewer v2.2.0^97^.

### Tintinnidorin protein characteristics and structure predictions

#### Sequence architecture and amino acid composition

The structuring of Tintinnidorin protein sequences into module and linker regions was based on manual assessment of multiple sequence alignments. In the alignments, module regions consistently formed sequence blocks of 78 amino acids, mostly starting and ending with pairs of alanine and serine (sometimes alanine and aspartic acid, or rarely other residues). Regions between the modules were subsequently defined as linker regions. Amino acid compositions were calculated as fractions based on the overall protein sequence and separately on the module and linker regions, respectively. Significant differences of mean fractions and variances were tested with the Wilcoxon signed-rank test as implemented in R and the Levene’s test (10.32614/CRAN.package.car), respectively. Amino acid conservation for each position in the modules was determined by assessing the consensus annotation of the aligned full-length Tintinnidorin protein sequences in Jalview v2.11.5.0^98^.

#### Structure prediction with AlphaFold2

A local installation of AlphaFold2^42^ was used to predict the three-dimensional structures of full-length Tintinnidorin sequences detected in the two public repositories (*n* = 72) plus Tintinnidorin-1-alpha, beta, gamma (*Schmidingerella*), and Tintinnidorin-2 (*Tintinnopsis cylindrica*) sequences from the single-cell transcriptomes (*n* = 6). From the five models generated by AlphaFold2 for each sequence, respective top-ranked models were used for subsequent analyses.

#### Prediction of protein features

Secondary structure elements in the AlphaFold2 models were determined by STRIDE^43^. Disordered protein regions were predicted with metapredict V2^44^. Propensities for condensate formation were predicted with PICNIC^99^. Molecular masses and isoelectric points were calculated with ProtParam^100^. Schematic views of Tintinnidorin protein features were generated with the pgfmolbio package (http://www.ctan.org/pkg/pgfmolbio; accessed on 11/20/2025) and TeXshade^101^.

#### Structure similarity search

Since protein structure is generally more conserved than protein sequence^102^, structure-based searches were performed in addition to the sequence-based searches for identifying proteins with structures similar to the 78 Tintinnidorin models (queries). We used Foldseek^103^ easy-search (version 427df8a6b5d0ef78bee0f98cd3e6faaca18f172d; --exhaustive-search 1) to search against all experimentally determined structures available from the Protein Data Bank (PDB; https://RCSB.org)^104^ as of 11/05/2025 (1,051,375 protein chain targets) and against AlphaFold2 models for a representative set of all canonical sequences in the UniProt database^105^ as of 07/17/2024 (53,665,860 protein chain targets). Furthermore, we used TopMatch^106^ for structure searches against the abovementioned set of experimentally determined structures and against all AlphaFold2 models for proteins in the UniProt/Swiss-Prot database as of 10/19/2022 (542,378 protein chain targets). TopMatch was also used to re-align the top-ranked hits obtained by Foldseek to obtain consistent structure similarity scores across all structure searches. The extent of structure similarity was then quantified by TopMatch’s structure similarity score^106^. Model analysis and molecular graphics were performed with UCSF ChimeraX^107^.

### Reporting summary

Further information on research design is available in the Nature Portfolio Reporting Summary linked to this article.

## Supplementary information

Supplementary Information

Description of Additional Supplementary Files

Supplementary Movie 1

Supplementary Data 1-14

Supplementary Data 15

Supplementary Data 16

Reporting Summary

Transparent Peer Review file

## Source data

Source Data 1

Source Data 2

## Data Availability

The transcriptomic reads of the monoclonal *Schmidingerella* (SPMC176) cells analysed during the current study are available in BioProject PRJNA1363744. Additional single-cell transcriptomes of tintinnid ciliates used during this study are available in BioProject PRJNA1026950^18^. Consensus sequences of the 18S, 5.8S, 28S rRNA genes and the internal transcribed spacer regions (ITS1 and ITS2) of the same *Schmidingerella* specimens are available in NCBI GenBank under the accession numbers: PX559938 (18S SSU rRNA), PX559939 (ITS1-5.8S-ITS2), and PX644820 (28S LSU rRNA). The mass spectrometry proteomics data of *Schmidingerella* shells generated during the current study are available via the PRIDE partner repository [C]^108^ with the dataset identifiers PXD070957 and 10.6019/PXD070957. Supplementary information: Supplementary Notes 1–7, Supplementary Methods 1–6, Supplementary Figs. 1–11; Supplementary Files: Supplementary Movie 1, Supplementary Data 1–14 (separate sheets in one excel file), Supplementary Data 15 (fasta sequences of the 78 full-length Tintinnidorin proteins), and Supplementary Data 16 (AlphaFold2 structure models in CIF format). All supplementary files are available on Zenodo (10.5281/zenodo.20628083). Unless otherwise stated, all data supporting the results of this study can be found in the article, supplementary, and source data files provided with this paper.

## References

[1] Miserez, A., Yu, J. & Mohammadi, P. Protein-based biological materials: Molecular design and artificial production. *Chem. Rev.* **123**, 2049–2111 (2023). 36692900 10.1021/acs.chemrev.2c00621 PMC9999432

[2] Vollrath, F. Biology of spider silk. *Int. J. Biol. Macromol.* **24**, 81–88 (1999). 10342751 10.1016/s0141-8130(98)00076-2

[3] Numata, K. & Kaplan, D. L. Silk proteins: Designs from nature with multipurpose utility and infinite future possibilities. *Adv. Mater.* **37**, 2411256 (2025). 10.1002/adma.202411256 39468893

[4] Shire, E., Coimbra, A. A. B., Barba Ostria, C., Rios-Solis, L. & López Barreiro, D. Molecular design of protein-based materials – state of the art, opportunities and challenges at the interface between materials engineering and synthetic biology. *Mol. Syst. Des. Eng.* **9**, 1187–1209 (2024).

[5] Schiller, T. & Scheibel, T. Bioinspired and biomimetic protein-based fibers and their applications. *Commun. Mater.* **5**, 56 (2024).

[6] Hofman, A. H., van Hees, I. A., Yang, J. & Kamperman, M. Bioinspired underwater adhesives by using the supramolecular toolbox. *Adv. Mater.* **30**, 1704640 (2018). 10.1002/adma.201704640 29356146

[7] Raheem, A. et al. Smart biomaterials in healthcare: Breakthroughs in tissue engineering, immunomodulation, patient-specific therapies, and biosensor applications. *Appl. Phys. Rev.* **12**, 011333 (2025).

[8] Samir, A., Ashour, F. H., Hakim, A. A. A. & Bassyouni, M. Recent advances in biodegradable polymers for sustainable applications. *npj Mater. Degrad.* **6**, 68 (2022).

[9] Williamson, K. et al. A robustly rooted tree of eukaryotes reveals their excavate ancestry. *Nature* **640**, 974–981 (2025). 40074902 10.1038/s41586-025-08709-5

[10] Fernandes, N. M. & Schrago, C. G. A multigene timescale and diversification dynamics of Ciliophora evolution. *Mol. Phylogenet. Evol.* **139**, 106521 (2019). 31152779 10.1016/j.ympev.2019.106521

[11] Kofoid, C. A. & Campbell, A. S. A conspectus of the marine and fresh-water Ciliata belonging to the suborder Tintinnoinea, with descriptions of new species principally from the Agassiz Expedition to the eastern tropical Pacific 1904–1905. *Univ. Calif. Publs Zool.* **34**, 1–403 (1929).

[12] Santoferrara, L. F. & McManus, G. B. in *Zooplankton Ecology* (eds Teodósio, M. A. & Barbosa, A. B.) 85–118 (CRC Press, 2020).

[13] Agatha, S. & Bartel, H. A comparative ultrastructural study of tintinnid loricae (Alveolata, Ciliophora, Spirotricha) and a hypothesis on their evolution. *J. Eukaryot. Microbiol.* **69**, e12877 (2022). 34850491 10.1111/jeu.12877

[14] Agatha, S. & Simon, P. On the nature of tintinnid loricae (Ciliophora: Spirotricha: Tintinnina): A histochemical, enzymatic, EDX, and high-resolution TEM study. *Acta Protozool.* **51**, 1–19 (2012). 22988335 PMC3442249

[15] Entz, G., Jr. Studien über Organisation und Biologie der Tintinniden. *Arch. Protistenk.* **15**, 93–226. + Plates VIII–XXI (1909).

[16] Ganser, M. H., Weißenbacher, B. & Agatha, S. How single cells form shells: Maturation and secretion of lorica-forming material in the tintinnid *Schmidingerella* (Alveolata, Ciliophora). *J. Eukaryot. Microbiol.* **72**, e70025 (2025). 40625050 10.1111/jeu.70025 PMC12235346

[17] Laval-Peuto, M. Construction of the lorica in Ciliata Tintinnina. In vivo study of *Favella ehrenbergii*: Variability of the phenotypes during the cycle, biology, statistics, biometry. *Protistologica* **17**, 249–272 (1981).

[18] Shazib, S. U. A. et al. Phylogenomic workflow for uncultivable microbial eukaryotes using single-cell RNA sequencing − a case study with planktonic ciliates (Ciliophora, Oligotrichea). *Mol. Phylogenet. Evol.* **204**, 108239 (2025). 39551225 10.1016/j.ympev.2024.108239

[19] Carradec, Q. et al. A global ocean atlas of eukaryotic genes. *Nat. Commun.* **9**, 373 (2018). 29371626 10.1038/s41467-017-02342-1 PMC5785536

[20] Groussman, R. D., Coesel, S. N., Durham, B. P., Schatz, M. J. & Armbrust, E. V. The North Pacific Eukaryotic Gene Catalog of metatranscriptome assemblies and annotations. *Sci. Data* **11**, 1161 (2024). 39438508 10.1038/s41597-024-04005-5 PMC11496615

[21] Yilmaz, M. et al. Sequence-to-sequence translation from mass spectra to peptides with a transformer model. *Nat. Commun.* **15**, 6427 (2024). 39080256 10.1038/s41467-024-49731-x PMC11289372

[22] Agatha, S., Weißenbacher, B., Böll, L. & Ganser, M. H. Morphologic changes in the model tintinnid *Schmidingerella* (Alveolata, Ciliophora) during the cell cycle, including the first volumetric analyses of the lorica-forming material. *BMC Microbiol*. **25**, 88 (2025). 40000957 10.1186/s12866-025-03780-4 PMC11853588

[23] Ødum, M. T. et al. DeepLoc 2.1: Multi-label membrane protein type prediction using protein language models. *Nucl. Acids Res.* **52**, W215–W220 (2024). 38587188 10.1093/nar/gkae237 PMC11223819

[24] Katz, L. A., Leleu, M., Ani, G., Gawron, R. & Cote-L’Heureux, A. Rethinking large-scale phylogenomics with EukPhylo v.1.0, a flexible toolkit to enable phylogeny-informed data curation and analyses of diverse eukaryotic lineages. *mBio*. **16**, e01770-25 (2025). 40862604 10.1128/mbio.01770-25 PMC12506077

[25] Vernette, C. et al. The Ocean Gene Atlas v2.0: Online exploration of the biogeography and phylogeny of plankton genes. *Nucl. Acids Res.* **50**, W516–W526 (2022). 35687095 10.1093/nar/gkac420 PMC9252727

[26] Landreh, M. et al. Liquid-liquid crystalline phase separation of spider silk proteins. *Commun. Chem.* **7**, 260 (2024). 39533043 10.1038/s42004-024-01357-2 PMC11557605

[27] Stewart, R. J., Wang, C. S., Song, I. T. & Jones, J. P. The role of coacervation and phase transitions in the sandcastle worm adhesive system. *Adv. Colloid Interface Sci.* **239**, 88–96 (2017). 27393642 10.1016/j.cis.2016.06.008 PMC5182194

[28] Craig, H. C. et al. Posttranslational modifications in spider silk influence conformation and dimerization dynamics. *MRS Bull.* **49**, 1192–1204 (2024).

[29] Priemel, T., Degtyar, E., Dean, M. N. & Harrington, M. J. Rapid self-assembly of complex biomolecular architectures during mussel byssus biofabrication. *Nat. Commun.* **8**, 14539 (2017). 28262668 10.1038/ncomms14539 PMC5343498

[30] The UniProt Consortium. UniProt: The Universal Protein Knowledgebase in 2025. *Nucl. Acids Res.* **53**, D609–D617 (2025). 39552041 10.1093/nar/gkae1010 PMC11701636

[31] Maraldo, A. et al. Biochemical methods for producing and characterising recombinant spider silks. *Front. Arachn. Sci.* **3**, 1488680 (2025).

[32] Feughelman, M. Natural protein fibers. *J. Appl. Polym. Sci.* **83**, 489–507 (2002).

[33] Petrone, L. et al. Mussel adhesion is dictated by time-regulated secretion and molecular conformation of mussel adhesive proteins. *Nat. Commun.* **6**, 8737 (2015). 26508080 10.1038/ncomms9737 PMC4640085

[34] Barik, S. The uniqueness of tryptophan in biology: Properties, metabolism, interactions and localization in proteins. *Int. J. Mol. Sci.* **21**, 8776 (2020). 33233627 10.3390/ijms21228776 PMC7699789

[35] Partlow, B. P., Bagheri, M., Harden, J. L. & Kaplan, D. L. Tyrosine templating in the self-assembly and crystallization of silk fibroin. *Biomacromolecules* **17**, 3570–3579 (2016). 27736062 10.1021/acs.biomac.6b01086

[36] Warrender, A. K., Pan, J., Pudney, C., Arcus, V. L. & Kelton, W. Red edge excitation shift spectroscopy is highly sensitive to tryptophan composition. *J. R. Soc. Interface* **20**, 20230337 (2023). 37935360 10.1098/rsif.2023.0337 PMC10645072

[37] Ahsan, R., Blanche, W. & Katz, L. A. Macronuclear development in ciliates, with a focus on nuclear architecture. *J. Eukaryot. Microbiol.* **69**, e12898 (2022). 35178799 10.1111/jeu.12898 PMC9391682

[38] Katz, L. A. & Kovner, A. M. Alternative processing of scrambled genes generates protein diversity in the ciliate *Chilodonella uncinata*. *J. Exp. Zool. (Mol. Dev. Evol.)* **314B**, 480–488 (2010). 10.1002/jez.b.21354 PMC2933047 20700892

[39] Gao, F., Roy, S. W. & Katz, L. A. Analyses of alternatively processed genes in ciliates provide insights into the origins of scrambled genomes and may provide a mechanism for speciation. *mBio* **6**, e01998–14 (2015). 25650397 10.1128/mBio.01998-14 PMC4324306

[40] Zufall, R. A., McGrath, C. L., Muse, S. V. & Katz, L. A. Genome architecture drives protein evolution in ciliates. *Mol. Biol. Evol.* **23**, 1681–1687 (2006). 16760419 10.1093/molbev/msl032

[41] Yan, Y., Maurer-Alcalá, X. X., Knight, R., Pond, S. L. K. & Katz, L. A. Single-cell transcriptomics reveal a correlation between genome architecture and gene family evolution in ciliates. *mBio* **10**, e02524–19 (2019). 31874915 10.1128/mBio.02524-19 PMC6935857

[42] Jumper, J. et al. Highly accurate protein structure prediction with AlphaFold. *Nature* **596**, 583–589 (2021). 34265844 10.1038/s41586-021-03819-2 PMC8371605

[43] Frishman, D. & Argos, P. Knowledge-based protein secondary structure assignment. *Proteins: Struct., Funct., Bioinf.* **23**, 566–579 (1995). 10.1002/prot.340230412 8749853

[44] Emenecker, R. J., Griffith, D. & Holehouse, A. S. Metapredict: A fast, accurate, and easy-to-use predictor of consensus disorder and structure. *Biophys. J.* **120**, 4312–4319 (2021). 10.1016/j.bpj.2021.08.039 PMC8553642 34480923

[45] Lay, M. G., Oktaviani, N. A., Malay, A. D. & Numata, K. Exploring the self-assembly of silk proteins through liquid-liquid phase separation. *Polym. J.* **57**, 799–814 (2025).

[46] Li, T., Ilhamsyah, D., Tai, B. & Shen, Y. Functional biomaterials derived from protein liquid–liquid phase separation and liquid-to-solid transition. *Adv. Mater.* **37**, 2414703 (2025). 39924792 10.1002/adma.202414703 PMC12138867

[47] Harmon, T. S., Holehouse, A. S., Rosen, M. K. & Pappu, R. V. Intrinsically disordered linkers determine the interplay between phase separation and gelation in multivalent proteins. *eLife* **6**, e30294 (2017). 29091028 10.7554/eLife.30294 PMC5703641

[48] Ankle, M. R. & Joshi, P. S. A study to evaluate the efficacy of xylene-free hematoxylin and eosin staining procedure as compared to the conventional hematoxylin and eosin staining: an experimental study. *J. Oral. Maxillofac. Pathol.* **15**, 161–167 (2011). 22529574 10.4103/0973-029X.84482 PMC3329695

[49] Maraldo, A., Rnjak-Kovacina, J. & Marquis, C. Tyrosine – a structural glue for hierarchical protein assembly. *Trends Biochem. Sci.* **49**, 633–648 (2024). 38653686 10.1016/j.tibs.2024.03.014

[50] Iadanza, M. G., Jackson, M. P., Hewitt, E. W., Ranson, N. A. & Radford, S. E. A new era for understanding amyloid structures and disease. *Nat. Rev. Mol. Cell Biol.* **19**, 755–773 (2018). 30237470 10.1038/s41580-018-0060-8 PMC7617691

[51] Keten, S., Xu, Z., Ihle, B. & Buehler, M. J. Nanoconfinement controls stiffness, strength and mechanical toughness of β-sheet crystals in silk. *Nat. Mater.* **9**, 359–367 (2010). 20228820 10.1038/nmat2704

[52] Chen, P. et al. pH-triggered transition of silk fibroin from spherical micelles to nanofibrils in water. *Macromol. Res.* **16**, 539–543 (2008).

[53] Moreno-Tortolero, R. O. et al. Molecular organization of fibroin heavy chain and mechanism of fibre formation in *Bombyx mori*. *Commun. Biol.* **7**, 786 (2024). 10.1038/s42003-024-06474-1 PMC11217467 38951579

[54] Martinez Rodriguez, N. R., Das, S., Kaufman, Y., Israelachvili, J. N. & Waite, J. H. Interfacial pH during mussel adhesive plaque formation. *Biofouling* **31**, 221–227 (2015). 25875963 10.1080/08927014.2015.1026337 PMC4420479

[55] Zaragoza-Solas, A. & Baltar, F. Ayu: A machine intelligence tool for identification of extracellular proteins in the marine secretome. *Nat. Commun.* **16**, 2793 (2025). 40118827 10.1038/s41467-025-57974-5 PMC11928666

[56] Makwana, K. M. & Mahalakshmi, R. Implications of aromatic–aromatic interactions: from protein structures to peptide models. *Protein Sci.* **24**, 1920–1933 (2015). 26402741 10.1002/pro.2814 PMC4815235

[57] Dolan, J. R. & Pierce, R. W. in *The Biology and Ecology of Tintinnid Ciliates: Models for Marine Plankton* (eds Dolan, J. R. et al.) 214–243 (John Wiley & Sons, Ltd, 2013).

[58] Santoferrara, L. F., Rubin, E. & McManus, G. B. Global and local DNA (meta)barcoding reveal new biogeography patterns in tintinnid ciliates. *J. Plankton Res.* **40**, 209–221 (2018).

[59] Pesant, S. et al. Open science resources for the discovery and analysis of Tara Oceans data. *Sci. Data* **2**, 150023 (2015). 26029378 10.1038/sdata.2015.23 PMC4443879

[60] Hölzer, M. & Marz, M. De novo transcriptome assembly: a comprehensive cross-species comparison of short-read RNA-Seq assemblers. *GigaScience* **8**, 1–16 (2019). 10.1093/gigascience/giz039 PMC6511074 31077315

[61] McManus, G. B. & Santoferrara, L. F. in *The Biology and Ecology of Tintinnid Ciliates: Models for Marine Plankton* (eds Dolan, J. R. et al.) 198–213 (John Wiley & Sons, Ltd, 2013).

[62] Stoecker, D. K. in *The Biology and Ecology of Tintinnid Ciliates: Models for Marine Plankton* (eds Dolan, J. R. et al.) 122–144 (John Wiley & Sons, Ltd, 2013).

[63] Price, A. M. & Pospelova, V. High-resolution sediment trap study of organic-walled dinoflagellate cyst production and biogenic silica flux in Saanich Inlet (BC, Canada). *Mar. Micropaleontol.* **80**, 18–43 (2011).

[64] Boltovskoy, D., Alder, V. A. & Abelmann, A. Annual flux of Radiolaria and other shelled plankters in the eastern equatorial Atlantic at 853 m: Seasonal variations and polycystine species-specific responses. *Deep-Sea Res. I* **40**, 1863–1895 (1993).

[65] Suzuki, T. & Taniguchi, A. Sinking rate of loricae of some common tintinnid ciliates. *Fish. Oceanogr.* **4**, 257–263 (1995).

[66] Numata, K. The biology of natural polymers accelerates and expands the science of biomacromolecules: A focus on structural proteins. *Biomacromolecules* **26**, 1393–1403 (2025). 39965779 10.1021/acs.biomac.4c01621 PMC11898061

[67] Hu, W. et al. A molecular atlas reveals the tri-sectional spinning mechanism of spider dragline silk. *Nat. Commun.* **14**, 837 (2023). 36792670 10.1038/s41467-023-36545-6 PMC9932165

[68] Babb, P. L. et al. The *Nephila clavipes* genome highlights the diversity of spider silk genes and their complex expression. *Nat. Genet.* **49**, 895–903 (2017). 28459453 10.1038/ng.3852

[69] Ma, Y. et al. A single-cell transcriptomic atlas characterizes the silk-producing organ in the silkworm. *Nat. Commun.* **13**, 3316 (2022). 35680954 10.1038/s41467-022-31003-1 PMC9184679

[70] Kono, N. et al. Multicomponent nature underlies the extraordinary mechanical properties of spider dragline silk. *Proc. Nat. Acad. Sci.* **118**, e2107065118 (2021). 34312234 10.1073/pnas.2107065118 PMC8346794

[71] Guillard, R. R. L. in *Culture of Marine Invertebrate Animals* (eds Smith, W. L. & Chanley, M. H.) 29–60 (Plenum Press, 1975).

[72] Picelli, S. et al. Full-length RNA-seq from single cells using Smart-seq2. *Nat. Protoc.* **9**, 171–181 (2014). 24385147 10.1038/nprot.2014.006

[73] FastQC: A quality control tool for high throughput sequence data. Babraham Bioinformatics (2010).

[74] Ewels, P., Magnusson, M., Lundin, S. & Käller, M. MultiQC: Summarize analysis results for multiple tools and samples in a single report. *Bioinformatics* **32**, 3047–3048 (2016). 27312411 10.1093/bioinformatics/btw354 PMC5039924

[75] Bushnell, B., Rood, J. & Singer, E. BBMerge – accurate paired shotgun read merging via overlap. *PLoS ONE* **12**, e0185056 (2017). 29073143 10.1371/journal.pone.0185056 PMC5657622

[76] Deng, Z.-L., Münch, P. C., Mreches, R. & McHardy, A. C. Rapid and accurate identification of ribosomal RNA sequences via deep learning. *Nucl. Acids Res.* **50**, e60 (2022). 35188571 10.1093/nar/gkac112 PMC9177968

[77] Bushmanova, E., Antipov, D., Lapidus, A. & Prjibelski, A. D. rnaSPAdes: A de novo transcriptome assembler and its application to RNA-Seq data. *GigaScience* **8**, 1–13 (2019). 10.1093/gigascience/giz100 PMC6736328 31494669

[78] Prjibelski, A., Antipov, D., Meleshko, D., Lapidus, A. & Korobeynikov, A. Using SPAdes de novo assembler. *Curr. Protoc. Bioinf.* **70**, e102 (2020). 10.1002/cpbi.102 32559359

[79] Maurer-Alcalá, X. X. & Kim, E. TIdeS: A comprehensive framework for accurate open reading frame identification and classification in eukaryotic transcriptomes. *Genome Biol. Evol.* **16**, evae252 (2024). 39570867 10.1093/gbe/evae252 PMC11631190

[80] Hulstaert, N. et al. ThermoRawFileParser: Modular, scalable, and cross-platform RAW file conversion. *J. Proteome Res.* **19**, 537–542 (2020). 31755270 10.1021/acs.jproteome.9b00328 PMC7116465

[81] Melendez, C. et al. Accounting for digestion enzyme bias in Casanovo. *J. Proteome Res.* **23**, 4761–4769 (2024). 39213590 10.1021/acs.jproteome.4c00422

[82] Schulte, D., Peng, W. W. & Snijder, J. Stitch (Version 1.5.0). GitHub (2024).

[83] Li, K., Vaudel, M., Zhang, B., Ren, Y. & Wen, B. PDV: An integrative proteomics data viewer. *Bioinformatics* **35**, 1249–1251 (2018). 10.1093/bioinformatics/bty770 PMC6821182 30169737

[84] Strauss, M. T. et al. AlphaPept: A modern and open framework for MS-based proteomics. *Nat. Commun.* **15**, 2168 (2024). 38461149 10.1038/s41467-024-46485-4 PMC10924963

[85] Teufel, F. et al. SignalP 6.0 predicts all five types of signal peptides using protein language models. *Nat. Biotechnol.* **40**, 1023–1025 (2022). 34980915 10.1038/s41587-021-01156-3 PMC9287161

[86] Patro, R., Duggal, G., Love, M. I., Irizarry, R. A. & Kingsford, C. Salmon provides fast and bias-aware quantification of transcript expression. *Nat. Meth.* **14**, 417–419 (2017). 10.1038/nmeth.4197 PMC5600148 28263959

[87] Soneson, C., Love, M. I. & Robinson, M. D. Differential analyses for RNA-seq: Transcript-level estimates improve gene-level inferences. *F1000Res*. **4**, 1521 (2016). 10.12688/f1000research.7563.1 PMC4712774 26925227

[88] Posit team. RStudio: Integrated Development Environment for R. Posit Software, PBC (Boston, MA, 2024).

[89] Emms, D. M. & Kelly, S. OrthoFinder: Phylogenetic orthology inference for comparative genomics. *Genome Biol.* **20**, 238 (2019). 31727128 10.1186/s13059-019-1832-y PMC6857279

[90] Villar, E. et al. The Ocean Gene Atlas: Exploring the biogeography of plankton genes online. *Nucl. Acids Res.* **46**, W289–W295 (2018). 29788376 10.1093/nar/gky376 PMC6030836

[91] Bork, P. et al. Tara Oceans studies plankton at planetary scale. *Science* **348**, 873 (2015). 25999501 10.1126/science.aac5605

[92] Larsson, A. AliView: A fast and lightweight alignment viewer and editor for large datasets. *Bioinformatics* **30**, 3276–3278 (2014). 25095880 10.1093/bioinformatics/btu531 PMC4221126

[93] Hunter, J. D. Matplotlib: A 2D graphics environment. *Comput. Sci. Eng.* **9**, 90–95 (2007).

[94] Katoh, K., Kuma, K., Toh, H. & Miyata, T. MAFFT version 5: Improvement in accuracy of multiple sequence alignment. *Nucl. Acids Res.* **33**, 511–518 (2005). 15661851 10.1093/nar/gki198 PMC548345

[95] Wong, T. K. F. et al. IQ-TREE 3: Phylogenomic inference software using complex evolutionary models. *Mol. Biol. Evol.* **43**, 1–6 (2026). 10.1093/molbev/msag117 PMC13191116 42085559

[96] Müller, T. & Vingron, M. Modeling amino acid replacement. *J. Comput. Biol.* **7**, 761–776 (2000). 11382360 10.1089/10665270050514918

[97] Bianchini, G. & Sánchez-Baracaldo, P. TreeViewer: Flexible, modular software to visualise and manipulate phylogenetic trees. *Ecol. Evol.* **14**, e10873 (2024). 38314311 10.1002/ece3.10873 PMC10834882

[98] Waterhouse, A. M., Procter, J. B., Martin, D. M. A., Clamp, M. & Barton, G. J. Jalview Version 2 - a multiple sequence alignment editor and analysis workbench. *Bioinformatics* **25**, 1189–1191 (2009). 19151095 10.1093/bioinformatics/btp033 PMC2672624

[99] Hadarovich, A. et al. PICNIC accurately predicts condensate-forming proteins regardless of their structural disorder across organisms. *Nat. Commun.* **15**, 10668 (2024). 39663388 10.1038/s41467-024-55089-x PMC11634905

[100] Gasteiger, E. et al. in *The Proteomics Protocols Handbook* (ed Walker, J. M.) 571–607 (Humana Press, 2005).

[101] Beitz, E. TeXshade: Shading and labeling of multiple sequence alignments using LaTeX2e. *Bioinformatics* **16**, 135–139 (2000). 10842735 10.1093/bioinformatics/16.2.135

[102] Chothia, C. & Lesk, A. M. The relation between the divergence of sequence and structure in proteins. *EMBO J.* **5**, 823–826 (1986). 3709526 10.1002/j.1460-2075.1986.tb04288.x PMC1166865

[103] van Kempen, M. et al. Fast and accurate protein structure search with Foldseek. *Nat. Biotechnol.* **42**, 243–246 (2024). 37156916 10.1038/s41587-023-01773-0 PMC10869269

[104] Berman, H. M. et al. The Protein Data Bank. *Nucl. Acids Res.* **28**, 235–242 (2000). 10592235 10.1093/nar/28.1.235 PMC102472

[105] Varadi, M. et al. AlphaFold Protein Structure Database in 2024: Providing structure coverage for over 214 million protein sequences. *Nucl. Acids Res.* **52**, D368–D375 (2023). 10.1093/nar/gkad1011 PMC10767828 37933859

[106] Wiederstein, M. & Sippl, M. J. TopMatch-web: Pairwise matching of large assemblies of protein and nucleic acid chains in 3D. *Nucl. Acids Res.* **48**, W31–W35 (2020). 32479639 10.1093/nar/gkaa366 PMC7319569

[107] Pettersen, E. F. et al. UCSF ChimeraX: Structure visualization for researchers, educators, and developers. *Protein Sci.* **30**, 70–82 (2021). 32881101 10.1002/pro.3943 PMC7737788

[108] Perez-Riverol, Y. et al. The PRIDE database at 20 years: 2025 update. *Nucl. Acids Res.* **53**, D543–D553 (2025). 39494541 10.1093/nar/gkae1011 PMC11701690

[109] Laval-Peuto, M. in *Traité de Zoologie. Anatomie, systématique, biologie. 2. Infusoires ciliés. 2. Systématique* (ed P. de Puytorac) 181–219 (Masson, 1994).

