# Supplementary material for: Self-assembling proteins compose the chemically resistant shell biomaterial of planktonic tintinnid ciliates
Source: Nat Commun. 2026 Jun 13;17:7507. doi: 10.1038/s41467-026-74402-4 (PMC13408134; doi:10.1038/s41467-026-74402-4)
Supplement: Supplementary file 1 — Supplementary Information [file 41467_2026_74402_MOESM1_ESM.pdf]

Supplementary Information

Self-assembling proteins compose the chemically resistant shell biomaterial of planktonic tintinnid ciliates

Maximilian H. Ganser[1*], Markus Wiederstein[2], Christof Regl[2], Laura A. Katz[3] & Sabine Agatha[1*]

[1]Department of Environment and Biodiversity, University of Salzburg, Salzburg, Austria
[2]Department of Biosciences, University of Salzburg, Salzburg, Austria
[3]Department of Biological Sciences, Smith College, Northampton, Massachusetts, USA

*Corresponding authors:

Maximilian H. Ganser, maximilian.ganser@plus.ac.at,

Sabine Agatha, sabine.agatha@plus.ac.at

Content

Supplementary Figures
Supplementary Figure 1: Relative gene expression of Tintinnidorin-1-alpha, beta, and gamma proteins during the cell cycle of monoclonal *Schmidingerella* specimens **3**
Supplementary Figure 2: Species tree computed by Orthofinder2 based on 298 genomes and transcriptomes **4**
Supplementary Figure 3: Structure similarities of the 78 full-length Tintinnidorin proteins..... **5**
Supplementary Figure 4: Comparisons of amino acid compositions **6**
Supplementary Figure 5: Autofluorescence of tintinnid ciliate shells excited with UV light..... **7**
Supplementary Figure 6: Sequence and structural characteristics for Tintinnidorin-1-alpha, beta, and gamma of *Schmidingerella* and Tintinnidorin-2-alpha of *Tintinnopsis cylindrica* **8**
Supplementary Figure 7: Pairwise nucleotide sequence divergence and similarity of Tintinnidorin-1-alpha, beta, and gamma **9**
Supplementary Figure 8: Structure models of Tintinnidorin-1-alpha, beta, and gamma from *Schmidingerella* and Tintinnidorin-2-alpha from *Tintinnopsis cylindrica* predicted by AlphaFold2 ..... **10**
Supplementary Figure 9: Structure models of the 72 full-length Tintinnidorin proteins extracted from the Tara Oceans database and North Pacific Eukaryotic Gene Catalog **11**
Supplementary Figure 10: Secondary structure assignments and propensities for disorder in the 78 full-length Tintinnidorin proteins **12**
Supplementary Figure 11: Mass spectra of Tintinnidorin-1 peptides identified by Casanovo and verified by secondary assignment of fragment masses in the proteomics data viewer PDV (Supplementary Data 2) **13**

Supplementary Notes
Supplementary Note 1: Function of tintinnid shells **20**
Supplementary Note 2: Properties of the shell biomaterial..... **20**
Supplementary Note 3: Reorganization of membranellar zone in tintinnids **21**
Supplementary Note 4: Nomenclature of Tintinnidorin proteins **21**
Supplementary Note 5: Supposed evolutionary mechanisms **21**
Supplementary Note 6: Storage of structural proteins in tintinnids compared to animal silk glands **22**
Supplementary Note 7: Influence of ionic composition on material assembly **22**
Supplementary Note 8: Origin of images in Figure 1 ..... **23**

Supplementary Methods
Supplementary Method 1: Species identification in *Schmidingerella* **23**
Supplementary Method 2: Cultivation of tintinnid ciliates **24**
Supplementary Method 3: Ribosomal RNA sequence assembly..... **24**
Supplementary Method 4: Isolation of tintinnid shells for proteomics **25**
Supplementary Method 5: Interpretation of proteomic data **25**
Supplementary Method 6: Homology search details..... **26**

References **27**

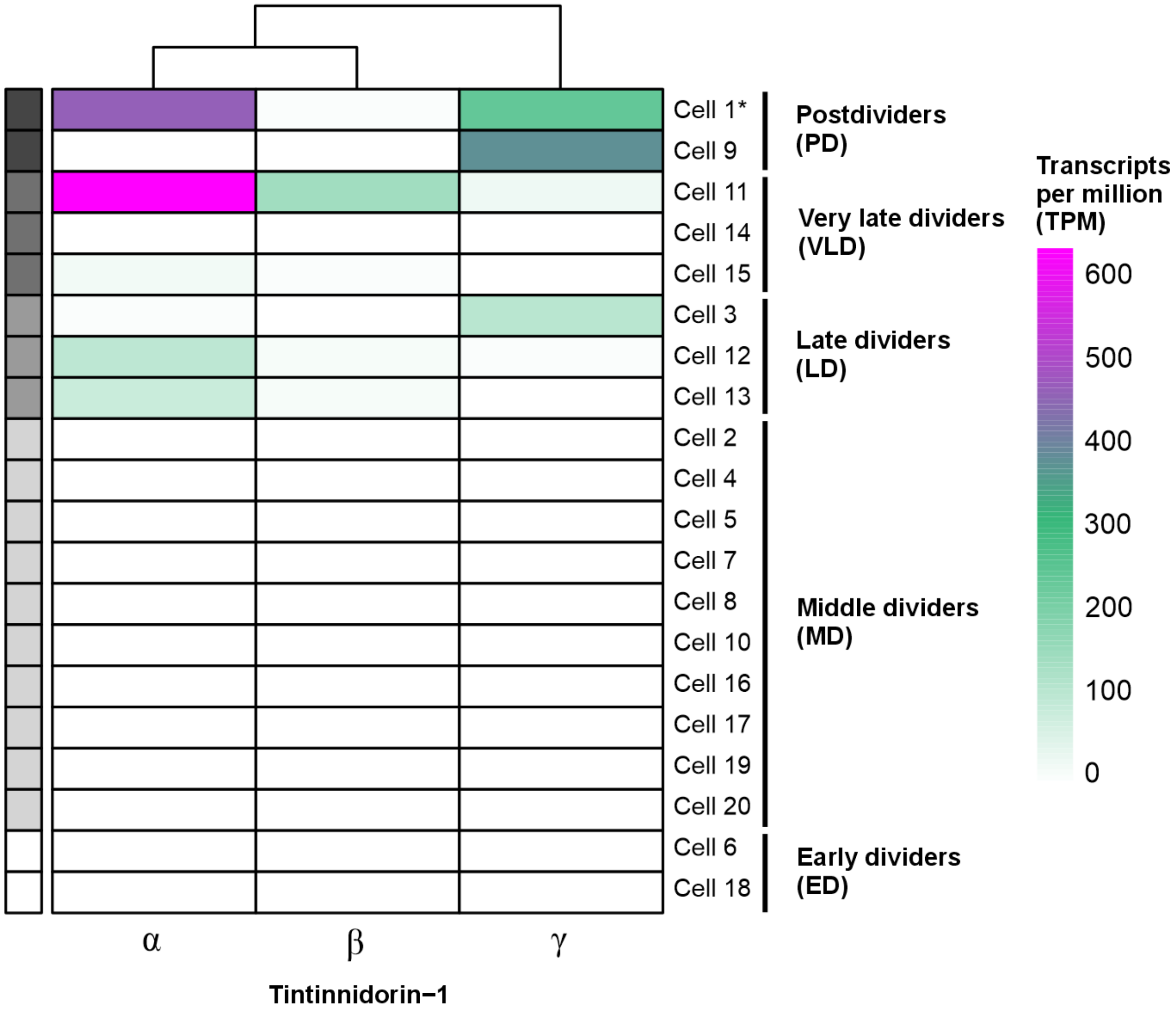

Supplementary Fig. 1 Relative gene expression of Tintinnidorin-1-alpha, beta, and gamma proteins during the cell cycle of monoclonal *Schmidingerella* specimens. The relative gene expression given in transcripts per million is highest in most late dividers (LD), very late dividers (VLD), and postdividers (PD), while earlier stages, namely, early dividers (ED) and middle dividers (MD), generally do not express the genes (Supplementary Data 3). High expression levels of Tintinnidorin are found in a specimen (asterisk; cell 1) that had no shell at the time of sampling but produced proteins potentially for a replacement shell. In another specimen, cell division was observed, and the posterior division product (cell 9) was picked. The three variants are not equally expressed, i.e., transcripts of Tintinnidorin-1-alpha and gamma are more abundant than those of Tintinnidorin-1-beta. Top branching illustrates the sequence similarity of the Tintinnidorin-1 proteins.

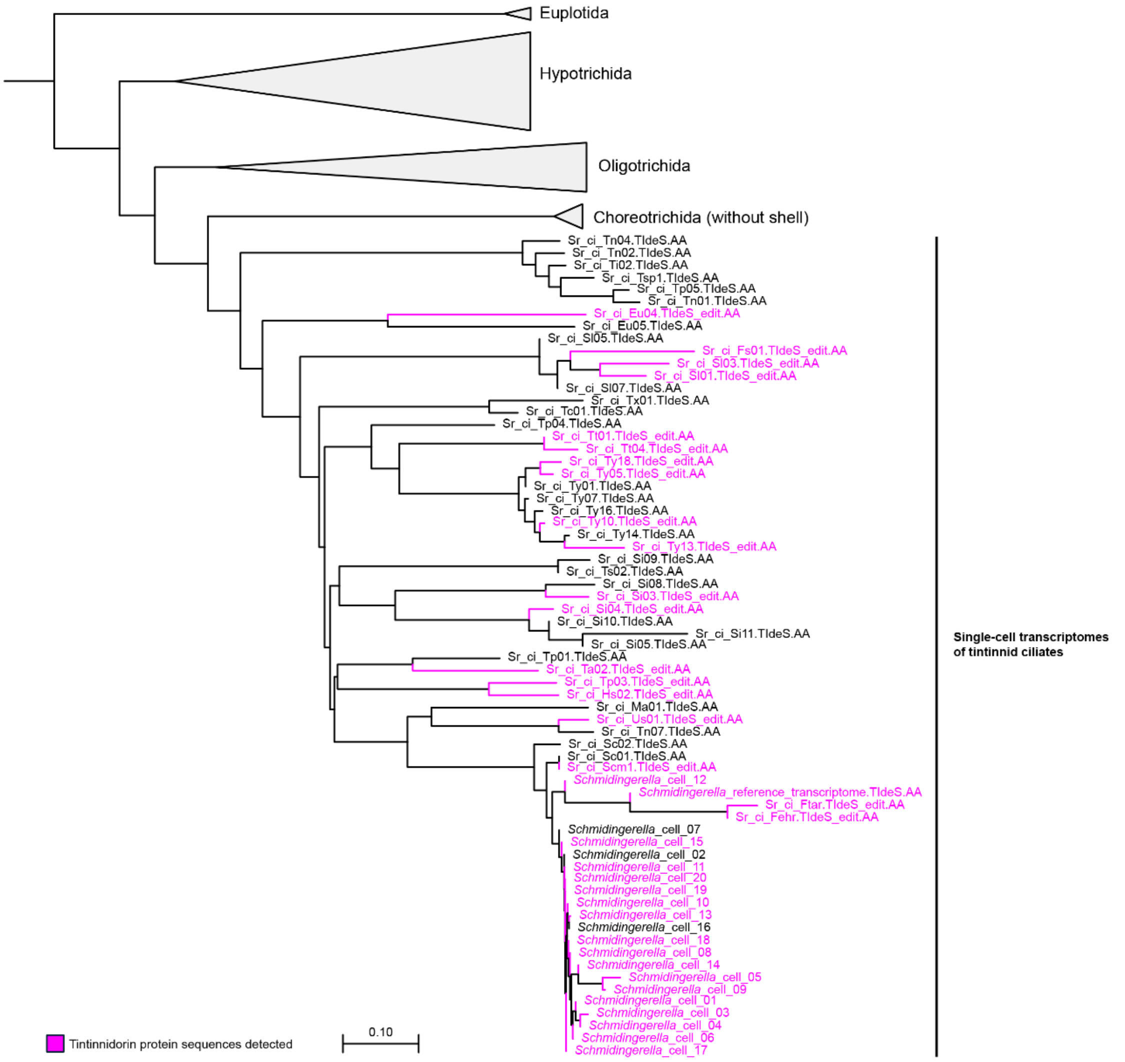

Supplementary Fig. 2 Species tree computed by Orthofinder2 based on 298 genomes and transcriptomes (Supplementary Data 5). Only the tree section including the closest relatives to tintinnid ciliates is displayed. Partial and complete Tintinnidorin protein sequences were exclusively detected in single-cell transcriptomes of tintinnid ciliates (magenta) and assigned to a single hierarchical orthogroup. The tree topology regarding Euplotida, Hypotrichida, Oligotrichida, and Choreotrichida perfectly match phylogenies based on the analyses of nuclear marker genes. Scale bar represents the average number of substitutions per site for a unit branch length.

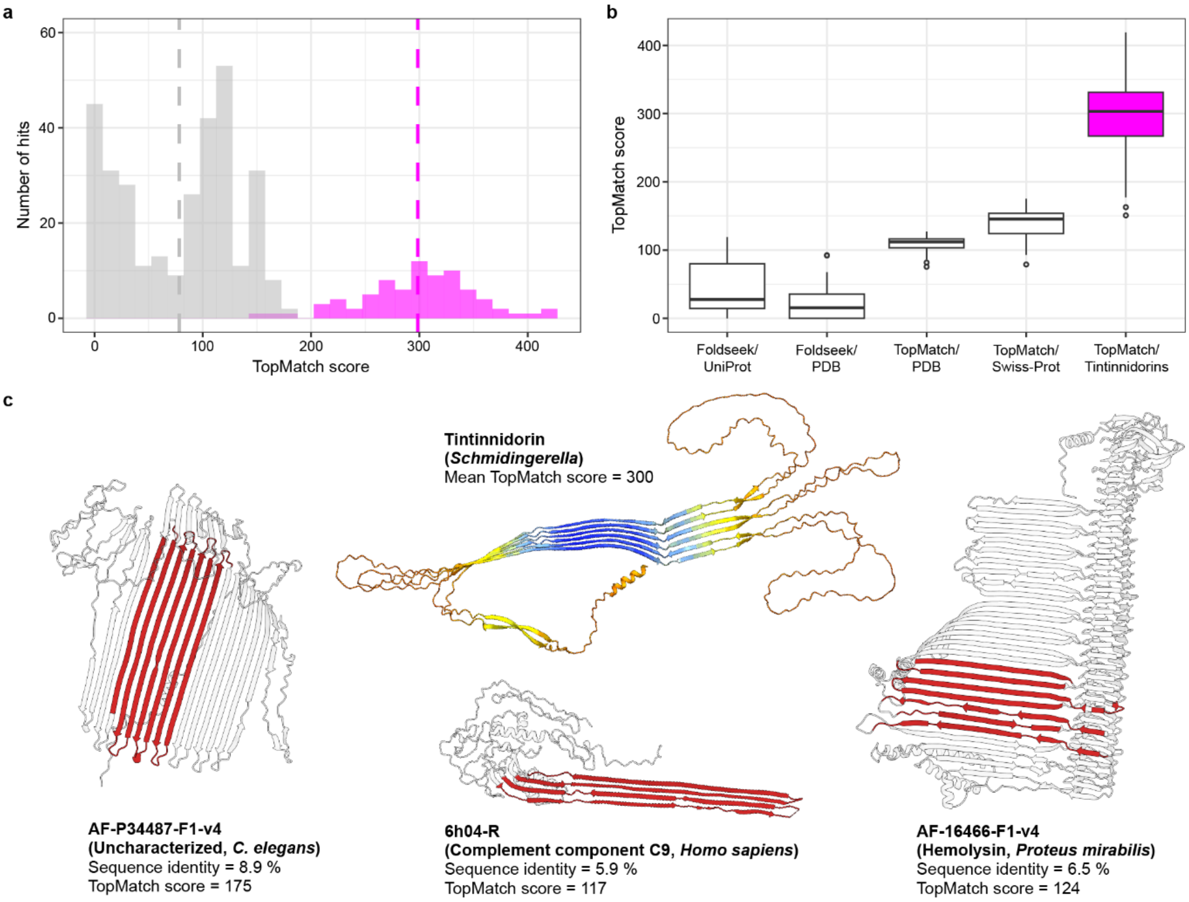

Supplementary Fig. 3 Structure similarities of the 78 full-length Tintinnidorin proteins. a. Distribution of structure similarity scores as calculated by TopMatch to the best matching targets in exhaustive structure searches against Protein Data Bank (PDB) and UniProt (grey) and in all-against-all structure comparisons of the 78 Tintinnidorin tintinnid shell proteins (magenta). Dashed lines indicate the mean scores for the respective comparisons. While the overall best match of a Tintinnidorin structure to a PDB/UniProt protein structure gets a similarity score of about 175 (Supplementary Data 6), the extent of structure similarity between pairs of Tintinnidorin proteins is, on average, almost twice as high and mainly caused by the structural equivalence of the modules' beta-sheets, with score variance mainly reflecting loop disorder in the linkers. **b.** Distribution of TopMatch structure similarity scores categorized by search method and target database. **c.** Structure models of the three best matches to Tintinnidorin proteins based on the aligned segments shaded in red. The low sequence identities and TopMatch scores show that Tintinnidorin proteins have no structural homologs in any protein database.

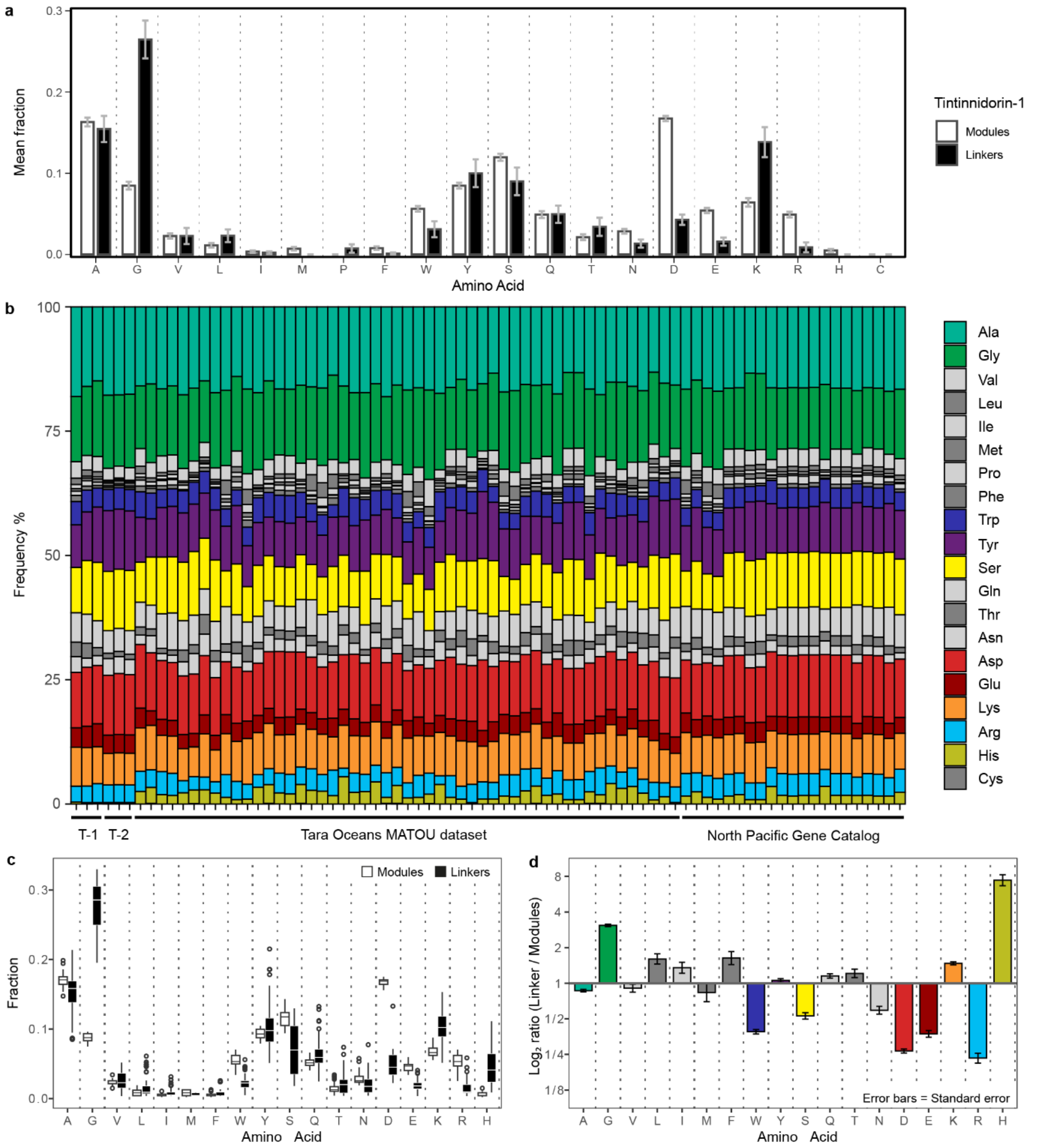

Supplementary Fig. 4 Comparisons of amino acid compositions. a. Comparisons of the amino acids' mean fractions between modules (n = 24) and linkers (n = 15) in the Tintinnidorin-1-alpha, beta, and gamma proteins of *Schmidingerella*. Error bars = standard error. **b.** Amino acid frequencies in the 78 full-length Tintinnidorin proteins, including Tintinnidorin-1 of *Schmidingerella*, Tintinnidorin-2 of *Tintinnopsis cylindrica*, and the sequences discovered in the Tara Oceans database and North Pacific Eukaryotic Gene Catalog, demonstrate the compositional similarity of the sequences. **c, d.** Comparisons of amino acid compositions between modules (n = 468) and linkers (n = 390) in the 78 full-length Tintinnidorin proteins: fractions (c), Log_2 ratios of average linker and module fractions (d). T-1, Tintinnidorin-1; T-2, Tintinnidorin-2; A, Ala, alanine; G, Gly, glycine; V, Val, valine; L, Leu, leucine; I, Ile, isoleucine; M, Met, methionine; P, Pro, proline; F, Phe, phenylalanine; W, Trp, tryptophan; Y, Tyr, tyrosine; S, Ser, serine; Q, Gln, glutamine; T, Thr, threonine; N, Asn, asparagine; D, Asp, aspartic acid; E, Glu, glutamic acid; K, Lys, lysine; R, Arg, arginine; H, His, histidine; C, Cys, cysteine.

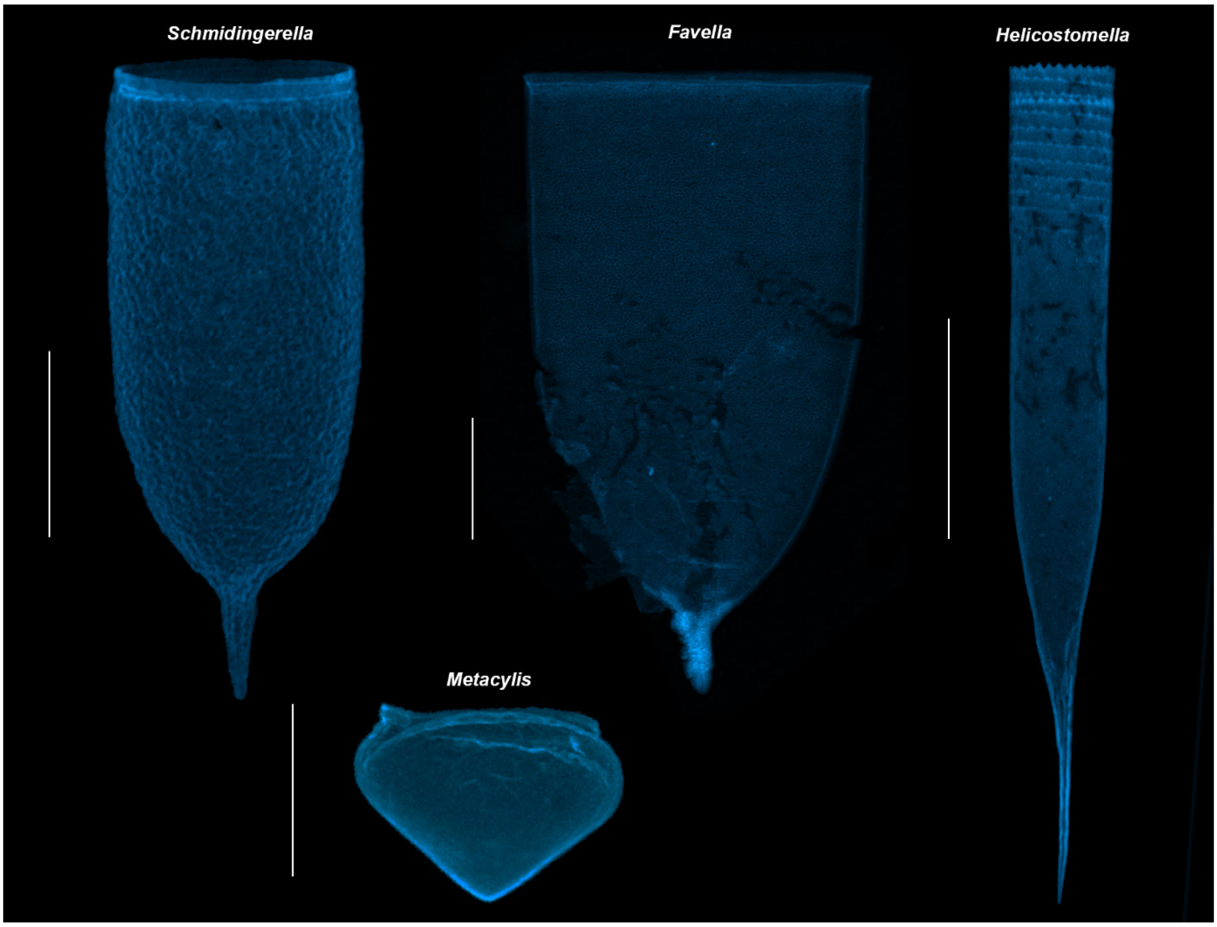

Supplementary Fig. 5 Autofluorescence of tintinnid ciliate shells excited with UV light. The optically transparent shells of *Schmidingerella*, *Favella*, *Helicostomella*, and *Metacylis* exhibit an emission at about 465 nm after an excitation at 385 nm. The shell of *Favella* is squashed due to the pressure of the cover slip. Images based on four independent measurements. Scale bars are 50 µm.

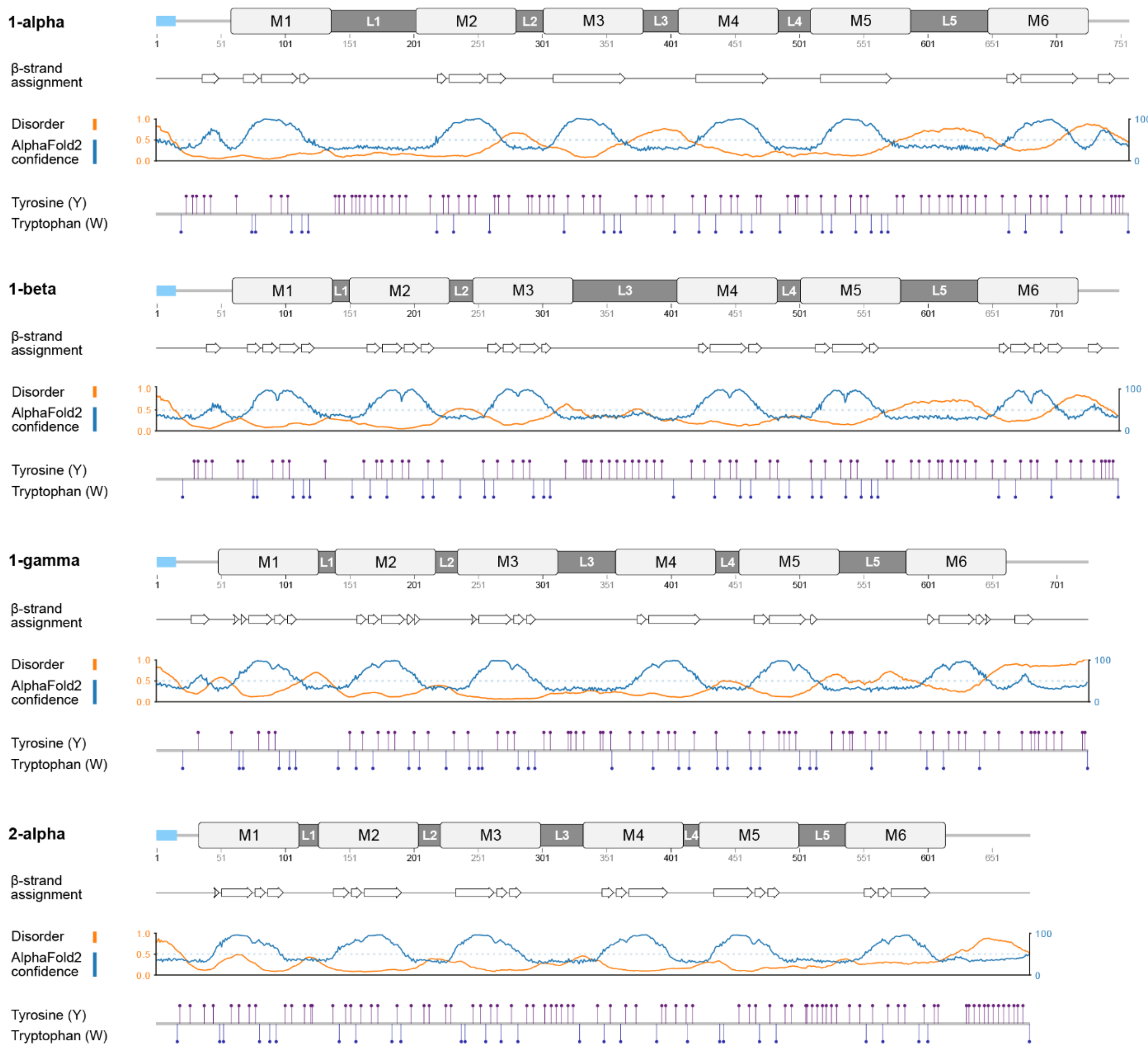

Supplementary Fig. 6 Sequence and structural characteristics for Tintinnidorin-1-alpha, beta, and gamma of *Schmidingerella* and Tintinnidorin-2-alpha of *Tintinnopsis cylindrica*. Upper lines: Schemes of the six modules (M1–6), the connecting linkers (L1–5), and the signal peptides (light blue). Second line: Positions of beta-strands as assigned by STRIDE[44]. Third line: Intrinsically disordered regions (orange) as predicted by metapredict V2[45] and AlphaFold2 (blue) per-residue confidence score (pLDDT, predicted local distance difference test). Disorder values (orange) higher than 0.5 (threshold = dotted line) indicate high probability for disorder. High AlphaFold2 model confidence values are generally predicted for the modules and concur with low values for disorder. Conversely, high values for disorder are mainly predicted for the linker segments, for which the model confidence values are low. Fourth line: Distributions of tyrosine (Y) and tryptophan (W) residues.

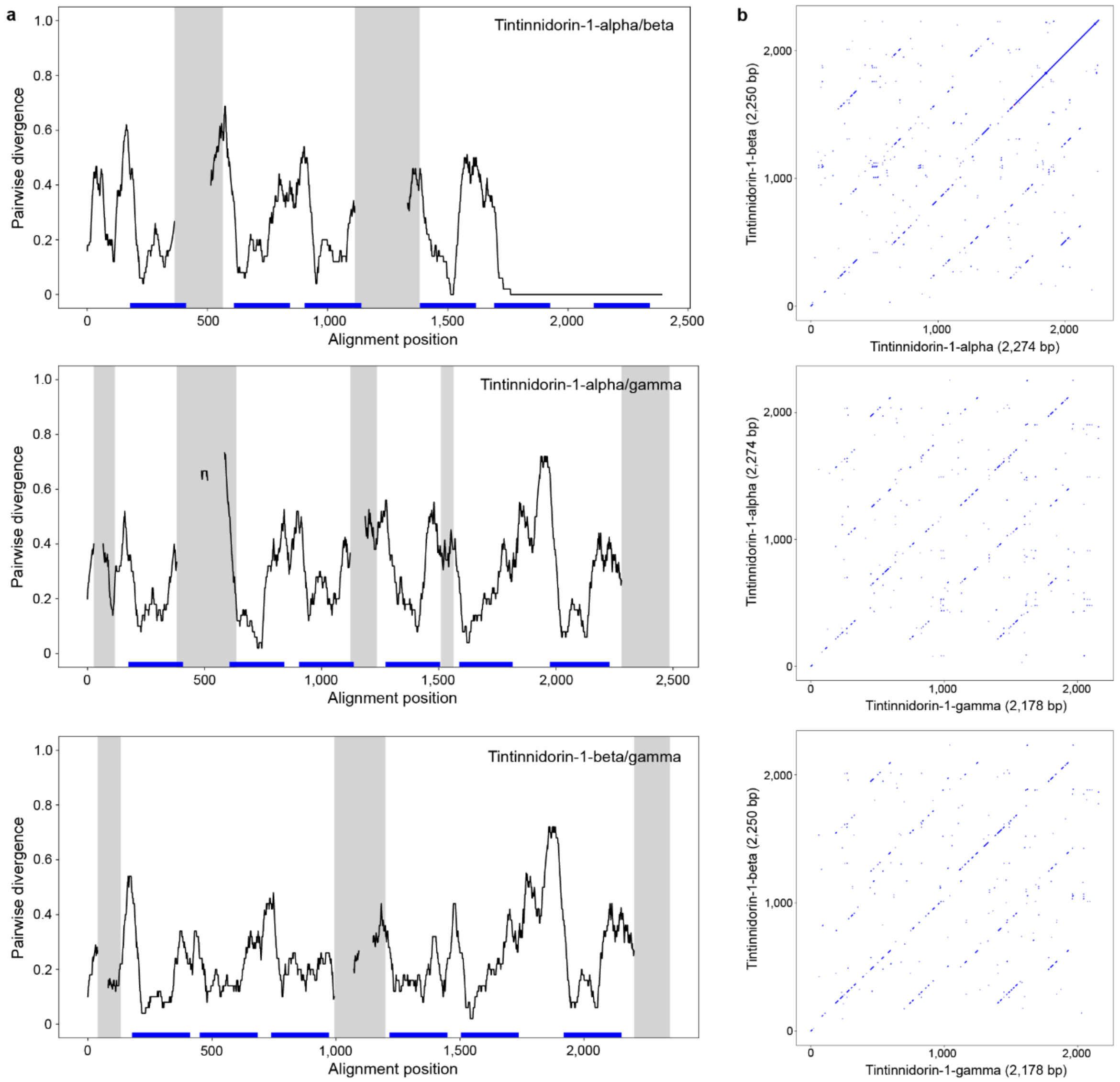

Supplementary Fig. 7 Pairwise nucleotide sequence divergence and similarity of Tintinnidorin-1-alpha, beta, and gamma. a. Sliding window analyses (50 base pairs with a step size of one base pair) of aligned nucleotide sequences of Tintinnidorin-1-alpha, beta, and gamma. Divergence is only partly plotted in the grey shaded alignment segments when one of the two compared sequences contains many gaps (≥ 20). Blue lines above the x-axes mark the six module positions in the alignment. **b.** Dotplots visualizing similarity between sequence pairs. Forward matches (blue dots) were identified, using a sliding window of 10 base pairs.

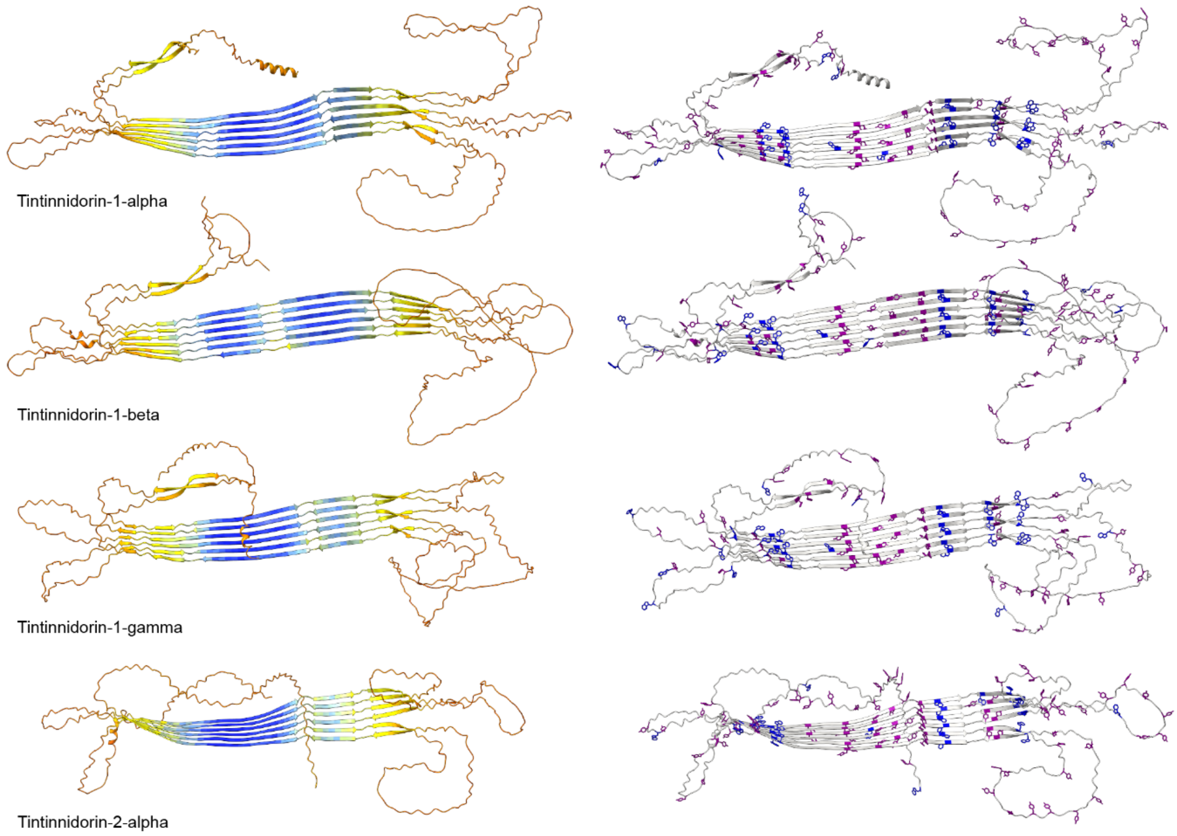

Supplementary Fig. 8 Structure models of Tintinnidorin-1-alpha, beta, and gamma from *Schmidingerella* and Tintinnidorin-2-alpha from *Tintinnopsis cylindrica* predicted by AlphaFold2. First column: Models color-coded by per-residue scores (pLDDT, predicted local distance difference test) ranging from high (blue) to low (orange) confidence values. Second column: Distribution of tyrosine (purple) and tryptophan residues (blue).

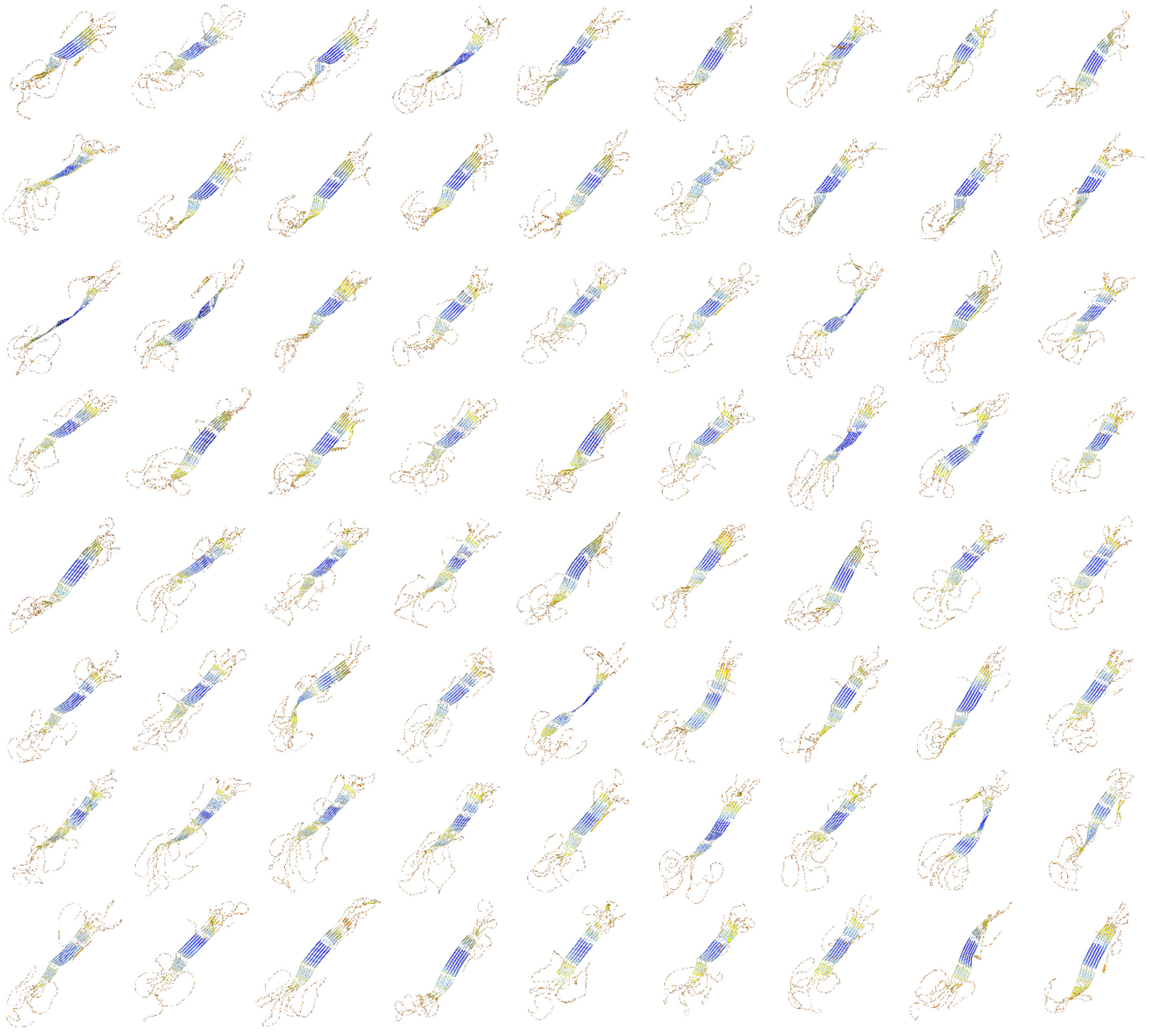

Supplementary Fig. 9 Structure models of the 72 full-length Tintinnidorin proteins extracted from the Tara Oceans database and North Pacific Eukaryotic Gene Catalog. The models are predicted by AlphaFold2 and color-coded by per-residue scores (pLDDT, predicted local distance difference test) ranging from high (blue) to low (orange) confidence values. Consistently, the core structure segments (antiparallel beta-sheets) are folded with the highest confidence, whereas the remaining segments are mostly disordered.

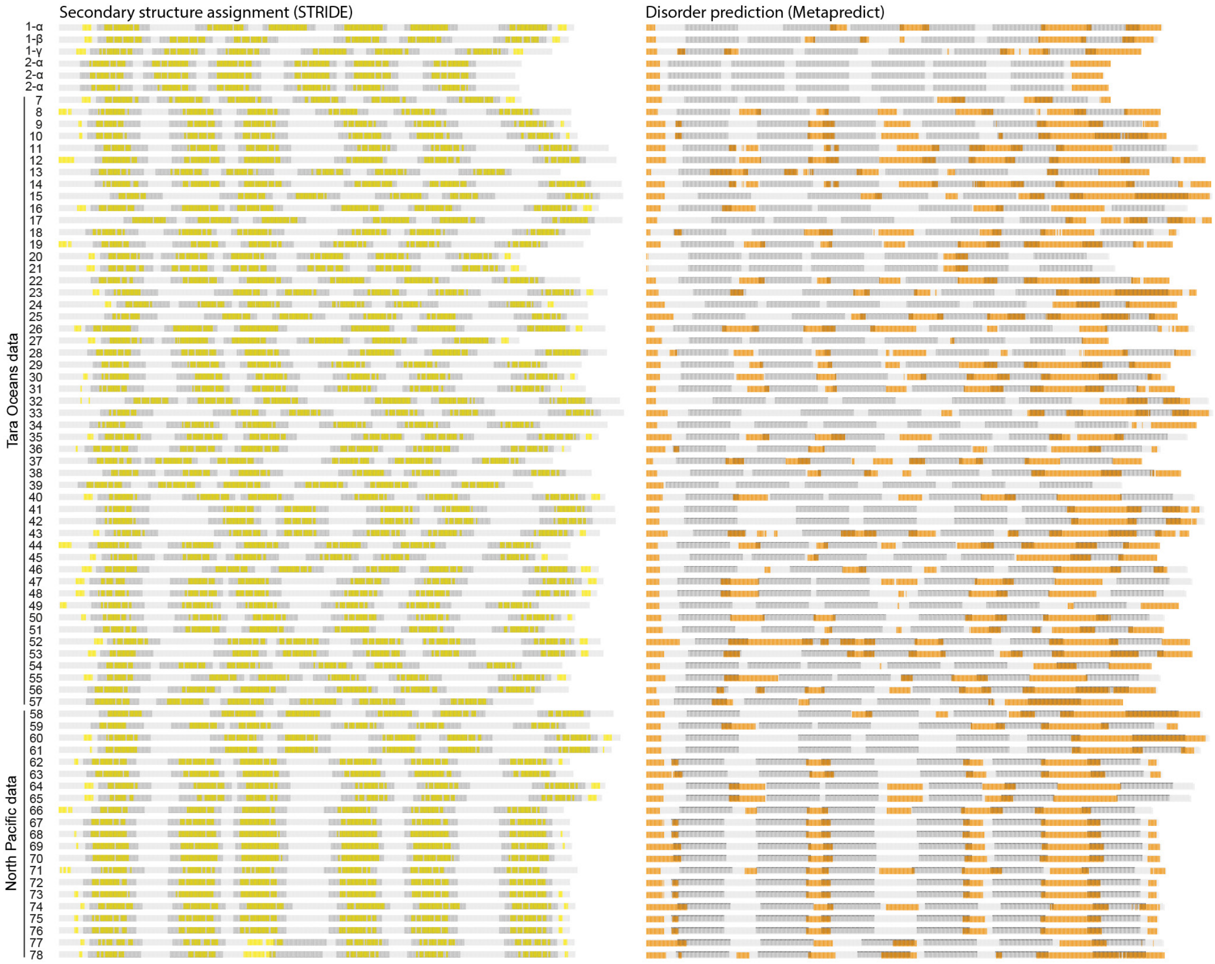

Supplementary Fig. 10 Secondary structure assignments and propensities for disorder in the 78 full-length Tintinnidorin proteins. Congruently, beta-sheets (left column; yellow) are assigned as the main secondary structure to the six modules of each sequence by STRIDE. In contrast, propensities for disorder (right column; orange) are predicted by metapredict V2 mainly for the N- and C-terminal segments and the linkers, especially for each most C-terminal linker. The sequence numbers refer to further information in Supplementary Data 7. 1-α, 1-β, 1-γ, Tintinnidorin-1-alpha, beta, and gamma of *Schmidingerella*; 2-α, Tintinnidorin-2-α of *Tintinnopsis cylindrica*.

Supplementary Fig. 11 Mass spectra [1-21] of Tintinnidorin-1 peptides identified by Casanovo and verified by secondary assignment of fragment masses in the proteomics data viewer PDV.

[1] Spectrum 17543

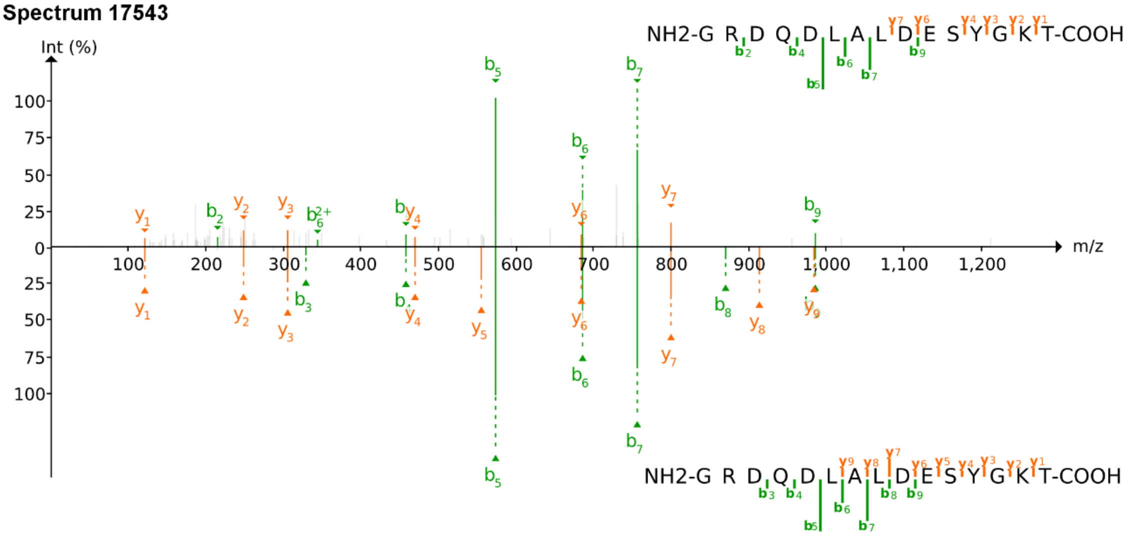

[2] Spectrum 27488

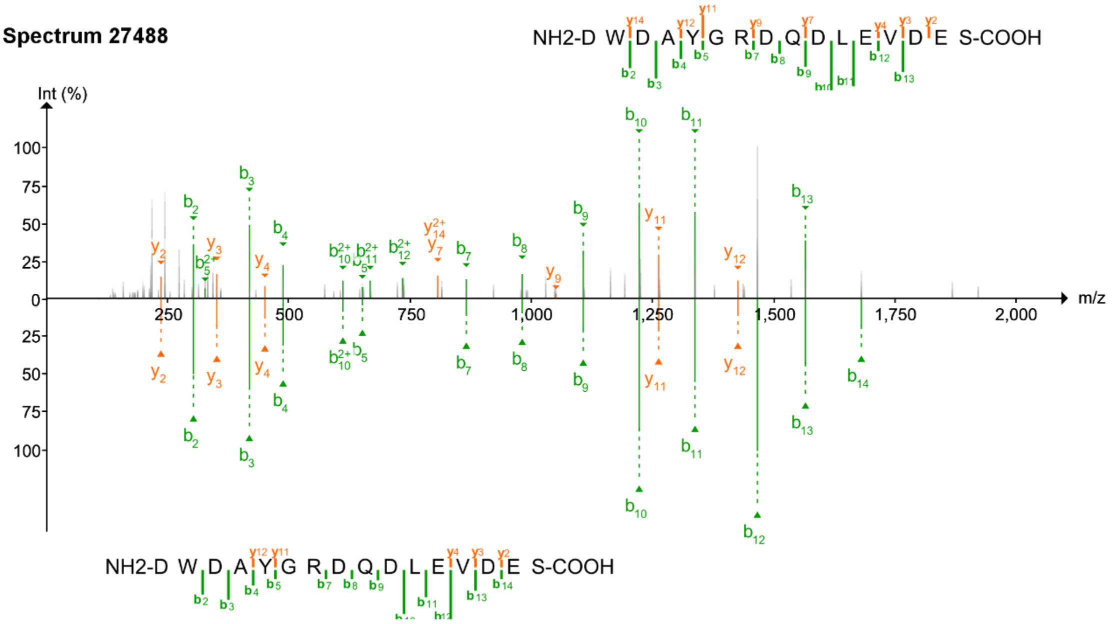

[3] Spectrum 22140

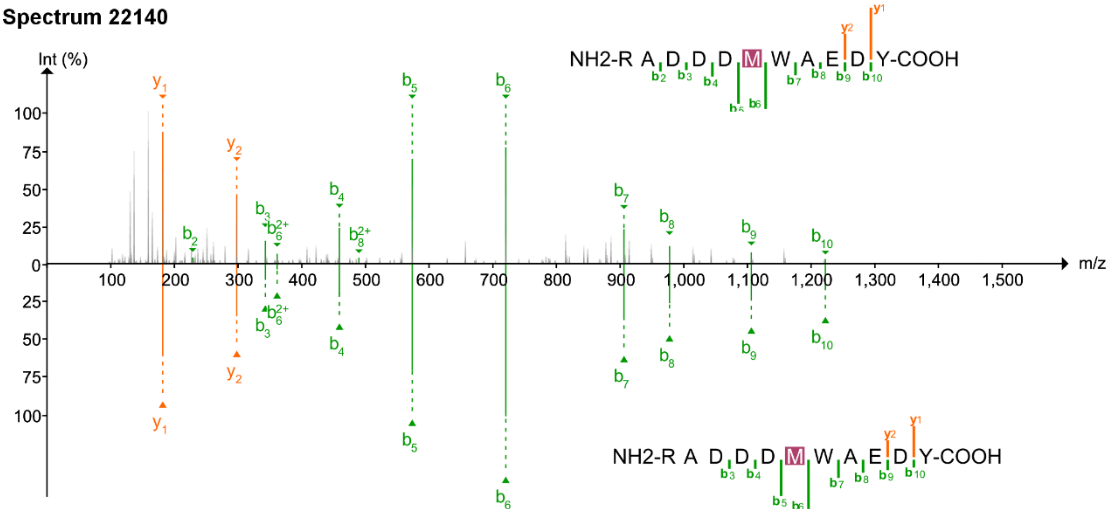

[4] Spectrum 20173

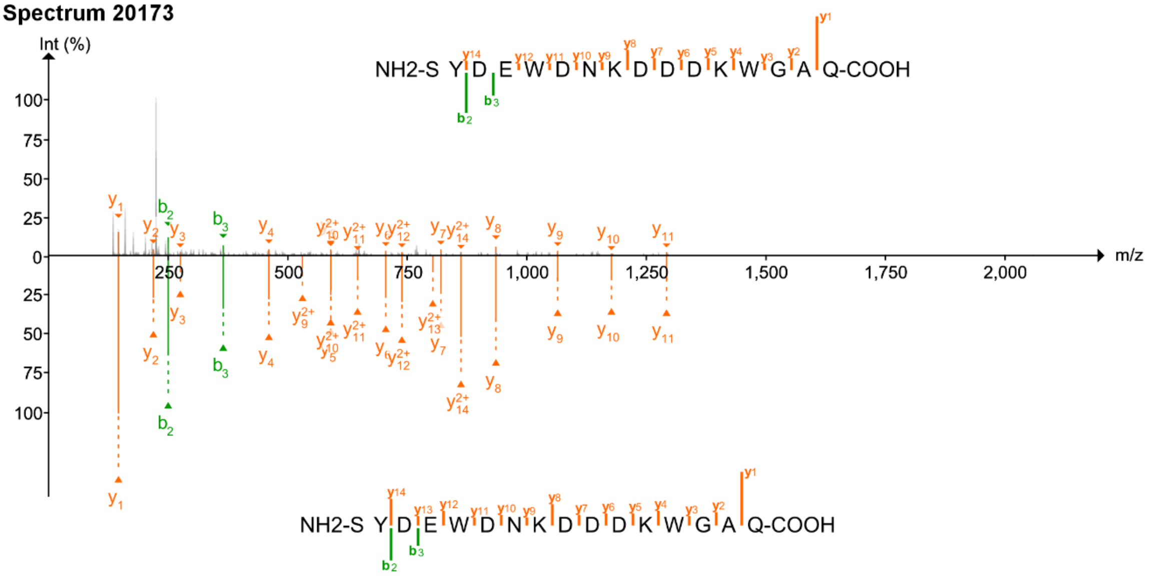

[5] Spectrum 18428

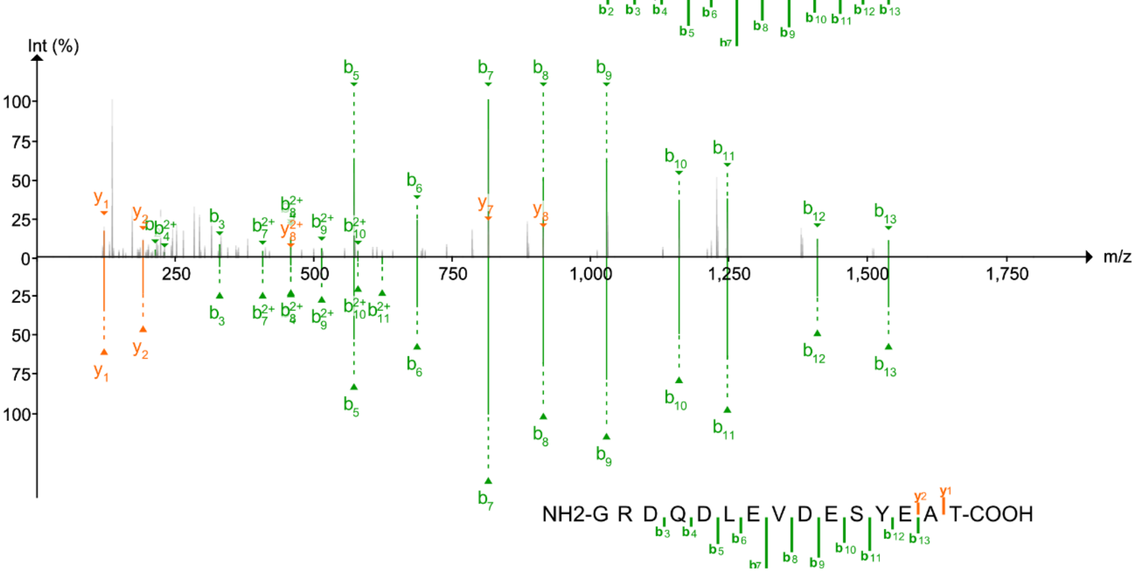

[6] Spectrum 11463

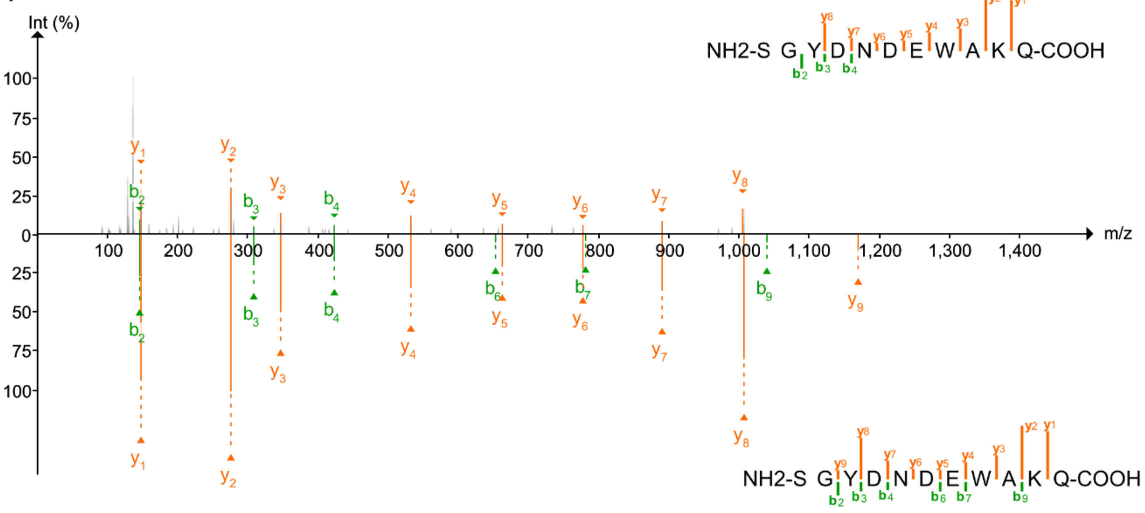

[7] Spectrum 10519

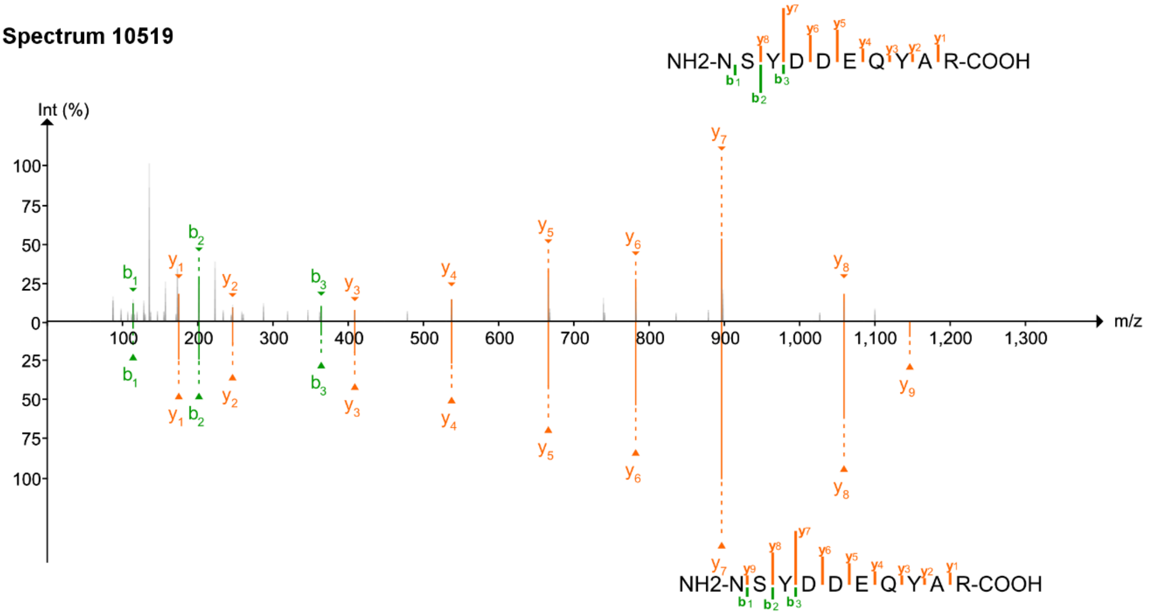

[8] Spectrum 14525

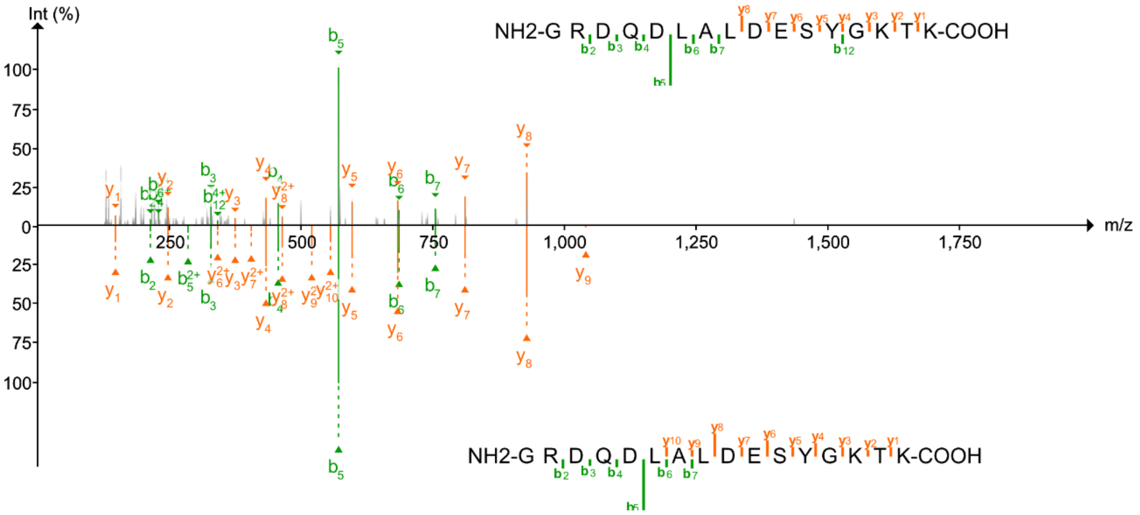

[9] Spectrum 32767

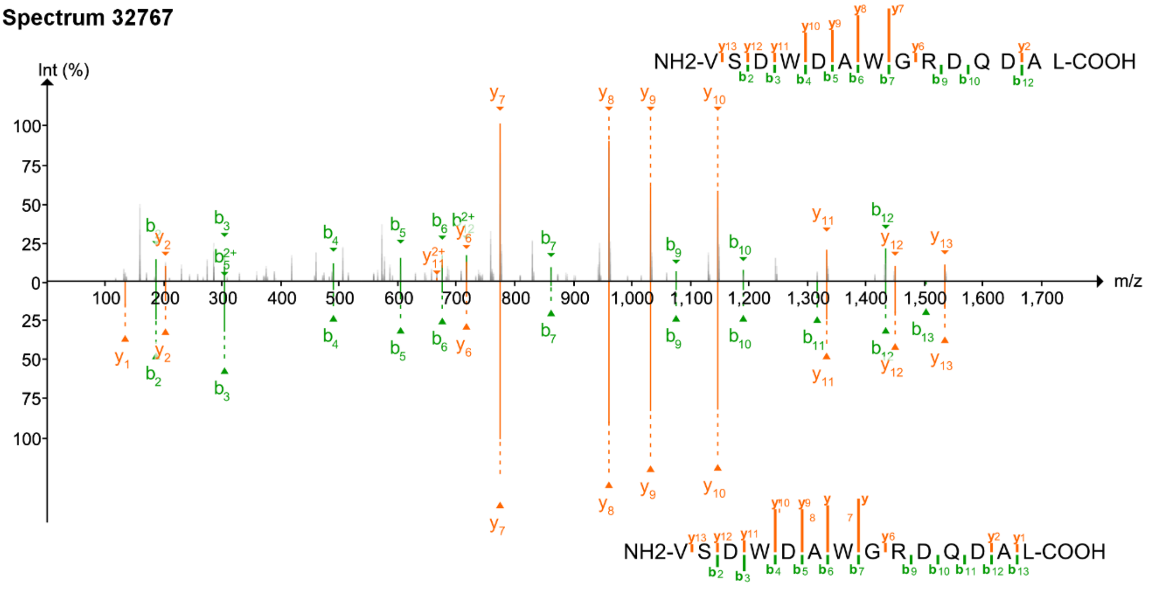

[10] Spectrum 36375

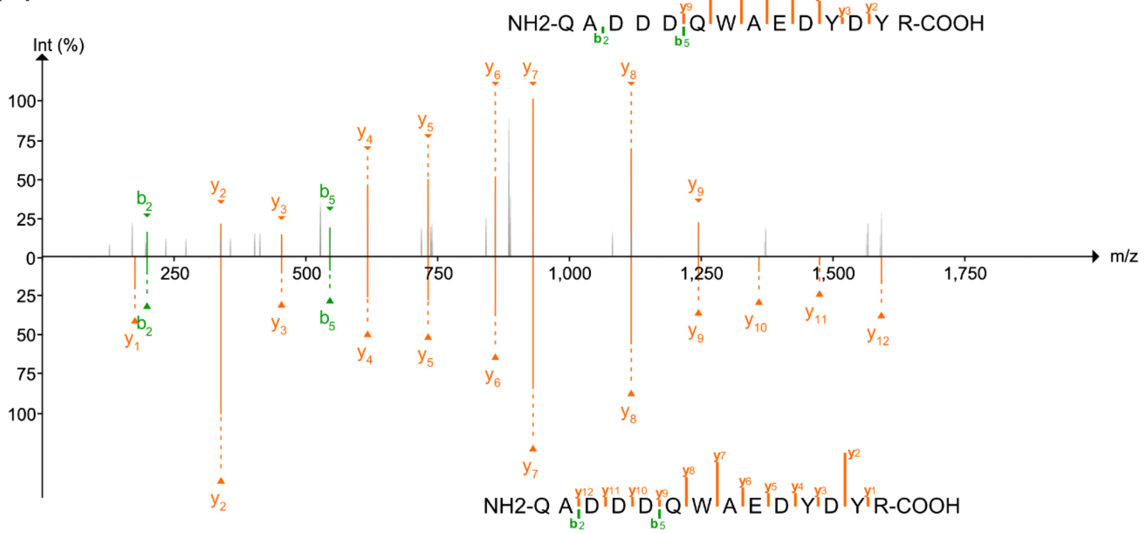

[11] Spectrum 28186

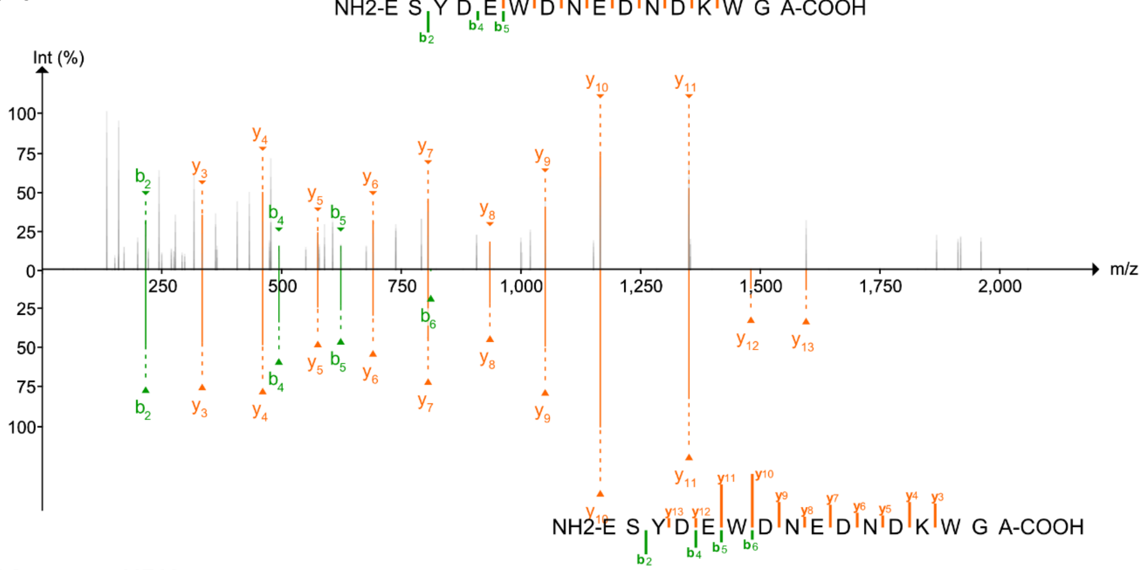

[12] Spectrum 11712

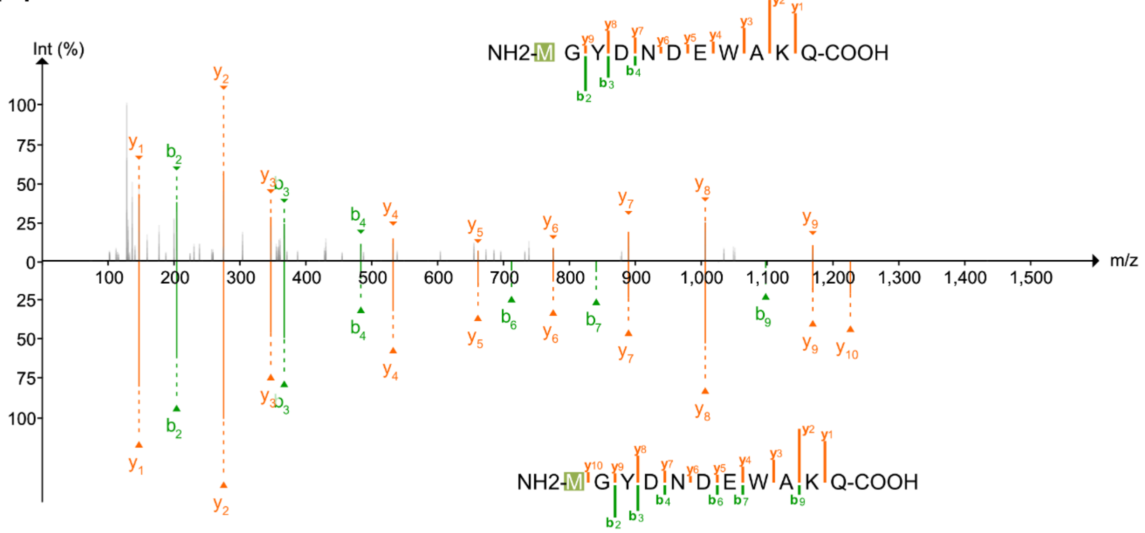

[13] Spectrum 20749

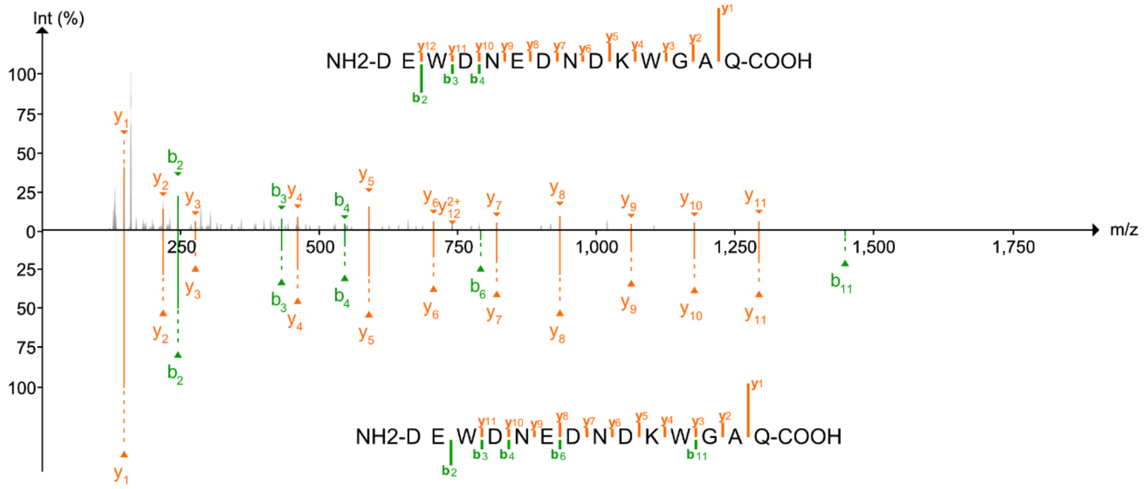

[14] Spectrum 17073

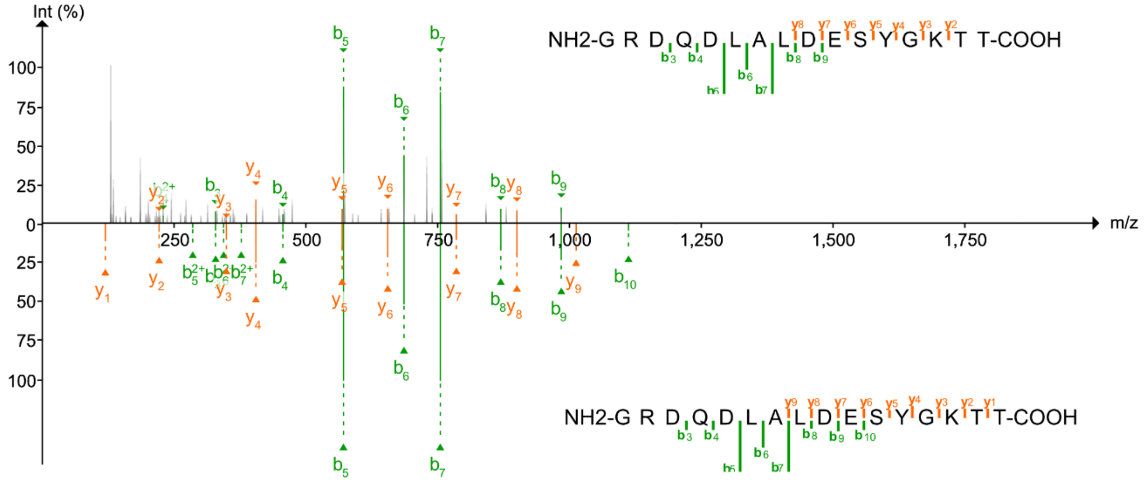

[15] Spectrum 23339

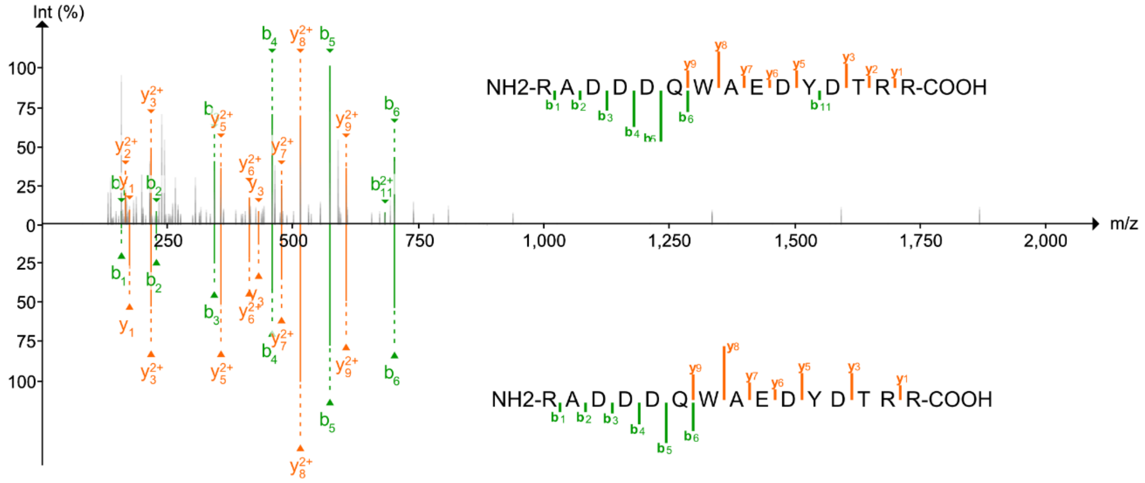

[16] Spectrum 25710

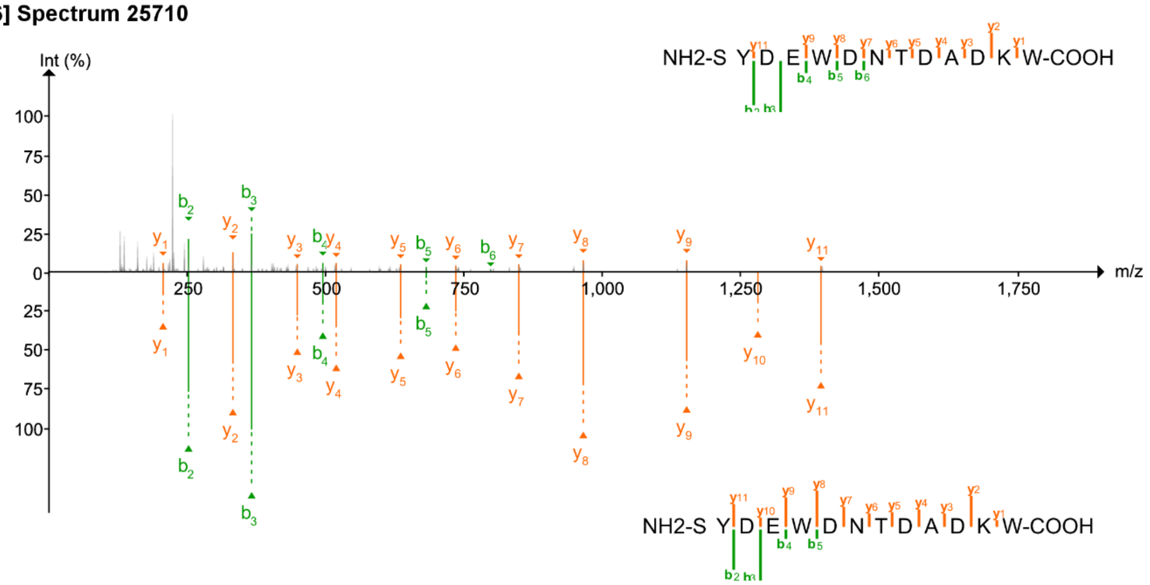

[17] Spectrum 12559

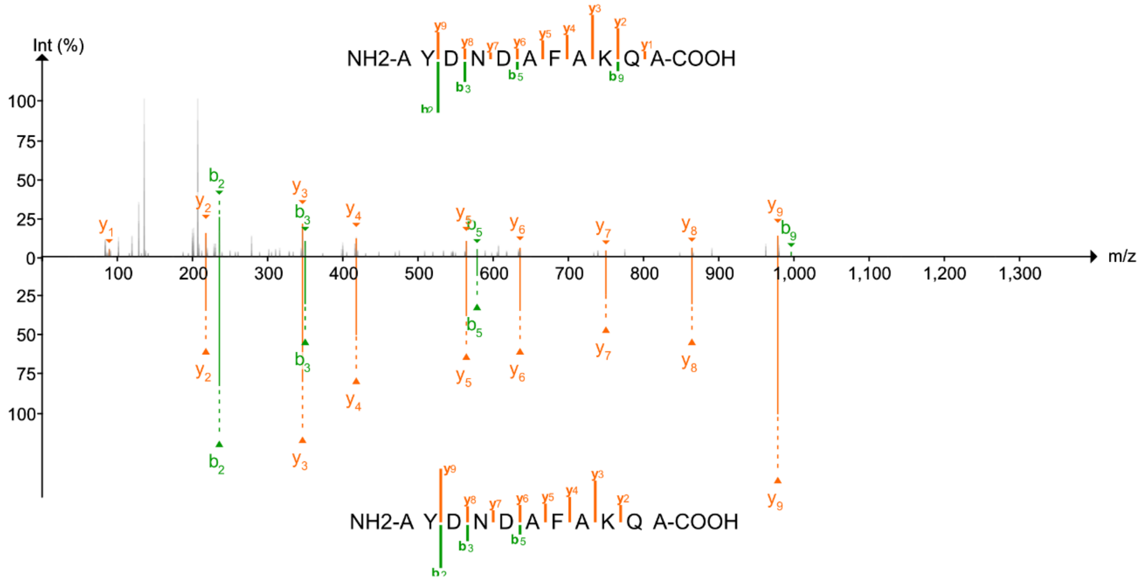

[18] Spectrum 29978

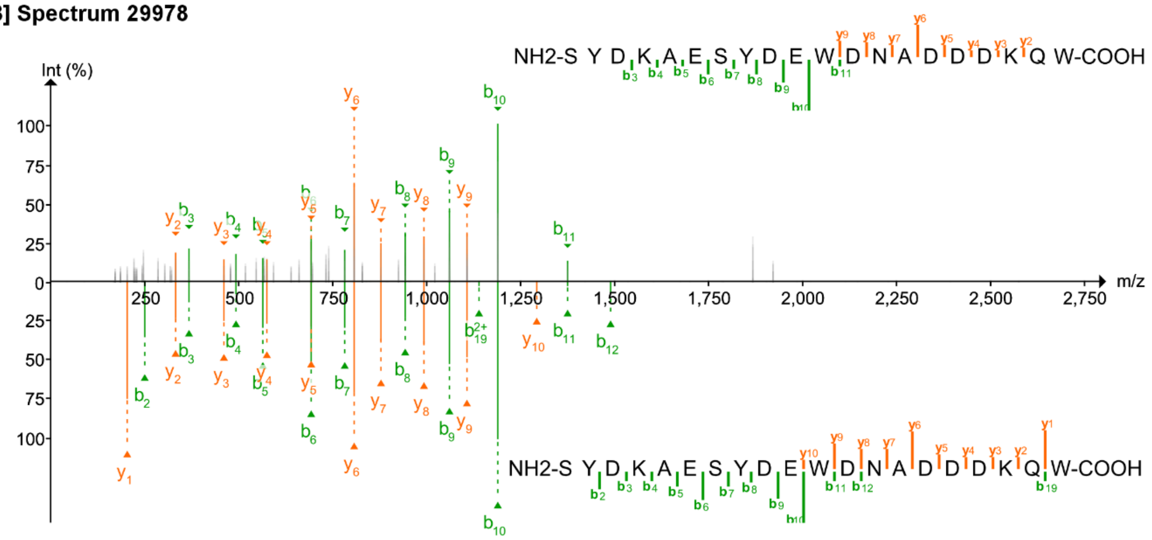

[19] Spectrum 23780

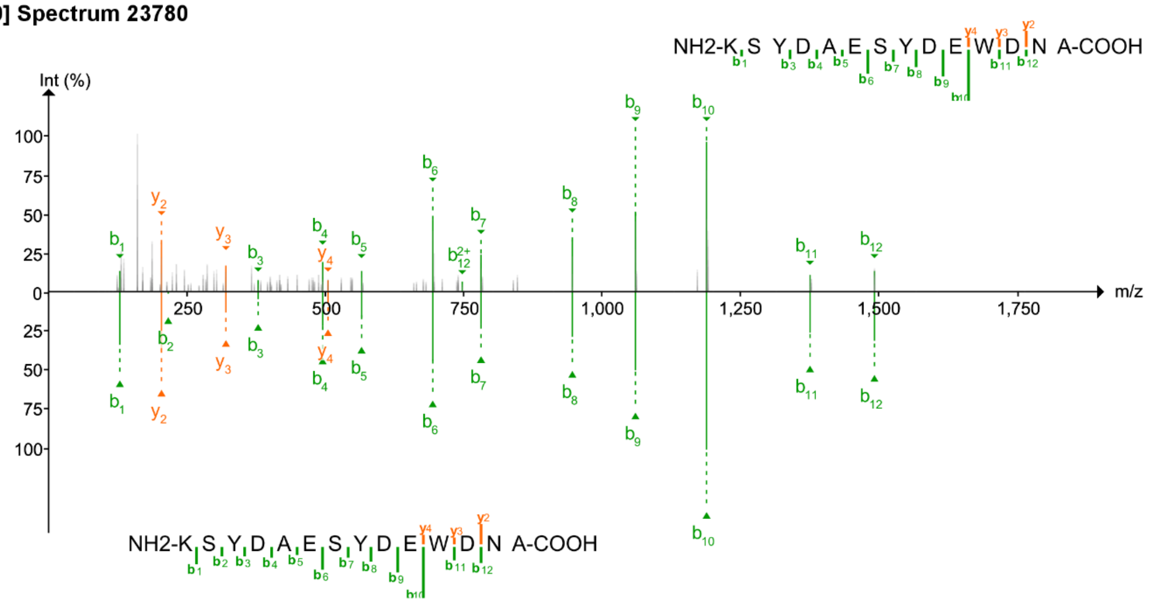

[20] Spectrum 17154

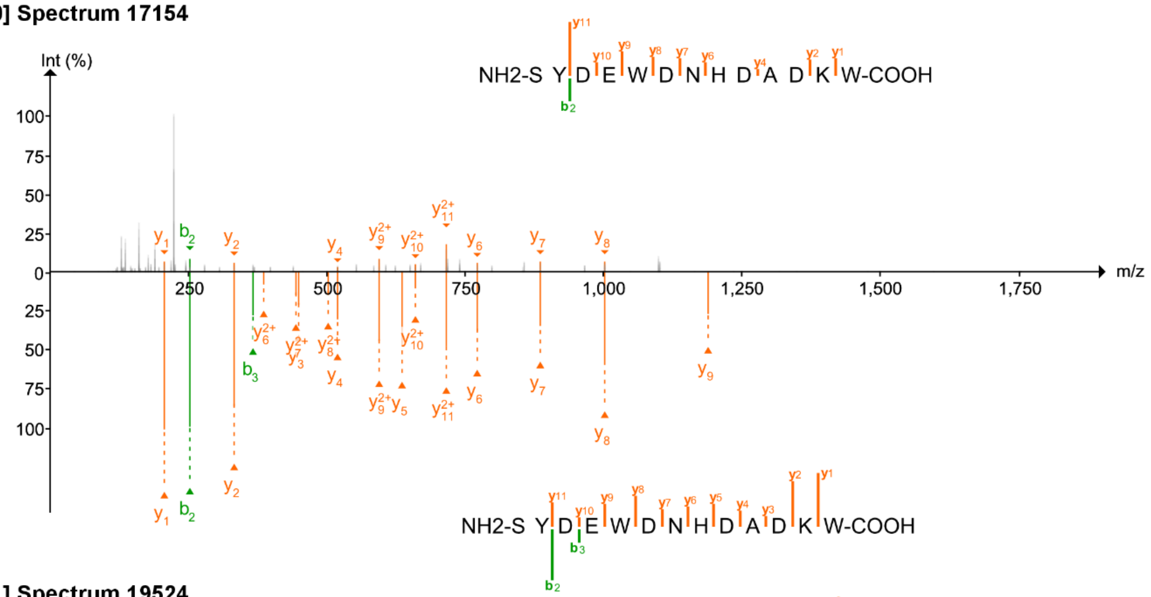

[21] Spectrum 19524

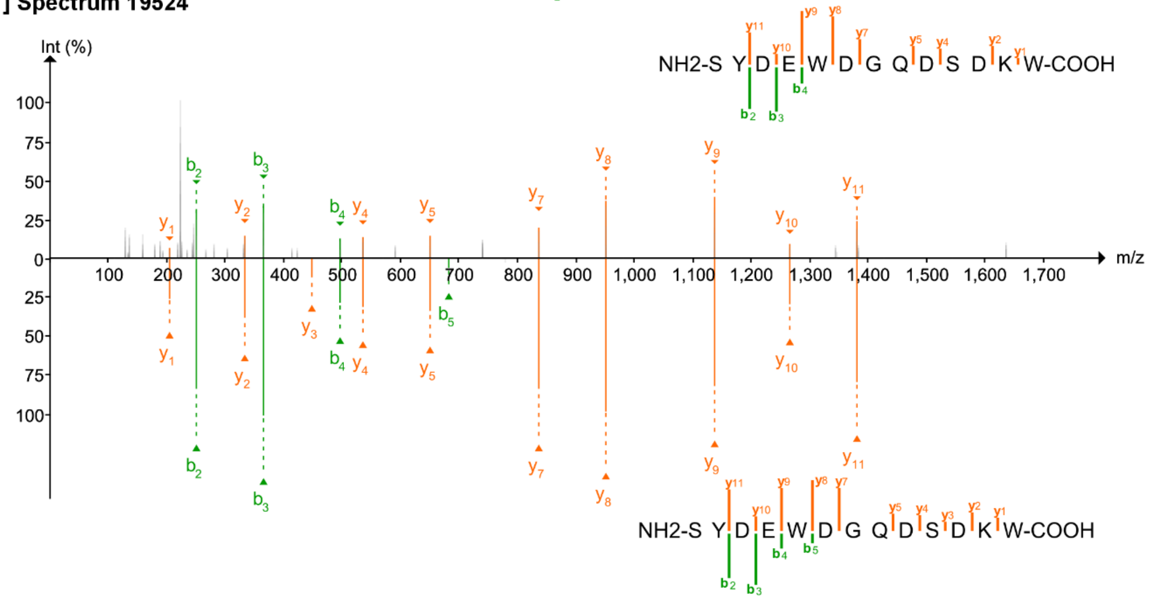

Supplementary Notes

Supplementary Note 1: Function of tintinnid shells

Several advantages of tintinnid shells accounting for both their comparatively high species richness and their global, predominantly marine distribution have been proposed, most of which relate to protection and feeding. Although the construction and transport of the shell impose additional energetic costs, its weight is frequently advantageous. From a protective perspective, the shell may operate through multiple mechanisms. In contrast to previous speculations[1], the present study provides the first evidence for the shells' partial shielding against ultraviolet radiation due to the comparatively high amounts of amino acids with aromatic residues and an autofluorescence after excitation at these wavelengths. Further, the shell may reduce the exudation of chemical cues detectable by predators[2]. Shells also facilitate rapid sinking of a contracted tintinnid out of a predator's attack trajectory[2] and increase the likelihood that a tintinnid contracted into its shell will be released by predatory copepods[3,4]. Nevertheless, these protective mechanisms are not completely effective. Undamaged shells recovered from copepod fecal pellets demonstrate that tintinnid cells are digested like other ingested prey despite the high resistance of their shells[5] supposedly caused by the extracellular formation of beta-sheets by the Tintinnidorin proteins. A second suite of advantages concerns feeding. Experimental studies suggest that the weight of the shell, in combination with the apical ring of (collar) membranelles, stabilizes the organism with its apical end oriented upward. This creates a predominantly upward swimming direction that facilitates the exploitation of phytoplankton-rich surface layers[6]. Furthermore, advanced hydrodynamic experiments indicate that the shell increases drag, thereby strengthening the feeding current and enhancing the encounter rate with food particles[7]. These advantages might in concert have promoted the evolution of the tintinnid ciliates.

Supplementary Note 2: Properties of the shell biomaterial

The primary scope of the present study is the discovery and first characterization of Tintinnidorin proteins. Previously, several remarkable properties of tintinnid shells have been documented experimentally, clearly demonstrating the potential of this proteinaceous biomaterial. Agatha and Simon demonstrated that the shell-forming material of tintinnids with diverse shells exhibits a remarkable resistance to high temperatures (about 160 °C), strong acids and bases (hydrofluoric acid and potassium hydroxide), and various enzymatic treatments[8]. Further enzymatic digestion experiments were conducted in preliminary analyses of the present study (Supplementary Method 4).

During our live observations, just secreted Tintinnidorin granules frequently got in contact with the glass slide and were not removed by the vigorously fidgeting tintinnid cells[9]. While sticking on the slide, the material granules showed an expansion and merging, forming a material layer with small chambers resembling the natural shell wall texture (Supplementary Movie 1). Wet adhesion (adhesion of structures in the presence of liquids) is also displayed by agglutinated tintinnid shells, in which the shell-forming material secreted by the ciliate adheres foreign particles with different properties (calcium carbonate/calcite: coccoliths and mineral particles; silicate: diatom frustules and mineral particles). In Tintinnidorin, the most plausible explanation for wet adhesion are molecular interactions of the highly abundant tyrosine and lysine, as in marine animal biomaterials (e.g., mussel foot proteins). We consider these examples sufficient evidence for the Tintinnidorin's wet adhesive properties, although dedicated assays were not yet conducted.

The shell biomaterial self-assembly was also captured in a video of shell formation in free-swimming *Schmidingerella* tintinnids[9] and mentioned in previous light microscopical studies[10]. The capacity of the shell biomaterial to generate a considerable variety of layered wall ultrastructures is demonstrated by the diverse shell textures[11] (Fig. 1).

The tintinnid shell biomaterial likely exhibits photoprotective properties against ultraviolet radiation as indicated by the high content of aromatic amino acids in Tintinnidorin and the intrinsic autofluorescence of tintinnid shells (Supplementary Fig. 5).

Future studies investigating sequence-structure-material relationships, particularly using recombinantly expressed proteins, will be key to determine how Tintinnidorin properties can be reproduced and potentially tailored for specific biomaterial applications. Thereby, the natural variability displayed by the Tintinnidorin might even be enlarged.

Supplementary Note 3: Reorganization of membranellar zone in tintinnids
Renewal of the oral and locomotory structures (membranelles; ciliary fans) in tintinnid ciliates represents an intriguing biological question. Observations of live cells are limited by the difficulty of tracking a single proter across multiple cell divisions. In protargol-stained dividing and non-dividing specimens showing the nuclear apparatus and basal bodies, no indications of membranelle renewal or reorganization have been detected[12]. In several hypotrich ciliates, taxa relatively closely related to tintinnids and possessing a homologous zone of membranelles, partial reorganization of the oral ciliature during cell division and conjugation (reciprocal fertilization) has been documented[13,14]. Because the ciliature is resorbed during encystment in tintinnids and several hypotrichs[15], excystment necessarily involves its reconstruction and therefore renewal. Additional complexity is introduced by the capacity of some hypotrich ciliates for physiological reorganization in non-dividing cells, triggered by altered nutritional conditions, as well as post-traumatic regeneration following mechanical damage[14]. However, none of these processes has been observed in tintinnid ciliates or is directly associated with shell formation.

Supplementary Note 4: Nomenclature of Tintinnidorin proteins
Etymology: Tintinni-dorin. The protein is unique to the monophyletic tintinnid ciliates. The stem of the taxon's name is combined with the word "dorin", which has roots in different languages (Greek: dôron, neuter, noun). Its core meaning revolves around the concept of a gift handed down through generations.

Full-length Tintinnidorin proteins from single-cell transcriptomes of identified specimens were named according to a hierarchical naming system. Tintinnidorin and a numerical identifier provide a unique label, independent of the taxonomic nomenclature, and Greek letters indicate the variant within that group. Consequently, we named the shell proteins first discovered in *Schmidingerella* Tintinnidorin-1-alpha, beta, and gamma and those of *Tintinnopsis cylindrica* Tintinnidorin-2-alpha.

Supplementary Note 5: Supposed evolutionary mechanisms
The evolutionary mechanisms discussed for the tintinnids are derived from features related to the special genome architecture of ciliates, which possess two types of nuclei with different functions (nuclear dimorphism). This architecture is associated with

elevated rates of protein evolution, especially in ciliates with extensively fragmented genomes, such as tintinnids[16,17]. An extensive genome processing of the germline micronucleus during macronuclear reconstruction after conjugation produces thousands of gene-sized nanochromosomes in tintinnids and close relatives. During this process, micronuclear sequences are rearranged through the excision of internal eliminated sequences (IESs) and the assembly of macronuclear-destined sequences (MDSs), sometimes involving a complex genome scrambling[18,19]. Such mechanisms are suggested to promote rapid sequence diversification and the expansion of lineage-specific gene families[20]. In this context, the diversification of Tintinnidorin proteins may reflect repeated duplication and rearrangement events in the micronuclear genome followed by alternative processing into macronuclear nanochromosomes, potentially accounting for the large diversity of shells observed among tintinnids. However, detailed evolutionary hypotheses are difficult to formulate because genomic data from both the micronuclei and macronuclei are currently incomplete for *Schmidingerella*[16] and entirely missing for other tintinnids. High-quality assemblies of these two nuclear genomes from a single species are necessary to determine how Tintinnidorin genes are organized in the micronuclear genome and how they are processed into macronuclear nanochromosomes. Thereby, the evolutionary patterns underlying the diversification of this protein family will be elucidated.

Supplementary Note 6: Storage of structural proteins in tintinnids compared to animal silk glands

Our transmission electron microscopic analyses of the maturation process of shell-forming material granules containing the protein Tintinnidorin revealed distinct structural transitions[9]. We interpret these transitions as successive stages of a liquid–liquid phase separation (LLPS) process, in which two distinct phases are separated because of changing physicochemical conditions. The material in the mature granules appears to be stored in a highly compact state minimizing the volume while preventing unintended interactions between the proteins. These findings indicate that LLPS plays an important role for the storage of structural proteins in completely different biological systems, i.e., in tintinnid ciliates and also in the silkworm *Bombyx mori*[21] as well as in spiders[22]. A key distinction among these systems is the site of LLPS: in tintinnids, LLPS occurs within intracellular secretory vesicles, whereas in silkworms and spiders it takes place in the lumen of the silk glands. Conditions also differ markedly regarding pH. In tintinnids, LLPS proceeds at low pH (≤ 5) within the secretory vesicles, followed by a rapid increase in pH when the material is released into the (sea-)water after cell division. In contrast, the pH within the silk gland lumina decreases from approximately 8–7 in the proximal region to 6–4 distally. The main difference is that a controlled pre-assembly of silk proteins takes places before the fibres are formed by the spinning organ, whereas Tintinnidorin proteins start to self-assemble immediately when getting in contact with the surrounding (sea-) water.

Supplementary Note 7: Influence of ionic composition on material assembly

The environmental conditions during Tintinnidorin self-assembly in seawater are hardly comparable with those during protein pre-assembly in the lumen of silk glands where the silkworm regulates the pH and the metal ion concentrations[23].

In the seawater, the major ions show constant ratios in the salinity range of about 33–37‰ (Forchhammer's Principle[24]). In brackish waters, the ion composition fluctuates due to freshwater influence and biological processes. The total salinity

concentration affects the distribution ranges of tintinnid species, although euryhaline estuarine species are occasionally recorded[25]. Generally, genera exhibit a markedly greater tolerance to salinity gradients, some occurring across regions with elevated salinity levels (e.g., about 40‰ in the Red Sea) to low-salinity brackish environments (e.g., the Baltic Sea)[26]. Extreme examples are the genera *Codonella*, *Stenosemella*, and *Tintinnidium*, which even occur in freshwater lakes, still forming the genus-specific shells. In the present study, we have identified 78 full-length Tintinnidorin proteins from single-cell and metatranscriptome data. Congruently with microscopic data, these findings indicate a global distribution of tintinnids. If Tintinnidorin self-assembly depends on ions, they are thus apparently universally available in sufficient concentrations or can be replaced by equivalent ions. Future studies have to answer the question of how Tintinnidorin proteins manage self-assembly under such contrasting conditions.

Supplementary Note 8: Origin of images in Figure 1

Images in figure 1 are either original, owned by the authors, or reproduced with permission.

Figure 1b: Shell image used with permission of John Wiley & Sons, Ltd, from 'The Biology and Ecology of Tintinnid Ciliates' (eds Dolan, J. R. et al.), Agatha, S., Laval-Peuto, M. & Simon, P., 2013; permission conveyed through Copyright Clearance Center, Inc

Figure 1c,f: Shell images reproduced from 'A comparative ultrastructural study of tintinnid loricae (Alveolata, Ciliophora, Spirotricha) and a hypothesis on their evolution', Agatha, S. & Bartel, H., Journal of Eukaryotic Microbiology 69, 2022, according to the CC BY 4.0 requirements (https://creativecommons.org/licenses/by/4.0/).

Figure 1e: Shell image reproduced from 'A light and scanning electron microscopic study of the closing apparatus in tintinnid ciliates (Ciliophora, Spirotricha, Tintinnina): a forgotten synapomorphy', Agatha, S., Journal of Eukaryotic Microbiology 57, 2010; permission conveyed through Copyright Clearance Center, Inc. Image of shell section used with permission of Elsevier Science & Technology Journals, from 'Traité de Zoologie. Infusoires Ciliés, Systématique', Laval-Peuto, M., 1993; permission conveyed through Copyright Clearance Center, Inc.

Supplementary Methods

Supplementary Method 1: Species identification in *Schmidingerella*

Tintinnid taxonomy is largely based on shell characteristics, including shape, structure, and size. Most original species descriptions were published roughly eighty to one hundred years ago[27,28], typically relying on very limited material, often only one to five specimens. Consequently, the natural range of morphological variability was not captured. In many cases, only shell length was reported, which is problematic because length is now known to vary substantially among conspecific individuals, whereas the opening diameter is comparatively constant[29] and thus a more reliable diagnostic feature. Accordingly, this character must be inferred from line drawings, using given scale bars or magnifications, or from the ratio of shell length to opening diameter, both very imprecise approaches.

In the genus *Schmidingerella* (formerly included within *Favella*), inferred shell opening diameters span an exceptionally wide range in some species, whereas in most species only single values have been deduced. Because of substantial overlap in opening diameters and the overall similarity in shell morphologies, the taxonomy of this tintinnid genus requires a comprehensive integrative revision. Such a revision should

include analyses of a substantial number of monoclonal strains from different localities, examining shell morphology and ultrastructure, cell features, particularly the ciliary pattern, and multiple genetic markers. Only with these data will it be feasible to clearly distinguish intraspecific variability from interspecific similarity.

Previously, *Schmidingerella* specimens collected in the Northeast Pacific were generally assigned to *S. meunieri* [11,15,19,30-33], whereas those from the Northwest Atlantic were identified as *S. arcuata*[19,34]. Barcoded specimens from these regions exhibit minor differences in ribosomal marker gene sequences and show overlapping ranges of shell characteristics. The variation in shell morphology among the Northeast Pacific strains obtained from the Strom laboratory suggests that more than one species may occur in this region. The marker gene sequences of the strain analysed in the present study match those of specimens previously identified as *S. meunieri* solely based on their Northeast Pacific origin[19]. Given the unresolved and problematic taxonomic situation, we refrain from assigning our specimens to a species.

Supplementary Method 2: Cultivation of tintinnid ciliates

The autecology of most tintinnid species, including their preferred prey, remains poorly understood. Consequently, establishing laboratory cultures is largely a process of trial and error and requires continuous access to fresh material. We repeatedly obtained monoclonal strains from the Strom laboratory. Increasing strain numbers indicated that new cultures were periodically initiated by isolating single *Schmidingerella* cells from field samples.

In the Strom laboratory, non-axenic cultures were maintained in 500-ml flasks containing 300 ml of ciliate medium, which were inoculated twice weekly and provisioned with 4 ml of the dinoflagellate *Heterocapsa triquetra*, half a Pasteur pipette of the prasinophyte *Mantoniella squamata*, and the haptophyte *Isochrysis galbana* (pers. commun.). The culture flasks were incubated at 15 °C with a 12:12 diel cycle of low light (about 20–30 µmol photons/m²/sec). The cultures were non-axenic and occasionally contained additional, unidentified flagellates at low abundances. *Heterocapsa triquetra* appeared essential for sustaining growth of our strains, and both this species and *I. galbana* have frequently been used in the cultivation of marine planktonic tintinnids[35].

We continued to culture the strains following the recommendations of the Strom laboratory and using *Heterocapsa triquetra* as the main food supplemented with *Isochrysis galbana*, provided as cultures. Under these conditions, *Schmidingerella* cultures could be maintained for up to six months. In several strains, conjugation was observed shortly before a pronounced decline and eventual collapse of the cultures. Depletion of prey organisms led to heavy bacterial infestation of the tintinnids, followed by culture termination. Application of these culture conditions to other tintinnid species is constrained by the limited knowledge of the species-specific autecological requirements.

Supplementary Method 3: Ribosomal RNA sequence assembly

Reads classified as rRNA sequences were assembled, using the same parameters as for the transcriptome assemblies. The resulting contigs were clustered with CD-HIT v4.8.1[36,37], using the CD-HIT-EST command at 95% similarity (Parameters: -c 0.95 -n 10 -d 0 -M 0 -T 0). The longest contig from each cluster was used to assess identity and completeness against a reference dataset of *Schmidingerella* rRNA sequences with BLASTN v.2.12.0. Contigs with high similarity to the reference sequences were

aligned with MAFFT, using the parameters --globalpair, --maxiterate 1000, and --adjustdirectionaccurately. Complete rRNA sequences were then reconstructed by concatenating overlapping contigs for each cell. Consensus sequences of the 18S, 5.8S, 28S rRNA genes and the internal transcribed spacer regions (ITS1 and ITS2) for our investigated monoclonal *Schmidingerella* specimens (SPMC 176) are available in NCBI GenBank under the accession numbers: PX559938 (18S SSU rRNA), PX559939 (ITS1-5.8S-ITS2), PX559940 (28S LSU rRNA). The present paper is the third publication in a series on shell formation in the model tintinnid ciliate *Schmidingerella*. Specimens of the same monoclonal culture (SPMC 176) had been investigated concerning the accumulation of shell-forming material during the cell cycle, its final volume available for shell formation, and a comparison of the maximum amount of intracellular material with the wall volume of the finished shell[12]. Further, the maturation and secretion of the shell-forming material and the process of shell formation were studied in the same clone[9].

Supplementary Method 4: Isolation of tintinnid shells for proteomics

In the 6-well plates used for culturing the monoclonal *Schmidingerella* tintinnids, empty shells sedimented after cell death and gradually accumulated at the bottom of the wells. Intact, empty, and optically clean shells (verified by inspection at 115× magnification) were collected with a finely drawn pipette approximately every two days to prevent bacterial colonization. The isolated shells were subsequently transferred through five to six rinsing steps in ddH_2O to remove residual contaminants and sea salts. After washing, the shells were placed in 1.5-ml Eppendorf caps with minimal water. With the lids left open, the caps were positioned in a desiccator to allow the shells to air-dry. Due to the labour-intensive nature of this procedure and the restricted number of shells meeting the required specifications, the about 1,700 shells selected for proteomics represent a significant proportion of the available material.

Supplementary Method 5: Interpretation of proteomic data

Combining single-cell transcriptomics and shell proteomics was key to obtain the full-length Tintinnidorin protein sequences and directly verify their presence in the shell biomaterial. To achieve this, several non-standard digestion approaches were employed, using both unspecific and more specific proteases (proteinase K, elastase, and trypsin). As these experiments represent the first proteomic analyses of tintinnid shell material, particular care was taken to use protease concentrations that were as low as possible relative to the unknown protein content of the shells. Proteinase K and elastase yielded promising results in preliminary tests. For these enzymes, we empirically determined the minimal concentration at which gradual shell decomposition could be confirmed visually by light microscopy. In contrast, several other proteases (e.g., α-chymotrypsin, trypsin, protease XIV) did not show detectable effects on the shells of *Schmidingerella*, even though potential cleavage sites for these proteases are present throughout the protein sequences, including modules, linkers, and terminal domains.

How Tintinnidorin proteins assemble into the shell biomaterial and how they are ultimately arranged within the hardened shell wall remains unknown. The architecture of this resistant proteinaceous biomaterial determines the accessibility of proteolytic cleavage sites, which depends on several structural factors that cannot be readily inferred from peptide fragmentation patterns in the mass spectra. Consequently,

standard assumptions derived from the digestion efficiency and cleavage patterns of soluble proteins are of limited applicability to such a biomaterial.

Because the shell proteins are novel to science, we employed a non-standard proteomics workflow in which peptide identification from mass spectra relied on a reference-free de novo approach rather than conventional database-driven matching. Peptide identification was likely further impeded by intra- and intermolecular crosslinking as well as extensive proteolytic digestion, which may generate atypical fragmentation patterns and very small peptide products, respectively. Even when the masses of such atypical fragments were detected, the corresponding spectra often could not be interpreted.

While the current experiments provide direct evidence for the identity of the shell biomaterial proteins, detailed structural inferences will require different approaches in future studies.

Supplementary Method 6: Homology search details

More extensive homology searches were conducted with EukPhylo v1.0[39]. Lineage-specific orthogroups identified with OrthoFinder were used to generate a database of tintinnid ciliate genes including Tintinnidorin proteins with DIAMOND v2.1.8[40]. The database was used to perform a systematic and sensitive search for potential homologs across a curated dataset of 1,000 diverse species of bacteria, archaea, and eukaryotes, utilizing the EukPhylo pipeline. The curated dataset comprised standardized genome and transcriptome assemblies[41] (https://doi.org/10.6084/m9.figshare.25336129.v2), some of which were generated from raw data in the sequence read archive (SRA) not otherwise available in GenBank.

BLAST searches of Tintinnidorin-1 sequences against the NCBI GenBank (last accessed October 2025) yielded hits to four sequences of hypothetical proteins with 54-66% sequence identity and partial coverage from a metagenomic dataset annotated by the NCBI Prokaryotic Genome Annotation Pipeline (Accessions: MCP4570280, MCP4287431, MCP4556353, and WP_288100621). The dataset originates from a study that investigated foraminifera and their associated microbiomes in marine sediments[42]. We assume, the sequenced samples must have contained DNA/RNA from tintinnid ciliate cells or their resting stages (cysts).

Additionally, we identified full-length Tintinnidorin sequences highly similar to those of *Schmidingerella* in the transcriptome data of the marine planktonic ciliate *Strombidinopsis acuminata* (Biosample accession: SAMN02740368). *Strombidinopsis* is closely related to tintinnid ciliates but does not form a shell. The species was cultured and sequenced along with tintinnid ciliate species, including *Schmidingerella* specimens, in the MMETSP project (Bioproject accession: PRJNA248394), and its transcriptome assembly shows high contamination with non-target sequences as evident from codon usage bias analyses in a recent phylogenomic study of planktonic ciliates[43]. In fact, the codon usage bias plot for *S. acuminata* (*Strombidinopsis* sp. MMETSP0126) displays many transcripts (grey dots; Figure S5 in ref. [43]) that match tintinnid ciliate transcripts regarding GC content at the third codon position (GC3) in contrast to transcripts attributed to *S. acuminata* (red dots). Consequently, we excluded this dataset from our analysis.

References

1. Armstrong, H. A. & Brasier, M. D. *Microfossils*. 2nd edn, i-vii + 1-296 (Blackwell Pub., 2005).
2. Capriulo, G. M., Gold, K. & Okubo, A. Evolution of the lorica in tintinnids: a possible selective advantage. *Ann. Inst. océanogr., Paris* **Suppl. 58**, 319–323 (1982).
3. Echevarria, M. L., Wolfe, G. V. & Taylor, A. R. Feast or flee: bioelectrical regulation of feeding and predator evasion behaviors in the planktonic alveolate *Favella* sp. (Spirotrichia). *J. Exp. Biol.* **219**, 445–456 (2016).
4. Stoecker, D. K. & Sanders, N. K. Differential grazing by *Acartia tonsa* on a dinoflagellate and a tintinnid. *J. Plankton Res.* **7**, 85–100 (1985).
5. Turner, J. T. Zooplankton feeding ecology: contents of fecal pellets of the copepods *Eucalanus pileatus* and *Paracalanus quasimodo* from continental shelf waters of the Gulf of Mexico. *Mar. Ecol. Prog. Ser.* **15**, 27–46 (1984).
6. Jonsson, P. R. Vertical distribution of planktonic ciliates - an experimental analysis of swimming behaviour. *Mar. Ecol. Prog. Ser.* **52**, 39–53 (1989).
7. Jiang, H. & Buskey, E. J. Relating ciliary propulsion morphology and flow to particle acquisition in marine planktonic ciliates I: the tintinnid ciliate *Amphorides quadrilineata*. *J. Plankton Res.* **47**, fbae012 (2024).
8. Agatha, S. & Simon, P. On the nature of tintinnid loricae (Ciliophora: Spirotricha: Tintinnina): a histochemical, enzymatic, EDX, and high-resolution TEM study. *Acta Protozool.* **51**, 1–19 (2012).
9. Ganser, M. H., Weißenbacher, B. & Agatha, S. How single cells form shells: maturation and secretion of lorica-forming material in the tintinnid *Schmidingerella* (Alveolata, Ciliophora). *J. Eukaryot. Microbiol.* **72**, e70025 (2025).
10. Laval-Peuto, M. Construction of the lorica in Ciliata Tintinnina. In vivo study of *Favella ehrenbergii*: variability of the phenotypes during the cycle, biology, statistics, biometry. *Protistologica* **17**, 249–272 (1981).
11. Agatha, S. & Bartel, H. A comparative ultrastructural study of tintinnid loricae (Alveolata, Ciliophora, Spirotricha) and a hypothesis on their evolution. *J. Eukaryot. Microbiol.* **69**, e12877 (2022).
12. Agatha, S., Weißenbacher, B., Böll, L. & Ganser, M. H. Morphologic changes in the model tintinnid *Schmidingerella* (Alveolata, Ciliophora) during the cell cycle, including the first volumetric analyses of the lorica-forming material. *BMC Microbiology* **25**, 88 (2025).
13. Agatha, S. & Foissner, W. Conjugation in the spirotrich ciliate *Halteria grandinella* (Müller, 1773) Dujardin, 1841 (Protozoa, Ciliophora) and its phylogenetic implications. *Eur. J. Protistol.* **45**, 51–63 (2009).
14. Berger, H. *Monograph of the Oxytrichidae (Ciliophora, Hypotrichia)*. Vol. 78 i-xii + 1-1079 (Kluwer Acad. Publishers, 1999).
15. Ganser, M. H. et al. A light and electron microscopical study on the resting cyst of the tintinnid *Schmidingerella* (Alveolata, Ciliophora) including a phylogeny-aware comparison. *Eur. J. Protistol.* **86**, 125922 (2022).
16. Smith, S. A. et al. Combined genome and transcriptome analyses of the ciliate *Schmidingerella arcuata* (Spirotrichea) reveal patterns of DNA elimination, scrambling, and inversion. *Genome Biol. Evol.* **12**, 1616–1622 (2020).
17. Zufall, R. A., McGrath, C. L., Muse, S. V. & Katz, L. A. Genome architecture drives protein evolution in ciliates. *Mol. Biol. Evol.* **23**, 1681–1687 (2006).
18. Maurer-Alcalá, X. X. & Nowacki, M. Evolutionary origins and impacts of genome architecture in ciliates. *Ann. N. Y. Acad. Sci.* **1447**, 110–118 (2019).

19. Smith, S. A., Santoferrara, L. F., Katz, L. A. & McManus, G. B. Genome architecture used to supplement species delineation in two cryptic marine ciliates. *Mol. Ecol. Resour.* **22**, 2880–2896 (2022).
20. Maurer-Alcalá, X. X., Cote-L'Heureux, A., Kosakovsky Pond, S. L. & Katz, L. A. Somatic genome architecture and molecular evolution are decoupled in "young" linage-specific gene families in ciliates. *PLoS ONE* **19**, e0291688 (2024).
21. Brookstein, O. et al. The natural material evolution and stage-wise assembly of silk along the silk gland. *bioRxiv*, 2024.2004.2016.589504 (2024).
22. Zeußel, L., Bargel, H., Holland, G. P. & Scheibel, T. Liquid–liquid phase separation of spider silk proteins. *Polymer J.* **57**, 831–843 (2025).
23. Brookstein, O. et al. Metal ions guide the production of silkworm silk fibers. *Nature Commun.* **15**, 6671 (2024).
24. Dittmar, W. in *Report of the Scientific Results of the Voyage of H.M.S. Challenger during the Years 1873-76. Physics and Chemistry - Volume 1* 1–251 + Plates I–III (Neill & Co., 1884).
25. Dolan, J. R. & Gallegos, C. L. Estuarine diversity of tintinnids (planktonic ciliates). *J. Plankton Res.* **23**, 1009–1027 (2001).
26. Dolan, J. R. & Pierce, R. W. in *The Biology and Ecology of Tintinnid Ciliates: Models for Marine Plankton* (eds Dolan, J. R. et al.) 214–243 (John Wiley & Sons, Ltd, 2013).
27. Kofoid, C. A. & Campbell, A. S. A conspectus of the marine and fresh-water Ciliata belonging to the suborder Tintinnoinea, with descriptions of new species principally from the Agassiz Expedition to the eastern tropical Pacific 1904-1905. *Univ. Calif. Publs Zool.* **34**, 1–403 (1929).
28. Kofoid, C. A. & Campbell, A. S. Reports on the scientific results of the expedition to the eastern tropical Pacific, in charge of Alexander Agassiz, by the U. S. Fish Commission Steamer "Albatross," from October, 1904, to March, 1905, Lieut.-Commander L. M. Garrett, U. S. N. Commanding. XXXVII. The Ciliata: The Tintinnoinea. *Bull. Mus. comp. Zool. Harv.* **84**, 1–473 + Plates I–XXXVI (1939).
29. Laval-Peuto, M. & Brownlee, D. C. Identification and systematics of the Tintinnina (Ciliophora): evaluation and suggestions for improvement. *Ann. Inst. océanogr., Paris* **62**, 69–84 (1986).
30. Agatha, S., Gruber, M. S., Bartel, H. & Weißenbacher, B. Somatic infraciliature in tintinnid ciliates (Alveolata, Ciliophora, Spirotricha): an ultrastructural comparison. *J. Eukaryot. Microbiol.* **69**, e12885 (2022).
31. Ganser, M. H., Bartel, H., Fedrizzi, M. & Agatha, S. A comparative ultrastructural study on the nanoscale extrusomes of tintinnids (Alveolata, Ciliophora, Spirotricha) and their phylogenetic significance. *Eur. J. Protistol.* **87**, 125953 (2023).
32. Gruber, M. S., Mühlthaler, A. & Agatha, S. Ultrastructural studies on a model tintinnid - *Schmidingerella meunieri* (Kofoid and Campbell, 1929) Agatha and Strüder-Kypke, 2012 (Ciliophora). I. Somatic kinetids with unique ultrastructure. *Acta Protozool.* **57**, 195–213 (2018).
33. Gruber, M. S., Weißenbacher, B. & Agatha, S. Ultrastructural studies on a model tintinnid - *Schmidingerella meunieri* (Kofoid & Campbell, 1929) Agatha & Strüder-Kypke, 2012 (Ciliophora). II. The oral apparatus. *J. Eukaryot. Microbiol.* **67**, 463–479 (2020).
34. Agatha, S. & Strüder-Kypke, M. C. Reconciling cladistic and genetic analyses in choreotrichid ciliates (Ciliophora, Spirotricha, Oligotrichea). *J. Eukaryot. Microbiol.* **59**, 325–350 (2012).
35. Montagnes, D. J. S. in *The Biology and Ecology of Tintinnid Ciliates: Models for Marine Plankton* (eds Dolan, J. R. et al.) 85–121 (John Wiley & Sons, Ltd, 2013).

36. Li, W. & Godzik, A. Cd-hit: a fast program for clustering and comparing large sets of protein or nucleotide sequences. *Bioinformatics* **22**, 1658–1659 (2006).
37. Fu, L., Niu, B., Zhu, Z., Wu, S. & Li, W. CD-HIT: accelerated for clustering the next-generation sequencing data. *Bioinformatics* **28**, 3150–3152 (2012).
38. Perez-Riverol, Y. et al. The PRIDE database at 20 years: 2025 update. *Nucl. Acids Res.* **53**, D543–D553 (2025).
39. Katz, L. A., Leleu, M., Ani, G., Gawron, R. & Cote-L'Heureux, A. Rethinking large-scale phylogenomics with EukPhylo v.1.0, a flexible toolkit to enable phylogeny-informed data curation and analyses of diverse eukaryotic lineages. *mBio* **16**, e01770–01725 (2025).
40. Buchfink, B., Reuter, K. & Drost, H.-G. Sensitive protein alignments at tree-of-life scale using DIAMOND. *Nat. Meth.* **18**, 366–368 (2021).
41. Cote-L'Heureux, A., Leleu, M., Ani, G., Gawron, R. & Katz, L. File_S2_1000taxa_ReadyToGo. Dataset. figshare https://doi.org/10.6084/m6089.figshare.25336129.v25336122 (2024).
42. Woehle, C. et al. Denitrification in foraminifera has an ancient origin and is complemented by associated bacteria. *Proc. Nat. Acad. Sci.* **119**, e2200198119 (2022).
43. Shazib, S. U. A. et al. Phylogenomic workflow for uncultivable microbial eukaryotes using single-cell RNA sequencing − a case study with planktonic ciliates (Ciliophora, Oligotrichea). *Mol. Phylogen. Evol.* **204**, 108239 (2025).
44. Frishman, D. & Argos, P. Knowledge-based protein secondary structure assignment. *Proteins: Struct., Funct., Bioinf.* **23**, 566–579 (1995).
45. Emenecker, R. J., Griffith, D. & Holehouse, A. S. Metapredict V2: an update to metapredict, a fast, accurate, and easy-to-use predictor of consensus disorder and structure. *bioRxiv*, 2022.2006.2006.494887 [Preprint] [bioRxiv] (2022).
