# Supplementary material for: Self-assembling proteins compose the chemically resistant shell biomaterial of planktonic tintinnid ciliates
Source: Nat Commun. 2026 Jun 13;17:7507. doi: 10.1038/s41467-026-74402-4 (PMC13408134; doi:10.1038/s41467-026-74402-4)
Supplement: Supplementary file 2 — Description of Additional Supplementary Files [file 41467_2026_74402_MOESM2_ESM.pdf]

Description of Additional Supplementary Files

File Name: Supplementary Movie 1
Description: Timelapse of shell-forming material secreted by a tintinnid ciliate specimen (*Schmidingerella*) on a glass slide observed by light microscopy.

File Name: Supplementary Data 1
Description: Single-cell transcriptome data of 20 individuals of the tintinnid *Schmidingerella* and their pooled data, called "reference transcriptome". The raw reads were quality-filtered (phred score ≥ 24) and trimmed to a minimum length of 100 base pairs with BBduk. Next, reads were processed with RiboDetector to distinguish ribosomal RNA (rRNA) sequences from non-ribosomal RNA sequences. The *de novo* transcriptome assemblies were generated for each cell, using SPAdes. N50 is the length of the shortest contig (or transcript) such that 50% of the total assembled sequence length is contained in contigs of that length or longer. L50 is the number of contigs (or transcripts) whose combined length makes up at least 50% of the total assembly length. Numbers of contigs in certain length ranges and total number of contigs.

File Name: Supplementary Data 2A
Description: Summarized Casanovo results on Tintinnidorin-1-alpha, beta, and gamma of the *Schmidingerella* shell from tandem-mass spectra analyses. Sample 1 contained about 690 shells that were incubated with proteinase K and subsequently with trypsin. Sample 2 contained about 490 shells that were incubated solely with proteinase K. Sample 3 contained about 500 shells that were incubated with elastase. Casanovo prediction results were summarized with the Stitch software. Length, number of amino acids; Score, sum of scores for all reads; Matches, total number of reads; Unique Score, sum of scores for all unique reads; Unique Matches, number of unique reads.

File Name: Supplementary Data 2B
Description: Tandem-mass spectra assessment with the proteomics data viewer PDV for manual inspection of predicted and measured peptide fragment masses of Tintinnidorin-1-alpha, beta, and gamma of the *Schmidingerella* shell. Tandem-mass spectrometry data are available via the PRIDE repository with the dataset identifier 10.6019/PXD070957. Peptide, amino acid sequence; Module, assigment to modules 1-6; Proteins, Tintinnidorin-1-alpha, beta, and gamma; Length, number of amino acids; Scan_ID, identification number of spectrum; Score, Stitch score for read; MS1, mass accuracy of precursor ion scan in parts per million (ppm); File, file which contains the spectrum.

File Name: Supplementary Data 3
Description: Relative gene expression of Tintinnidorin-1-alpha, beta, and gamma during the cell cycle in the tintinnid ciliate *Schmidingerella*. Relative gene expression is given in transcripts per million (TPM) for each variant and each of the 20 cells analyzed. Cell cycle stages: ED, early divider; LD, late divider; MD, middle divider; PD, postdivider; VLD, very late divider.

File Name: Supplementary Data 4A
Description: Subcellular localization and sorting signals predicted by DeepLoc for Tintinnidorin-1-alpha, beta, and gamma of the tintinnid ciliate *Schmidingerella*. DeepLoc distinguished between ten localizations, which are either membrane-associated or soluble. Upper table provides the threshold values for the different subcellular localizations and membrane types. Lower table lists the values for Tintinnidorin-1-alpha, beta, and gamma. The probability for Tintinnidorin is above the thresholds for proteins that are not embedded in a membrane but soluble and extracellular (marked by green). Respective signal peptides were detected (Supplementary Table 4B).

File Name: Supplementary Data 4B
Description: Signal peptides at the N-terminal regions of the tintinnid shell proteins Tintinnidorin-1-alpha, beta, and gamma of *Schmidingerella*. SignalP results for eukaryotes suggest with highest probabilities that the Tintinnidorin proteins are targeted to the secretory pathway by signal peptides (SP), while the proteins' probability for no signal peptides (OTHER) is very low. The cleavage position (CS) marks the site (as number of the bordering amino acids in the sequence) where the signal peptide is cut from the non-signal peptide region (OTHER); further, the probability values (Pr) for this site are given.

File Name: Supplementary Data 5
Description: Results of the OrthoFinder analyses based on single-cell transcriptomes of 20 *Schmidingerella* cells, the *Schmidingerella* reference transcriptome, 46 single-cell transcriptomes of other tintinnid ciliates, and 232 genomes and transcriptomes from a wide variety of bacteria, archaea, and eukaryotes. Partial and complete Tintinnidorin protein sequences were exclusively detected in single-cell transcriptomes of tintinnid ciliates and assigned to a single hierarchical orthogroup.

File Name: Supplementary Data 6
Description: Currently known Tintinnidorin proteins and their structurally most similar matches in the Protein Data Bank (PDB) or UniProt. From the five models generated by AlphaFold2 for each protein sequence, the respective top-ranked models

were used as queries for exhaustive structure searches in PDB and UniProt. Target, hit with the highest structure similarity as characterized by the TopMatch structure similarity score. Target IDs starting with "AF-" denote AlphaFold predictions for UniProt sequences available from the AlphaFold Protein Structure Database (https://alphafold.com); remaining target IDs denote experimentally determined structures available from PDB (https://rcsb.org; <PDB code>_<chain id>). TopMatch structure similarity score, can range from 0 to the length of the structure alignment (i.e., to the length of the shorter of the two structures compared); details in Wiederstein & Sippl 2020. TopMatch-web: pairwise matching of large assemblies of protein and nucleic acid chains in 3D. Nucl. Acids Res. 48: W31-W35. https://doi.org/10.1093/nar/gkaa366. Query coverage, 100 x *L/Qn*, where *L* is the alignment length and *Qn* is the number of residues in the query structure. Sequence identity, percentage of identical residues in structure-based sequence alignment.

File Name: Supplementary Data 7
Description: Currently known Tintinnidorin protein sequences and their metrics. Screening the Tara Oceans database and the North Pacific Eukaryotic Gene Catalog yielded a considerable diversity of 72 Tintinnidorin proteins beyond our single-cell dataset of Tintinnidorin-1 from Schmidingerella and Tintinnidorin-2 from Tintinnopsis cylindrica. The very high PICNIC scores suggest high propensities for condensate formation by Tintinnidorin proteins. Fractions of amino acids in the shell-forming proteins of tintinnid ciliates. A, alanine; G, glycine; V, valine; L, leucine; I, isoleucine; M, methionine; P, proline; F, phenylalanine; W, tryptophan; Y, tyrosine; S, serine; Q, glutamine; T, threonine; N, asparagine; D, aspartic acid; E, glutamic acid; K, lysine; R, arginine; H, histidine; C, cysteine. 1, Taxonomic annotation of Tara unigenes based on the Lowest Common Ancestor approach (Carradec et al. 2018. A global ocean atlas of eukaryotic genes. Nat. Commun. 9: 373.) https://doi.org/10.1038/s41467-017-02342-1).

File Name: Supplementary Data 8
Description: Comparison of amino acid compositions. Amino acid fractions in the Tintinnidorin-1-alpha, beta, and gamma proteins of *Schmidingerella*, average fractions of the *Schmidingerella* proteome, the Swiss-Prot database, and the 78 full-length Tintinnidorin protein sequences, and fractions of selected animal biomaterial proteins (spider major ampullate spidroins 1 and 2; silkworm fibroin heavy and light chains; mussel foot protein). A, alanine; G, glycine; V, valine; L, leucine; I, isoleucine; M, methionine; P, proline; F, phenylalanine; W, tryptophan; Y, tyrosine; S, serine; Q, glutamine; T, threonine; N, asparagine; D, aspartic acid; E, glutamic acid; K, lysine; R, arginine; H, histidine; C, cysteine.

File Name: Supplementary Data 9
Description: Comparison of linker lengths in Tintinnidorin proteins. The numbers of amino acids in the linkers 1-5 of the Tintinnidorins from the single-cell dataset comprising Tintinnidorin-1 from *Schmidingerella* and Tintinnidorin-2 from *Tintinnopsis cylindrica* and those sequences detected in the Tara Oceans database and the North Pacific Eukaryotic Gene Catalog.

File Name: Supplementary Data 10
Description: Between-group comparisons of amino acid fractions (modules vs. linkers). Differences in central tendency between groups were assessed, using the Wilcoxon rank-sum test (two-sided); homogeneity of variances was evaluated with Levene's test (two-sided). A, alanine; G, glycine; V, valine; L, leucine; I, isoleucine; M, methionine; P, proline; F, phenylalanine; W, tryptophan; Y, tyrosine; S, serine; Q, glutamine; T, threonine; N, asparagine; D, aspartic acid; E, glutamic acid; K, lysine; R, arginine; H, histidine; C, cysteine. Wilcoxon_p, Levene_p, p-values; Wilcoxon_FDR, Levene_FDR, adjusted p-values, using the Benjamini-Hochberg false discovery rate.

File Name: Supplementary Data 11
Description: Comparison of amino acid compositions in the module regions of Tintinnidorin protein sequences. Amino acid fractions in Tintinnidorins from the single-cell dataset comprising Tintinnidorin-1 from *Schmidingerella* and Tintinnidorin-2 from *Tintinnopsis cylindrica* and those sequences detected in the Tara Oceans database and the North Pacific Eukaryotic Gene Catalog. A, alanine; G, glycine; V, valine; L, leucine; I, isoleucine; M, methionine; P, proline; F, phenylalanine; W, tryptophan; Y, tyrosine; S, serine; Q, glutamine; T, threonine; N, asparagine; D, aspartic acid; E, glutamic acid; K, lysine; R, arginine; H, histidine; C, cysteine.

File Name: Supplementary Data 12
Description: Comparison of amino acid compositions in the linker regions of Tintinnidorin protein sequences. Amino acid fractions in Tintinnidorins from the single-cell dataset comprising Tintinnidorin-1 of *Schmidingerella* and Tintinnidorin-2 of *Tintinnopsis cylindrica* and those sequences detected in the Tara Oceans database and the North Pacific Eukaryotic Gene Catalog. A, alanine; G, glycine; V, valine; L, leucine; I, isoleucine; M, methionine; P, proline; F, phenylalanine; W, tryptophan; Y, tyrosine; S, serine; Q, glutamine; T, threonine; N, asparagine; D, aspartic acid; E, glutamic acid; K, lysine; R, arginine; H, histidine; C, cysteine.

File Name: Supplementary Data 13
Description: Pairwise similarity values of the modules 1-6 of Tintinnidorin-1-alpha, beta, and gamma protein sequences from *Schmidingerella*. High similarity values are found among odd-numbered (M1, 3, 5) and even-numbered (M2, 4, 6) modules, respectively.

File Name: Supplementary Data 14
Description: Coordinates of sampling sites of the Tara Oceans database and the North Pacific Eukaryotic Gene Catalog at which Tintinnidorin protein sequences were detected. Positive latitude values indicate a location in the Northern Hemisphere, while negative values indicate a location in the Southern Hemisphere. Likewise, positive longitudinal values indicate a location east of the Prime Meridian, while negative values indicate a location west of the Prime Meridian.

File Name: Supplementary Data 15
Description: Nucleotide and amino acid sequences of the 78 full-length Tintinnidorin proteins.

File Name: Supplementary Data 16
Description: Predicted AlphaFold2 structure models of the 78 full-length Tintinnidorin proteins.
