# Supplementary material for: Self-assembling proteins compose the chemically resistant shell biomaterial of planktonic tintinnid ciliates
Source: Nat Commun. 2026 Jun 13;17:7507. doi: 10.1038/s41467-026-74402-4 (PMC13408134; doi:10.1038/s41467-026-74402-4)
Supplement: Supplementary file 8 — Transparent Peer Review file [file 41467_2026_74402_MOESM8_ESM.pdf]

nature portfolio

Peer Review File

Self-assembling proteins compose the chemically resistant shell biomaterial of planktonic tintinnid ciliates

Corresponding Author: Dr Maximilian Ganser

This file contains all reviewer reports in order by version, followed by all author rebuttals in order by version.

Version 0:

Reviewer comments:

Reviewer #1

(Remarks to the Author)
This manuscript is of considerable significance in that it identifies a novel proteinaceous component through analysis of the lorica of a tintinnid species, a unicellular marine planktonic ciliate characterized by its distinctive loricate structure. The discovery of a new proteinaceous substance derived from the lorica is scientifically meaningful. If followed by detailed biochemical characterization and material synthesis studies, this novel protein may hold promising potential for future application as a functional biomaterial.

The following points require clarification and further elaboration:
1. The authors should more clearly emphasize the structural and functional significance of the lorica that enables tintinnids to achieve broad spatiotemporal distribution in marine environments. In particular, the specific role of the newly identified lorica component in supporting the survival and ecological success of tintinnids should be more explicitly addressed.
2. Since a sufficient supply of experimental material is essential for the analyses conducted, detailed and precise information regarding long-term stable culture conditions should be provided. This should include parameters such as light intensity, prey concentration, and whether axenic or non-axenic conditions were maintained. Given that sustainable laboratory culture of tintinnids and other ciliates is generally considered challenging, clarification regarding the stability and reproducibility of the culture system is strongly recommended.
3. Although the strain is described as a monoclonal culture, the manuscript refers to it as Schmidingerella sp. The authors should clarify why it was not identified at the species level (e.g., as S. arcuata, if applicable) and provide supporting morphological or molecular evidence for the taxonomic designation.
4. In Fig. 3, the species name should be corrected to Schmidingerella taraikaensis (please verify spelling and authority).
5. The manuscript describes that the promodium formed in the mother cell results in the posterior daughter cell inheriting the parental lorica, whereas the anterior cell secretes and constructs a new lorica. If so, the anterior cell retains its primary locomotory apparatus (ciliary fans) across successive generations. This raises a biological question as to whether aging ciliary structures maintain full functional integrity, or whether structural renewal occurs. Further discussion on this point would strengthen the biological interpretation.
6. Some images (e.g., Fig. 1) appear to have been previously published in other papers but are used without citation. All previously published materials must clearly indicate their original source and, where applicable, include appropriate permission statements. Only data generated exclusively for this manuscript should be presented without prior citation.

Reviewer #2

(Remarks to the Author)
The manuscript describes the identification of new structural proteins forming the shell of tintinnid ciliates. The study is highly interesting, comprehensive, and clearly presented, and it will likely appeal to the broad readership of Nature Communications. I recommend acceptance of the manuscript for publication. My comments below are relatively minor and are intended to further improve the clarity of the paper:
1. Title: The current title is somewhat broad and non-specific, which is generally more typical of review articles than original research papers. I encourage the authors to consider a more specific title that better reflects the unique findings of this study.

2. Shell isolation methodology: Could the authors briefly clarify in the main text how the shell was separated from the rest of

the organismal body? This step may not be obvious to readers outside the immediate field and a short explanation would improve accessibility.
3. Role of LLPS (Line 309): The authors mention liquidlliquid phase separation (LLPS). It would be helpful if they could elaborate on its proposed role in the assembly process of tintinnidroin proteins. Do the authors envision LLPS as a storage or pre-assembly mechanism similar to that described in Bombyx mori silk? Clarifying this point would strengthen the mechanistic discussion. I also suggest including relevant references in this context:
https://www.biorxiv.org/content/10.1101/2024.04.16.589504v1.abstract
4. Role of ionic composition (Lines 336–355): The authors discuss the influence of the external environment, particularly ionic composition. It would be interesting to explore, perhaps in future studies, how variations in ionic conditions affect tintinnidroin self-assembly and to compare these effects with those described for well characterized silk systems (see example: https://www.nature.com/articles/s41467-024-50879-9). Briefly acknowledging this comparison in the discussion could broaden the impact of the work.
5. Section title ("Synthesis: …"): The section titled "Synthesis: …" does not appear to describe a chemical or biochemical synthesis process. In my view, the section title should be reconsidered and renamed to more accurately reflect its content.

Reviewer #3

(Remarks to the Author)
Maximilian et al. reports the discovery and characterization of Tintinnidorin, a previously unknown family of structural proteins that form the shell of tintinnid Schmidingerella. The work is methodologically impressive, combining single-cell transcriptomics, proteomics, structural bioinformatics, and metatranscriptomic and genomic datasets. The study identifies a novel protein architecture with rich aromatic residue composition, and provides plausible links between sequence, structure, and material properties such as self-assembly and resilience. The study opens a new avenue in biomaterials research and adds a valuable perspective from microbial eukaryotes, a group often overlooked in this field. However, while the in silico and descriptive evidence is compelling, the manuscript would benefit from experimental validation to strengthen some of the mechanistic claims. I have several suggestions for revision.
1. The current evidence for properties such as tensile strength, adhesion, or self-assembly kinetics is based on indirect or predictive data. I would encourage the authors to consider recombinant expression of Tintinnidorin variants, or other simple and effective experimental validation of material properties. This would substantially strengthen the conclusions regarding the biomaterial potential of these proteins.
2. The linkers are proposed to be intrinsically disordered and critical for phase separation, but they are poorly covered in the mass spectrometry data. The authors mention possible reasons (detection limits or crosslinking). A more detailed discussion —or ideally, additional experiments using alternative digestion protocols—would help clarify whether this is a technical limitation or reflects a genuine structural feature.
3. Overinterpretation of some claims. Two examples stood out: the statement that Tintinnidorin proteins "maintain self-assembly capacity across a wide range of salinities" is inferred from species distribution but not directly tested. Similarly, the term "wet adhesion" is used without supporting adhesion assays. I recommend tempering these claims or adding appropriate caveats.
Minor points:
1. Some figures are dense and difficult to read, particularly Fig. 4 and Extended Data Fig. 3. Simplifying these or splitting them into multiple panels would improve accessibility.
2. In all result figures, placing the label of each sub-figure in the top-left corner would be more consistent with standard reading habits.
3. A glossary of specialist terms (e.g., LLPS, pLDDT) would be useful for readers outside structural biology or biomaterials.
4. The discussion of evolutionary mechanisms (e.g., alternative processing, gene conversion) is brief. Expanding this section would strengthen the evolutionary narrative.

Version 1:

Reviewer comments:

Reviewer #1

(Remarks to the Author)
The authors have responded diligently and appropriately to all comments and have revised the manuscript accordingly. In addition, they have provided detailed explanations in the supplementary notes, which have been carefully reviewed.

Reviewer #2

(Remarks to the Author)
Authors addressed my comments. I recommend to accept this manuscript to publication.

Reviewer #3

(Remarks to the Author)
I have reviewed the revised manuscript and the authors' response to the previous round of comments. The authors have carefully addressed the concerns raised, primarily by revising the main text, improving figure presentation, clarifying ambiguous statements, and expanding the discussion where needed. For the suggestions that were not fully implementable

(e.g., certain experimental validations), the authors have provided reasonable explanations or acknowledged them as future work, which I find acceptable.

The manuscript is now clearer and better organised. I have no further technical or conceptual concerns. The work remains novel, methodologically sound, and of broad interest. I am pleased to recommend acceptance of the manuscript in its current form.

Open Access This Peer Review File is licensed under a Creative Commons Attribution 4.0 International License, which permits use, sharing, adaptation, distribution and reproduction in any medium or format, as long as you give appropriate credit to the original author(s) and the source, provide a link to the Creative Commons license, and indicate if changes were made.
In cases where reviewers are anonymous, credit should be given to 'Anonymous Referee' and the source.
The images or other third party material in this Peer Review File are included in the article's Creative Commons license, unless indicated otherwise in a credit line to the material. If material is not included in the article's Creative Commons license and your intended use is not permitted by statutory regulation or exceeds the permitted use, you will need to obtain permission directly from the copyright holder.
To view a copy of this license, visit https://creativecommons.org/licenses/by/4.0/

A point-by-point reply to the comments of the reviewers

We thank the three reviewers for their constructive comments and helpful suggestions. Below, we provide detailed point-by-point responses to each remark. To maintain the current narrative of the manuscript, we have limited the additional information included in the main text to one or two sentences where appropriate, while providing more detailed explanations in the Supplementary Notes. For literature references, please, consult the Supplementary Information.

Reviewer #1 (Remarks to the Author):

This manuscript is of considerable significance in that it identifies a novel proteinaceous component through analysis of the lorica of a tintinnid species, a unicellular marine planktonic ciliate characterized by its distinctive loricate structure. The discovery of a new proteinaceous substance derived from the lorica is scientifically meaningful. If followed by detailed biochemical characterization and material synthesis studies, this novel protein may hold promising potential for future application as a functional biomaterial.

The following points require clarification and further elaboration:
1. The authors should more clearly emphasize the structural and functional significance of the lorica that enables tintinnids to achieve broad spatiotemporal distribution in marine environments. In particular, the specific role of the newly identified lorica component in supporting the survival and ecological success of tintinnids should be more explicitly addressed.

ANSWER:

To give readers a sense of the literature, we have added lines 52-54: "The proposed adaptive advantages of the shells for tintinnid ciliates are primarily associated with protection and efficient filter feeding (Supplementary Note 1)."

We have also created a new section "Function of tintinnid shells" (Supplementary Note 1) to the Supplementary information, which states:

Several advantages of tintinnid loricae accounting for both their comparatively high species richness and their global, predominantly marine distribution have been proposed, most of which relate to protection and feeding; rarely, the properties of the shell material itself beyond its ability to self-assemble provide advantages for the survival and the fitness of the tintinnid specimens. Although the construction and transport of the lorica impose additional energetic costs, its weight is frequently advantageous. From a protective perspective, the lorica may operate through multiple mechanisms. In contrast to previous speculations (Armstrong & Brasier 2005), the present study provides the first evidence for the shells' partial shielding against ultraviolet radiation due to the comparatively high amounts of amino acids with aromatic residues and an autofluorescence after excitation at these wavelengths. Further, the shell may reduce the exudation of chemical cues detectable by predators (Capriulo et al. 1982). Loricae also facilitate rapid sinking of a contracted tintinnid out of a predator's attack trajectory (Capriulo et al. 1982) and increase the likelihood that a tintinnid contracted into its lorica will be released by predatory copepods (Echevarria et al. 2016). Nevertheless, these protective mechanisms are not completely effective. Undamaged loricae recovered from copepod fecal pellets demonstrate that tintinnid cells are digested like other ingested prey despite the high resistance of their loricae (Turner 1984) supposedly caused by the extracellular formation of beta-sheets by the Tintinnidorin proteins. A second suite of

advantages concerns feeding. Experimental studies suggest that the weight of the lorica, in combination with the apical ring of (collar) membranelles, stabilizes the organism with its apical end oriented upward. This creates a predominantly upward swimming direction that facilitates the exploitation of phytoplankton-rich surface layers (Jonsson 1989). Furthermore, advanced hydrodynamic experiments indicate that the lorica increases drag, thereby strengthening the feeding current and enhancing the encounter rate with food particles (Jiang & Buskey 2024). These advantages might in concert have promoted the evolution of the tintinnid ciliates.

2. Since a sufficient supply of experimental material is essential for the analyses conducted, detailed and precise information regarding long-term stable culture conditions should be provided. This should include parameters such as light intensity, prey concentration, and whether axenic or non-axenic conditions were maintained. Given that sustainable laboratory culture of tintinnids and other ciliates is generally considered challenging, clarification regarding the stability and reproducibility of the culture system is strongly recommended.

ANSWER:

We provide details on the conditions that were used to establish stable cultures and have added a reference to the respective Methods section "Sampling and cultivation" in lines 437-438: "Details on the conditions used to establish cultures of *Schmidingerella* in the Strom lab are provided in Supplementary Method 2."

Supplementary Method 2 "Cultivation of tintinnid ciliates" states:

The autecology of most tintinnid species, including their preferred prey, remains poorly understood. Consequently, establishing laboratory cultures is largely a process of trial and error and requires continuous access to fresh material. We repeatedly obtained monoclonal strains from the Strom laboratory. Increasing strain numbers indicated that new cultures were periodically initiated by isolating single *Schmidingerella* cells from field samples.

In the Strom laboratory, non-axenic cultures were maintained in 500-ml flasks containing 300 ml of ciliate medium, which were inoculated twice weekly and provisioned with 4 ml of the dinoflagellate *Heterocapsa triquetra*, half a Pasteur pipette of the prasinophyte *Mantoniella squamata*, and the haptophyte *Isochrysis galbana* (pers. commun.). The culture flasks were incubated at 15 °C with a 12:12 diel cycle of low light (about 20 to 30 µmol photons/m²/sec). The cultures were non-axenic and occasionally contained additional, unidentified flagellates at low abundances. *Heterocapsa triquetra* appeared essential for sustaining growth of our strains, and both this species and *I. galbana* have been used frequently in the cultivation of marine planktonic tintinnids (Montagnes 2013).

We continued to culture the strains following the recommendations of the Strom laboratory and using *Heterocapsa triquetra* as the main food supplemented with *Isochrysis galbana*, provided as cultures. Under these conditions, *Schmidingerella* cultures could be maintained for up to six months. In several strains, conjugation was observed shortly before a pronounced decline and eventual collapse of the cultures. Depletion of prey organisms led to heavy bacterial infestation of the tintinnids, followed by culture termination. Application of these culture conditions to other tintinnid species is constrained by the limited knowledge of the species-specific autecological requirements.

3. Although the strain is described as a monoclonal culture, the manuscript refers to it as Schmidingerella sp. The authors should clarify why it was not identified at the species level

(e.g., as S. arcuata, if applicable) and provide supporting morphological or molecular evidence for the taxonomic designation.

ANSWER:

The explanation why we refrain from assigning our *Schmidingerella* specimens to a species is now provided in Supplementary Method 1 “Species identification in *Schmidingerella*”:

Tintinnid taxonomy is largely based on lorica characteristics, including shape, structure, and size. Most original species descriptions were published roughly eighty to one hundred years ago (Kofoid & Campbell, 1929, 1939), typically relying on very limited material, often only one to five specimens. Consequently, the natural range of morphological variability was not captured. In many cases, only lorica length was reported, which is problematic because length is now known to vary substantially among conspecific individuals, whereas the opening diameter is comparatively constant (Laval-Peuto & Brownlee 1982) and thus a more reliable diagnostic feature. Accordingly, this character must be inferred from line drawings, using given scale bars or magnifications, or from the ratio of lorica length to opening diameter, both very imprecise approaches.

In the genus *Schmidingerella* (formerly included within *Favella*), inferred lorica opening diameters span an exceptionally wide range in some species, whereas in most species only single values have been deduced. Because of substantial overlap in opening diameters and the overall similarity of lorica morphologies, the taxonomy of this tintinnid genus requires a comprehensive integrative revision. Such a revision should include analyses of a substantial number of monoclonal strains from different localities, examining lorica morphology and ultrastructure, cell features, particularly the ciliary pattern, and multiple genetic markers. Only with these data will it be possible to clearly distinguish intraspecific variability from interspecific similarity.

Previously, *Schmidingerella* specimens collected in the Northeast Pacific were generally assigned to *S. meunieri* (Agatha & Bartel 2022; Agatha et al. 2022; Ganser et al. 2022, 2023; Gruber et al. 2018, 2020; Smith et al. 2022), whereas those from the Northwest Atlantic were identified as *S. arcuata* (Agatha & Strüder-Kypke 2012; Smith et al. 2022). Barcoded specimens from these regions exhibit minor differences in ribosomal marker gene sequences and show overlapping ranges of lorica characteristics. The variation in lorica morphology among the Northeast Pacific strains obtained from the Strom laboratory suggests that more than one species may occur in the Northeast Pacific. The marker gene sequences of the strain analysed in the present study match those of specimens previously identified as *S. meunieri* solely based on their Northeast Pacific origin (Smith et al. 2022). Given the unresolved and problematic taxonomic situation, we refrain from assigning our specimens to a species.

4. In Fig. 3, the species name should be corrected to Schmidingerella taraikaensis (please verify spelling and authority).

ANSWER:

In Figure 3, we corrected the misspelled species name to *Schmidingerella taraikaensis*. The species has been described by Hada (1932) under the name *Favella taraikaensis* and had been transferred by Agatha & Strüder-Kypke (2012) to the genus *Schmidingerella*, becoming *Schmidingerella taraikaensis* (Hada, 1932) Agatha & Strüder-Kypke, 2012.

5. The manuscript describes that the promodium formed in the mother cell results in the posterior daughter cell inheriting the parental lorica, whereas the anterior cell secretes and

constructs a new lorica. If so, the anterior cell retains its primary locomotory apparatus (ciliary fans) across successive generations. This raises a biological question as to whether aging ciliary structures maintain full functional integrity, or whether structural renewal occurs. Further discussion on this point would strengthen the biological interpretation.

ANSWER:

We provide a brief discussion in Supplementary Note 3 “Reorganization of membranellar zone tintinnids”, with a reference in line 105 “ciliary fans (membranelles; Supplementary Note 3)”:

Renewal of the oral and locomotory structures (membranelles; ciliary fans) in tintinnid ciliates represents an intriguing biological question. Observations of live cells are limited by the difficulty of tracking a single proter across multiple cell divisions. In protargol-stained specimens showing the nuclear apparatus and basal bodies, no indications of membranelle renewal or reorganization have been detected (Agatha et al. 2025). In several hypotrich ciliates, taxa relatively closely related to tintinnids and possessing a homologous zone of membranelles, partial reorganization of the oral ciliature during cell division and conjugation (reciprocal fertilization) has been documented (Agatha & Foissner 2009; Berger 1999). Because the ciliature is resorbed during encystment in tintinnids and several hypotrichs (Ganser et al. 2022), excystment necessarily involves its reconstruction and therefore renewal. Additional complexity is introduced by the capacity of some hypotrich ciliates for physiological reorganization in non-dividing cells, triggered by altered nutritional conditions, as well as post-traumatic regeneration following mechanical damage (Berger 1999). However, none of these processes is directly associated with lorica formation.

6. Some images (e.g., Fig. 1) appear to have been previously published in other papers but are used without citation. All previously published materials must clearly indicate their original source and, where applicable, include appropriate permission statements. Only data generated exclusively for this manuscript should be presented without prior citation.

ANSWER:

Images are either original, owned by the authors, or reproduced with permission obtained through the Copyright Clearance Center (Supplementary Note 8). (also added to lines 75-76)

Figure 1b: Lorica image published in Agatha, S., Laval-Peuto, M. & Simon, P. in *The Biology and Ecology of Tintinnid Ciliates: Models for Marine Plankton* (eds Dolan, J. R. et al.) 17–41 (John Wiley & Sons, Ltd, 2013).

Figure 1c: Lorica image published in Agatha, S. & Bartel, H. A comparative ultrastructural study of tintinnid loricae (Alveolata, Ciliophora, Spirotricha) and a hypothesis on their evolution. J. Eukaryot. Microbiol. 69, e12877 (2022). https://doi.org/10.1111/jeu.12877

Figure 1e: Lorica image published in Agatha, S. A light and scanning electron microscopic study of the closing apparatus in tintinnid ciliates (Ciliophora, Spirotricha, Tintinnina): a forgotten synapomorphy. J. Eukaryot. Microbiol. 57, 297–307 (2010). https://doi.org/10.1111/j.1550-7408.2010.00490.x

Figure 1e: Image of lorica wall section published in Laval-Peuto, M. in *Traité de Zoologie. Anatomie, systématique, biologie. 2. Infusoires ciliés. 2. Systématique* (ed P. de Puytorac) 181–219 (Masson, 1994).

Figure 1f: Lorica image published in Agatha, S. & Bartel, H. A comparative ultrastructural study of tintinnid loricae (Alveolata, Ciliophora, Spirotricha) and a hypothesis on their

evolution. J. Eukaryot. Microbiol. 69, e12877 (2022). https://doi.org/10.1111/jeu.12877

Reviewer #2 (Remarks to the Author):

The manuscript describes the identification of new structural proteins forming the shell of tintinnid ciliates. The study is highly interesting, comprehensive, and clearly presented, and it will likely appeal to the broad readership of Nature Communications. I recommend acceptance of the manuscript for publication. My comments below are relatively minor and are intended to further improve the clarity of the paper:

1. Title: The current title is somewhat broad and non-specific, which is generally more typical of review articles than original research papers. I encourage the authors to consider a more specific title that better reflects the unique findings of this study.

ANSWER:

The author guidelines of Nature Communications restrict the title length to 15 words.

- Current title: A new class of proteinaceous biomaterial with unprecedented properties in unicellular eukaryotes

We suggest this more specific title naming two main characteristics of the tintinnid shell biomaterial:

- Self-assembling proteins compose the highly resistant shell biomaterial of planktonic tintinnid ciliates

2. Shell isolation methodology: Could the authors briefly clarify in the main text how the shell was separated from the rest of the organismal body? This step may not be obvious to readers outside the immediate field and a short explanation would improve accessibility.

ANSWER:

We explain the shell isolation methodology in lines 521-523 of the Methods (“Preparation of shells”): “Empty and intact shells of Schmidingerella specimens (SPMC 176) were continuously collected from the culture material, thoroughly washed in ddH2O to remove salt, and air-dried in 1.5 mL Eppendorf tubes, using a desiccator (Supplementary Method 4).”

More details are now also provided in Supplementary Method 4 (“Isolation of tintinnid shells for proteomics”):

In the 6-well plates used for culturing the monoclonal *Schmidingerella* tintinnids, empty loricae sedimented after cell death and gradually accumulated at the bottom of the wells. Intact, empty, and optically clean shells (verified by inspection at 115× magnification) were collected with a finely drawn pipette approximately every two days to prevent bacterial colonization. The isolated shells were subsequently transferred through five to six rinsing steps in ddH□O to remove residual contaminants and sea salts. After washing, the shells were placed in 1.5-ml Eppendorf caps with minimal water. With the lids left open, the caps were positioned in a desiccator to allow the shells to air-dry. Due to the labour-intensive nature of the procedure and the restricted number of shells meeting the required specifications, the about 1,700 shells selected for proteomics represent a significant proportion of the available material.

3. Role of LLPS (Line 309): The authors mention liquidlliquid phase separation (LLPS). It would be helpful if they could elaborate on its proposed role in the assembly process of tintinnidroin proteins. Do the authors envision LLPS as a storage or pre-assembly

mechanism similar to that described in Bombyx mori silk? Clarifying this point would strengthen the mechanistic discussion. I also suggest including relevant references in this context: https://www.biorxiv.org/content/10.1101/2024.04.16.589504v1.abstract

ANSWER:

We have compared the very well-studied mechanisms regarding the role of LLPS in *Bombyx mori* with our findings and discuss the potential similarities and differences in Supplementary Note 6 ("Storage of structural proteins in tintinnids compared to animal silk glands"):

Our transmission electron microscopic analyses of the maturation process of shell-forming material granules composed of the tintinnid shell protein Tintinnidorin revealed distinct structural transitions (Ganser et al. 2025). We interpret these transitions as successive stages of a liquid–liquid phase separation (LLPS) process. The material in the mature granules appears to be stored in a highly compact state minimizing the volume while preventing unintended interactions between the proteins. These findings indicate that LLPS plays an important role for the storage of structural proteins in completely different biological systems, i.e., in tintinnid ciliates and also in the silkworm *Bombyx mori* (Brookstein et al. 2024, bioRxiv) as well as in spiders (Zeußel et al. 2025). A key distinction among these systems is the site of LLPS: in tintinnids, LLPS occurs within intracellular secretory vesicles, whereas in silkworms and spiders it takes place in the lumen of the silk glands. Conditions also differ markedly regarding pH. In tintinnids, LLPS proceeds at low pH (≤ 5) within the secretory vesicles, followed by a rapid increase in pH when the material is released into the (sea-)water after cell division. In contrast, the pH within the silk gland lumina decreases from approximately 8–7 in the proximal region to 6–4 distally. The main difference is that a controlled pre-assembly of silk proteins takes places before the fibres are formed by the spinning organ, whereas Tintinnidorin proteins start to self-assemble immediately when getting in contact with the surrounding (sea-) water.

Manuscript changes comprise lines 316-321: "Structural transitions of the shell-forming material during intracellular maturation revealed by transmission electron microscopy[17] are probably the result of phase separation (Fig. 2). LLPS is strongly associated with storage and self-assembly processes in many protein-based materials of animals and pathogenic amyloids in humans[1,27,46,47]. These findings indicate that LLPS plays an important role for structural proteins in completely different biological systems (Supplementary Note 6)."

4. Role of ionic composition (Lines 336–355): The authors discuss the influence of the external environment, particularly ionic composition. It would be interesting to explore, perhaps in future studies, how variations in ionic conditions affect tintinnidroin self-assembly and to compare these effects with those described for well characterized silk systems (see example: https://www.nature.com/articles/s41467-024-50879-9). Briefly acknowledging this comparison in the discussion could broaden the impact of the work.

ANSWER:

We rephrased the aspect about the influence of extracellular conditions on self-assembly in lines 347-353: "In comparison to these animals, tintinnid ciliates cannot actively control the extracellular physicochemical environment during shell formation as the shell-forming material is rapidly secreted into the surrounding (sea-)water. Nevertheless, family and genus-specific shell characteristics are consistent across widely differing environmental conditions. In some genera, species have even been recorded from seawater, brackish water, and freshwater. How Tintinnidorin proteins reliably self-assemble despite such contrasting conditions remains an open question (Supplementary Note 7)."

And discuss details in Supplementary Note 7 (“Influence of ionic composition on material assembly”):

The environmental conditions during Tintinnidorin self-assembly in seawater are hardly comparable with those during protein pre-assembly in the lumen of silk glands where the silkworms regulate the pH and the metal ion concentrations (Brookstein et al. 2024). In our study system, the shell-forming material is exposed to low pH values (≤ 5) in the (intracellular) secretory vesicles. With its secretion, the granular material abruptly gets in contact with seawater having a pH of around 8.1 and consisting of a broad spectrum of ions. In the seawater, the major ions show constant ratios globally in the salinity range of about 33-37 ‰ (Forchhammer's Principle; Dittmar 1884). In brackish waters, the ion composition fluctuates due to freshwater influence and biological processes. The total salinity concentration affects the distribution ranges of tintinnid species, although euryhaline estuarine species are occasionally recorded (Dolan and Gallegos 2001). Generally, genera exhibit a markedly greater tolerance to salinity gradients (Dolan and Pierce 2013), some occurring across regions with elevated salinity levels (e.g., about 40‰ in the Red Sea) to low salinity brackish environments (e.g., the Baltic Sea). Extreme examples are the genera *Codonella*, *Stenosemella*, and *Tintinnidium*, which even occur in freshwater lakes, still forming the genus-specific shells. In the present study, we have identified 78 full-length Tintinnidorin proteins from single-cell and metatranscriptome data. Congruently with microscopic data, these findings indicate a global distribution of tintinnids. If Tintinnidorin self-assembly depends on ions, they are thus apparently universally available in sufficient concentrations or can be replaced by equivalent ions. Future studies have to answer the question of how Tintinnidorin proteins manage self-assembly under such contrasting conditions.

5. Section title (“Synthesis: …”): The section titled “Synthesis: …” does not appear to describe a chemical or biochemical synthesis process. In my view, the section title should be reconsidered and renamed to more accurately reflect its content.

ANSWER:

To accurately reflect the content of the section, we renamed the title to “Potential of Tintinnidorin as a novel model system for biomaterial research”

Reviewer #3 (Remarks to the Author):

Maximilian et al. reports the discovery and characterization of Tintinnidorin, a previously unknown family of structural proteins that form the shell of tintinnid Schmidingerella. The work is methodologically impressive, combining single-cell transcriptomics, proteomics, structural bioinformatics, and metatranscriptomic and genomic datasets. The study identifies a novel protein architecture with rich aromatic residue composition, and provides plausible links between sequence, structure, and material properties such as self-assembly and resilience. The study opens a new avenue in biomaterials research and adds a valuable perspective from microbial eukaryotes, a group often overlooked in this field. However, while the in silico and descriptive evidence is compelling, the manuscript would benefit from experimental validation to strengthen some of the mechanistic claims. I have several suggestions for revision.

1. The current evidence for properties such as tensile strength, adhesion, or self-assembly kinetics is based on indirect or predictive data. I would encourage the authors to consider recombinant expression of Tintinnidorin variants, or other simple and effective experimental validation of material properties. This would substantially strengthen the conclusions regarding the biomaterial potential of these proteins.

ANSWER:

Our rationale that there already is compelling evidence supporting the strong biomaterial potential of Tintinnidorin-based shell material is presented in Supplementary Note 2 (“Properties of the shell biomaterial”):

The primary scope of the present study is the discovery and first characterization of Tintinnidorin proteins. Several remarkable properties of tintinnid shells have been experimentally documented, clearly demonstrating the potential of this proteinaceous biomaterial. Agatha & Simon (2012) demonstrated that the shell-forming material of tintinnids with diverse shells exhibits remarkable resistance to high temperatures (about 160° C), strong acids and bases (hydrofluoric acid and potassium hydroxide), and various enzymatic treatments. Further enzymatic digestion experiments were conducted in preliminary analyses of the present study (Supplementary Method 4).

During our live observations, just secreted Tintinnidorin granules frequently got in contact with the glass slide and were not removed by the vigorously fidgeting tintinnid cells (Ganser et al. 2025). While sticking on the slide, the material granules showed an expansion and merging, forming a material layer with small chambers resembling the natural shell wall texture (Supplementary Movie 1). Wet adhesion (adhesion of structures in the presence of liquids) is also displayed by agglutinated tintinnid shells, in which the shell-forming material secreted by the ciliate adheres foreign particles with different properties (calcium carbonate/calcite: coccoliths and mineral particles; silicate: diatom frustules and mineral particles). In Tintinnidorin, the most plausible explanation for wet adhesion are molecular interactions of the highly abundant tyrosine and lysine, as in marine animal biomaterials (e.g., mussel foot proteins). We consider these examples sufficient evidence for the Tintinnidorin’s wet adhesive properties, although dedicated assays were not conducted.

The shell biomaterial self-assembly was also captured in a video of lorica formation in free-swimming *Schmidingerella* tintinnids (Ganser et al. 2025) and mentioned in previous light microscopical studies (Laval-Peuto 1981). The capacity of the shell biomaterial to generate a considerable variety of layered wall ultrastructures is demonstrated by the diverse shell textures (Figure 1).

The tintinnid shell biomaterial likely exhibits photoprotective properties against ultraviolet radiation as indicated by the high content of aromatic amino acids in Tintinnidorin and the intrinsic autofluorescence of tintinnid shells (Supplementary Fig. 5).

Future studies investigating sequence-structure-material relationships, particularly using recombinantly expressed proteins, will be key to determine how Tintinnidorin properties can be reproduced and potentially tailored for specific biomaterial applications. Thereby, the natural variability displayed by the Tintinnidorin might even be enlarged.

In the manuscript, we introduce the outline above in lines 414-419: “The potential of the tintinnid shell biomaterial has been demonstrated by experiments and observations revealing remarkable properties, such as thermal and chemical resistance, photoprotection, wet adhesion, and self-assembly (Supplementary Note 2). On the molecular level, Tintinnidorin proteins represent a biomaterial with novel architectures that could serve as a convenient model complementing current biomaterial research.”

2. The linkers are proposed to be intrinsically disordered and critical for phase separation, but they are poorly covered in the mass spectrometry data. The authors mention possible reasons (detection limits or crosslinking). A more detailed discussion—or ideally, additional experiments using alternative digestion protocols—would help clarify whether this is a technical limitation or reflects a genuine structural feature.

ANSWER:

We provide a more detailed discussion in Supplementary Method 5 (“Interpretation of proteomic data”):

Combining single-cell transcriptomics and shell proteomics was key to obtain the full-length Tintinnidorin protein sequences and directly verify their presence in the shell biomaterial. To achieve this, several non-standard digestion approaches were employed, using both unspecific and more specific proteases (proteinase K, elastase, and trypsin). As these experiments represent the first proteomic analyses of tintinnid shell material, particular care was taken to use protease concentrations that were as low as possible relative to the unknown protein content of the shells. Proteinase K and elastase yielded promising results in preliminary tests. For these enzymes, we empirically determined the minimal concentration at which gradual shell decomposition could be visually confirmed by light microscopy. In contrast, several other proteases (e.g., α-chymotrypsin, trypsin, protease XIV) did not show detectable effects on the shells of *Schmidingerella*, even though potential cleavage sites for these proteases are present throughout the protein sequences, including modules, linkers, and terminal domains.

How Tintinnidorin proteins assemble into the shell biomaterial and how they are ultimately arranged within the hardened shell wall remains unknown. The architecture of this resistant proteinaceous biomaterial determines the accessibility of proteolytic cleavage sites, which depends on several structural factors that cannot be readily inferred from peptide fragmentation patterns in the mass spectra. Consequently, standard assumptions derived from the digestion efficiency and cleavage patterns of soluble proteins are of limited applicability to such a biomaterial.

Because the shell proteins are novel to science, we employed a non-standard proteomics workflow in which peptide identification from mass spectra relied on a reference-free de novo approach rather than conventional database-driven matching. Peptide identification was likely further impeded by intra- and intermolecular crosslinking, as well as extensive proteolytic digestion, which may generate atypical fragmentation patterns and very small

peptide products, respectively. Even when the masses of such atypical fragments were detected, the corresponding spectra often could not be interpreted.

While the current experiments provide direct evidence for the identity of the shell biomaterial proteins, detailed structural inferences will require different approaches in future studies.

3. Overinterpretation of some claims. Two examples stood out: the statement that Tintinnidorin proteins “maintain self-assembly capacity across a wide range of salinities” is inferred from species distribution but not directly tested. Similarly, the term “wet adhesion” is used without supporting adhesion assays. I recommend tempering these claims or adding appropriate caveats.

ANSWER:

To temper the claim regarding the self-assembly capacity, we have rephrased lines 347-353: “In comparison to these animals, tintinnid ciliates cannot actively control the extracellular physicochemical environment during shell formation as the shell-forming material is rapidly secreted into the surrounding (sea-)water. Nevertheless, family and genus-specific shell characteristics are consistent across widely differing environmental conditions. In some genera, species have even been recorded from seawater, brackish water, and freshwater. How Tintinnidorin proteins reliably self-assemble despite such contrasting conditions remains an open question (Supplementary Note 7).”

In the answer to the first remark regarding material properties (please see above), we argue why think that the use of the term “wet adhesion” is appropriate for the tintinnid shell biomaterial.

Details regarding known extracellular conditions and their influence on tintinnid shells are provided in Supplementary Note 7 (“Influence of ionic composition on material assembly”):

In the seawater, the major ions show constant ratios in the salinity range of about 33-37 ‰ (Forchhammer's Principle; Dittmar 1884). In brackish waters, the ion composition fluctuates due to freshwater influence and biological processes. The total salinity concentration affects the distribution ranges of tintinnid species, although euryhaline estuarine species are occasionally recorded (Dolan & Gallegos 2001). A much larger tolerance concerning salinity gradients is, however, demonstrated by genera recorded in the Red Sea at about 40 ‰ and the brackish waters of the Baltic Sea (Dolan & Pierce 2013). Extreme examples are the genera *Codonella*, *Stenosemella*, and *Tintinnidium*, which even occur in freshwater lakes, still forming the genus-specific shells. In the present study, we have identified 78 full-length Tintinnidorin proteins from single-cell and metatranscriptome data. Congruently with microscopic data, these findings indicate a global distribution of tintinnids. If Tintinnidorin self-assembly depends on ions, they are thus apparently universally available in sufficient concentrations or can be replaced by equivalent ions. Future studies have to answer the question of how Tintinnidorin proteins manage self-assembly under contrasting conditions.

Minor points:
1. Some figures are dense and difficult to read, particularly Fig. 4 and Extended Data Fig. 3. Simplifying these or splitting them into multiple panels would improve accessibility.

ANSWER:

Author guidelines prescribe a font size of 5-8 pt and an arrangement of several plots into single figures. To keep the results shown in the plots thematically coherent, we have partly combined Figures 4 and 5 and moved two plots (formerly Fig. 4 b,c) to Supplementary Fig. 4.

The quality and resolution of the images should allow a zooming into the figure to facilitate accessibility.

2. In all result figures, placing the label of each sub-figure in the top-left corner would be more consistent with standard reading habits.

ANSWER

Sub-figure labels are now in the top-left corner in the respective result figures.

3. A glossary of specialist terms (e.g., LLPS, pLDDT) would be useful for readers outside structural biology or biomaterials.

ANSWER:

We have added a brief explanation in Supplementary Note 6: “We interpret these transitions as successive stages of a liquid–liquid phase separation (LLPS) process, in which two distinct phases are separated because of changing physicochemical conditions.”

Generally, the journal’s guidelines do not intend to add a glossary in the main part of the manuscript. Acronyms and abbreviations should be defined the first time they appear, e.g. liquid-liquid phase separation (LLPS).

4. The discussion of evolutionary mechanisms (e.g., alternative processing, gene conversion) is brief. Expanding this section would strengthen the evolutionary narrative.

ANSWER:

Currently, inferences about specific evolutionary mechanisms are limited due to the lack of tintinnid genome data, which we now refer to in lines 285-287: “Due to the lack of a complete Schmidingerella reference genome including micronuclear and macronuclear data, and of tintinnid genomic data in general, the evolutionary mechanisms remain elusive (Supplementary Note 5).”

A more detailed account, based on the limited available data, is given in Supplementary Note 5 (“Supposed evolutionary mechanisms”):

The evolutionary mechanisms discussed for the tintinnids are derived from features related to the special genome architecture of ciliates, which possess two types of nuclei with different functions (nuclear dimorphism). This architecture is associated with elevated rates of protein evolution, especially in ciliates with extensively fragmented genomes, such as tintinnids (Smith et al. 2020; Zufall et al. 2006). An extensive genome processing of the germline micronucleus during macronuclear development after conjugation produces thousands of gene-sized nanochromosomes in tintinnids and close relatives. During this process, micronuclear sequences are rearranged through the excision of internal eliminated sequences (IESs) and the assembly of macronuclear-destined sequences (MDSs), sometimes involving a complex genome scrambling (e.g., Maurer-Alcalá and Nowacki 2019; Smith et al. 2022). Such mechanisms are suggested to promote rapid sequence diversification and the expansion of lineage-specific gene families (Maurer-Alcalá et al. 2024). In this context, the diversification of Tintinnidorin proteins may reflect repeated duplication and rearrangement events in the micronuclear genome followed by alternative processing into macronuclear nanochromosomes, potentially accounting for the large diversity of shells observed among tintinnids. However, detailed evolutionary hypotheses are difficult to formulate because genomic data from both the micronuclei and macronuclei are

currently incomplete for *Schmidingerella* (Smith et al. 2020) and entirely missing for other tintinnids. High-quality assemblies of these two nuclear genomes from a single species are necessary to determine how Tintinnidorin genes are organized in the micronuclear genome and how they are processed into macronuclear nanochromosomes. Thereby, the evolutionary patterns underlying the diversification of this protein family will be elucidated.
